# The Mechanism of Dehydrating Bimodules in *trans*‐Acyltransferase Polyketide Biosynthesis: A Showcase Study on Hepatoprotective Hangtaimycin

**DOI:** 10.1002/anie.202106250

**Published:** 2021-07-28

**Authors:** Minghe Luo, Houchao Xu, Yulu Dong, Kun Shen, Junlei Lu, Zhiyong Yin, Miaomiao Qi, Guo Sun, Lingjie Tang, Jin Xiang, Zixin Deng, Jeroen S. Dickschat, Yuhui Sun

**Affiliations:** ^1^ Key Laboratory of Combinatorial Biosynthesis and Drug Discovery Ministry of Education, and School of Pharmaceutical Sciences Wuhan University No. 185 East Lake Road Wuhan 430071 People's Republic of China; ^2^ Kekulé-Institute for Organic Chemistry and Biochemistry University of Bonn Gerhard-Domagk-Straße 1 53121 Bonn Germany

**Keywords:** biosynthesis, dehydrating bimodules, natural products, polyketide synthases, *trans*-acyltransferase

## Abstract

A bioassay‐guided fractionation led to the isolation of hangtaimycin (HTM) from *Streptomyces spectabilis* CCTCC M2017417 and the discovery of its hepatoprotective properties. Structure elucidation by NMR suggested the need for a structural revision. A putative HTM degradation product was also isolated and its structure was confirmed by total synthesis. The biosynthetic gene cluster was identified and resembles a hybrid *trans*‐AT PKS/NRPS biosynthetic machinery whose first PKS enzyme contains an internal dehydrating bimodule, which is usually found split in other *trans*‐AT PKSs. The mechanisms of such dehydrating bimodules have often been proposed, but have never been deeply investigated. Here we present in vivo mutations and in vitro enzymatic experiments that give first and detailed mechanistic insights into catalysis by dehydrating bimodules.

## Graphical abstract

Hangtaimycin biosynthesis proceeds through a hybrid *trans*‐AT PKS/NRPS biosynthetic machinery that contains distinctive dehydrating bimodules. Here we present detailed mechanistic insights into catalysis by dehydrating bimodules that are generally involved in the biosynthesis of polyketides with *Z* double bonds. This work lays the foundations for future structural modifications by protein engineering.

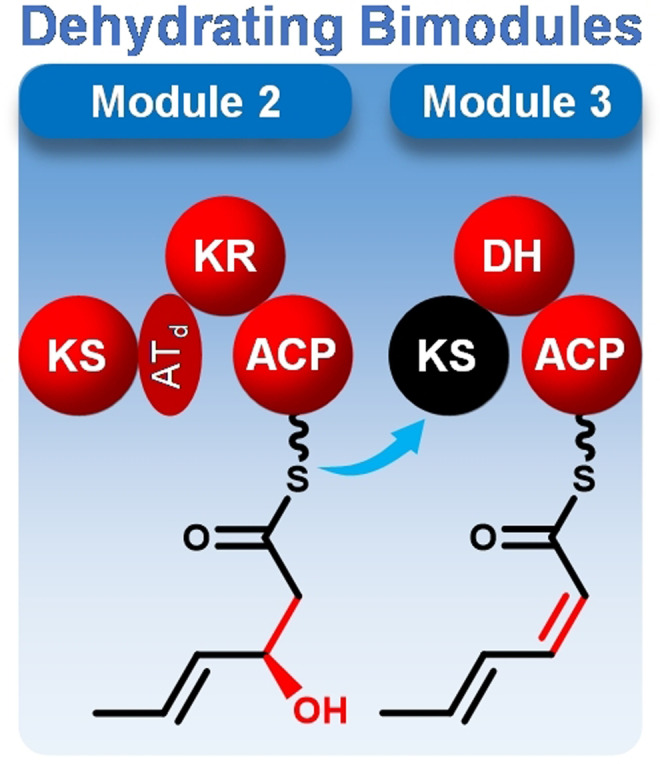

For many years *Actinomycetes* have been a major source for the discovery of bioactive natural products and the development of new drugs such as the antibiotic erythromycin, the immunosuppressant rapamycin, or the antitumor antibiotic bleomycin.[^1^, ^2^] As an important “detoxification” organ the liver plays an important role in human metabolism.^[3]^ Liver injury may cause fatigue, coagulopathy, encephalopathy and liver failure, which are threating people's lives, but only a few hepatoprotective drugs are clinically available for specific applications, for example, acetylcysteine is used to treat paracetamol overdosage and silymarin, a lipophilic extract from milk thistle (*Silybum marianum*) seeds, is administered in liver injuries by carbon tetrachloride, alcohol, in chronic viral hepatitis or acute poisonings with *Amanita* mushrooms.^[4]^ The limited availability of hepatoprotective drugs raises an urgent need for the discovery of new compounds.

In the process to screen hepatoprotective drug leads, crude culture extracts from *Streptomyces spectabilis* CCTCC M2017417^[5]^ revealed an interesting activity by inhibiting the increase of alanine (ALT) and aspartate aminotransferase (AST) activities in the human hepatoma cell line HepG2 stimulated with CCl_4_, which are indicators of liver overfunction.^[6]^ A subsequent bioassay guided fractionation led to the isolation of hangtaimycin (HTM, **1**) as the active principle (Figure S1). This known compound^[7]^ was identified by ESI‐HRMS and NMR spectroscopy (Figures 1, S2–S9 and Table S3). However, a detailed inspection of the NMR spectra indicated an *E* configured Δ^29,30^ double bond instead of the published *Z* configuration,^[7]^ especially because of the absence of a ROESY correlation between H29 and H30 in conjunction with the signal multiplicity for H30 (dd, *J=*15.0, 10.2 Hz). The ^1^H‐NMR signals for H29 and H31 are overlapping with other signals, which prevents a reverse assignment of coupling constants, but *J=*15.0 Hz must be relevant for the coupling over an olefinic double bond (between H29 and H30, thus revealing its *E* configuration), while *J=*10.2 Hz must be assigned to the coupling between H30 and H31 over a formal single bond. Besides HTM also a putative degradation product was detected by LC‐ESI‐HRMS ([M+H]^+^ *m*/*z* 223.14349, calcd for C_12_H_18_O_2_N_2_^+^ *m*/*z* 223.14410), which corresponds to the hemiaminal cleavage product HTM_222_ (index 222 referring to its molecular mass, Scheme S1a, Figure 1). This compound was synthesized from l‐alanine (Scheme S1b, Figures S10–S48), and the natural product and the synthetic material showed identical chromatographic behaviour and mass spectra (Figure S49). These findings also confirmed the assigned 2*Z*,4*E* configuration in the sorbic acid amide portion of HTM. HTM_222_ has been isolated before from *Streptomyces* sp. SBI108 and named sarmentosamide^[8]^ which may also arise by degradation of HTM or a similar metabolite in this organism. The optical rotation of the synthetic compound, [*α*]25D =−194 (*c* 0.28, MeOH), shows the same sign as the natural product, [*α*]28D =−141 (*c* 0.05, MeOH).^[8]^

**Figure 1:**
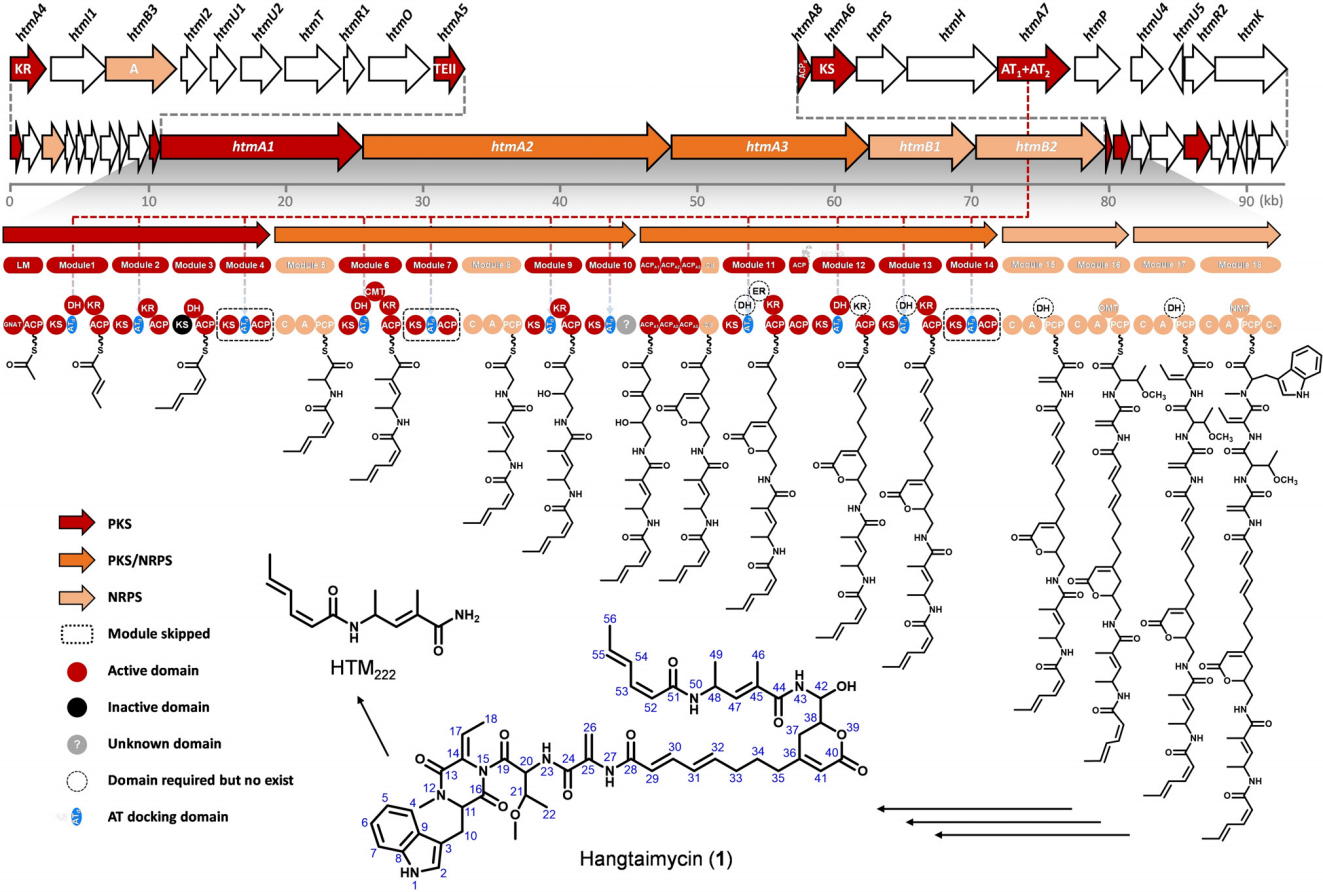
Structure of hangtaimycin (**1**) and its biosynthetic gene cluster. Proposed functions for individual ORFs are summarized in Table S2.

HTM is the product of a hybrid polyketide synthase/nonribosomal peptide synthetase (PKS/NRPS). According to the classical assembly line paradigm of PKS/NRPS, for the modular type of these enzymes usually a colinearity logic exists with one module being responsible for the incorporation of one unit,^[9]^ while the domain organization with acyl carrier proteins (ACP) for binding of intermediates, acyl transferase (AT) for upload of an extender unit, ketosynthases (KS) for catalysis of chain elongations by decarboxylative Claisen condensations, and optional ketoreductase (KR), dehydratase (DH) and enoyl reductase (ER) domains for *β*‐keto group processing allows for detailed functional predictions.^[9]^ In contrast, *trans*‐AT PKSs contain a much more complex and less understood domain architecture, often with split modules, unfunctional or repetitive domains or even modules, and domains with special functions only seen in one or few cases.[^10^, ^11^]

Bioinformatic analysis of the genome sequence of *S. spectabilis* CCTCC M2017417 resulted in the discovery of a putative biosynthetic gene cluster with 74.4 % G+C content that could explain the structure of HTM (Table S4). Deletion of the entire cluster from *htmA4* to *htmK* resulted in an abolished HTM production, confirming its function as HTM biosynthetic gene cluster (Figure S50). The central part encodes five multidomain enzymes comprising a *trans*‐AT[^18^, ^19^] PKS/NRPS hybrid machinery (Table S4) with an integral flanking subdomain of 90–110 amino acids located immediately after each KS except the loading module and module 3 to which the discrete duplicate AT encoded by *hmtA7* might dock (Figure 1). Another interesting observation are missing KR and DH domains in modules 11–13 that would be needed to explain the HTM structure. Here also KR and/or DH domains may act *in trans*. In‐frame deletion of *htmA7* completely abolished HTM production, verifying its essential role as an *in trans* acting AT for HTM biosynthesis (Figure S51).

More strikingly, an unusual modular organization was found for HtmA1, in which—according to the structure of HTM and against classical PKS colinearity—modules 1–4 are responsible for the elongation of the acetyl‐CoA starter with only two malonyl‐CoA units. Modules 2 and 3 together show a domain organization that closely resembles that of a type A dehydrating bimodule. Such bimodules are usually split after the second non‐elongating KS^0^ domain and generally show the domain organization KS‐KR‐ACP‐KS^0^// DH‐ACP and are believed to catalyze the installation of a *Z* double bond.^[12]^ Type B dehydrating bimodules are also split (KS‐KR‐ACP‐KS// DH‐ACP‐KR), but here both KS domains are elongating and the DH catalyzes two eliminations of water with formation of a 2*E*,4*Z* diene.^[13]^ Dehydrating bimodules are a special feature of *trans*‐AT PKSs and occur inter alia in the biosynthetic machineries for bacillaene,^[14]^ difficidin,^[15]^ batumin,^[16]^ kirromycin^[17]^ and gladiolin.^[18]^ For the dehydrating bimodule in the gladiolin PKS the importance of a docking domain for protein‐protein interaction and efficient acyl group transfer has been demonstrated in vitro,^[19]^ but further aspects of catalysis by dehydrating bimodules have not been studied so far. This prompted us to aim at a deep investigation of the hypothetical integral dehydrating bimodule of HtmA1.

Alternative biosynthetic hypotheses for the biosynthesis of the (2*Z*,4*E*)‐sorbic acid portion made up by C51–C56 in HTM are shown in Figure 2. The starter unit is likely malonyl‐CoA that is uploaded to the loading module with decarboxylation by the GNAT domain.^[20]^ This step must be followed by two chain extensions with double bond formation, but the only module in HtmA1 that is fully equipped for double bond installation is module 1. In order to test whether module 1 functions iteratively to perform two rounds of chain extension (Figure 2 a), individual in vivo inactivations of DH_1_ and KR_1_ (the index refers to the module number) by mutating active site and NADPH binding sites, respectively (Figures S52 and S53). LC‐ESI‐HRMS analysis of culture extracts from the DH_1_(H26A) and KR_1_(G10Q, G12L, G15A) mutants showed a disrupted HTM production, but also no other product such as a derivative with hydroxyl or keto groups at C53 and C55 was observed. At this stage an iterative function of module 1 could still not completely be ruled out, because it is possible that downstream modules do not take over such hydroxyl‐ or ketoacyl intermediates from ACP_1_, but this hypothesis also seems unlikely, because one and the same set of KR and DH domains would need to be responsible for the installation of one *E* and one *Z* double bond.

**Figure 2:**
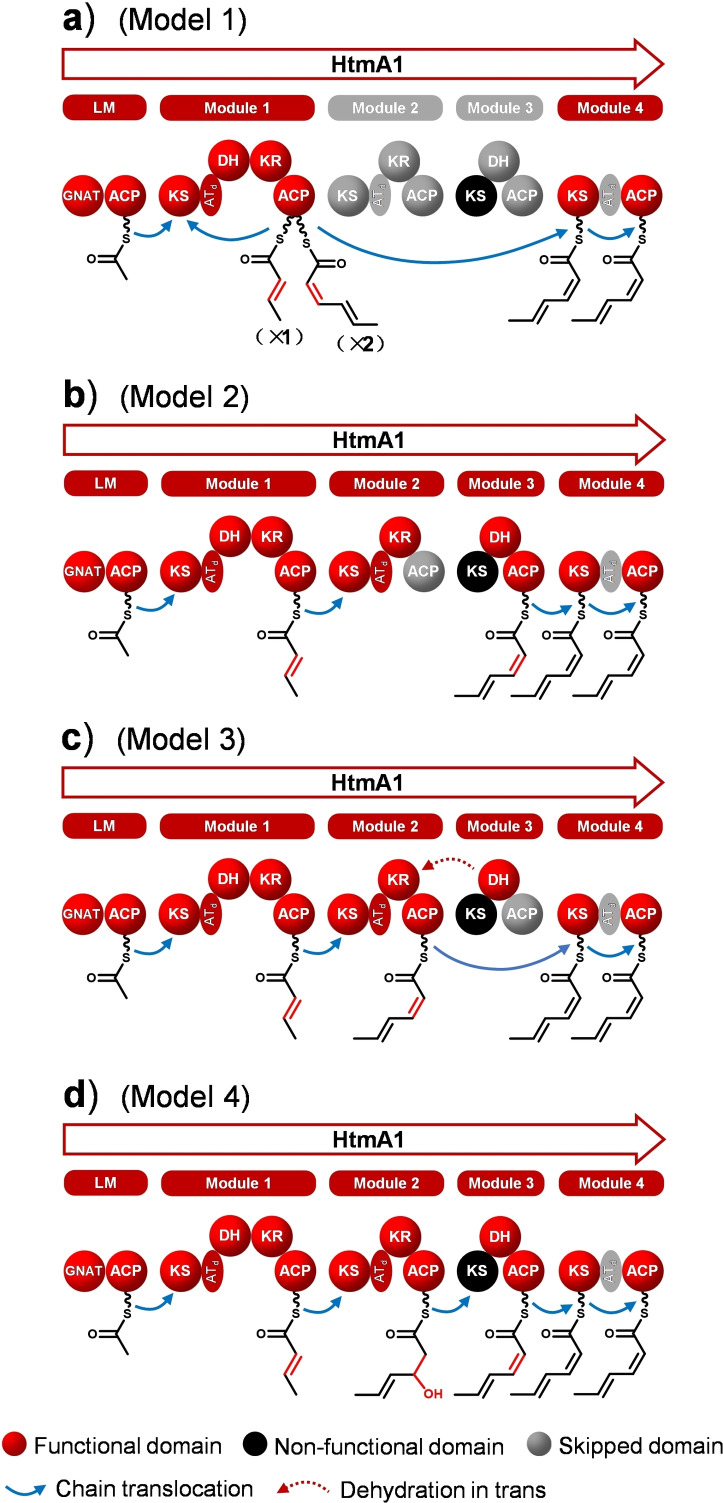
Alternative models for triketide biosynthesis by HtmA1. a) Module 1 iteratively catalyzes two elongations with skipping of modules 2 and 3. b) Modules 2 and 3 represent an active dehydrating bimodule with processing of the acyl group solely bound to ACP_3_ (ACP_2_ is skipped), c) solely bound to ACP_2_ (ACP_3_ is skipped), or d) bound to ACP_2_ for chain extension and ketoreduction and to ACP_3_ for dehydration.

Similar to the situation in type A dehydrating bimodules, HtmA1 KS_3_ is predicted to be inactive with respect to chain elongation based on the observed Cys‐**Asn**‐His motif instead of the usual highly conserved Cys‐**His**‐His catalytic triad,^[21]^ but because of the retained Cys in this motif KS_3_ could still act as a transacylase for passing on the substrate to a downstream ACP (Figure S54). Instead of an iteration of module 1 and as suggested for (split) dehydrating bimodules, modules 2 and 3 could cooperate in one chain elongation with formation of a *Z* olefin. This may proceed with attachment of the triketide intermediate solely to ACP_3_, i. e. with a silent ACP_2_ and KR_2_ acting on the ACP_3_ bound intermediate (Figure 2 b). Alternatively, the triketide intermediate may still be tethered on ACP_2_ after ketoreduction by KR_2_ and dehydration by DH_3_ “*in‐trans‐like*” acting on the ACP_2_ bound intermediate, followed by module 3 skipping (Figure 2 c). Finally, chain elongation to the triketide and ketoreduction could be performed at ACP_2_, followed by KS_3_‐mediated transacylation to ACP_3_ and dehydration by DH_3_ (Figure 2 d), as it is generally proposed for type A dehydrating bimodules, but has never been verified experimentally.

To further examine the above hypotheses, site‐directed mutations were introduced into the active sites of KR_2_ and DH_3_, yielding the mutant strains KR_2_(Y168A) and DH_3_(H25A) (Figures S55 and S56). Both mutants neither produced HTM nor a new derivative with a hydroxyl or keto group at C53, revealing the involvement of KR_2_ and DH_3_ in HTM biosynthesis and thus ultimately ruling out the possibility of an iterative usage of module 1 (Figure 2 a). To distinguish between models 2, 3 and 4, site‐directed mutation was performed at ACP_2_ and ACP_3_, yielding the in vivo mutants ACP_2_(S40A, I41F) and ACP_3_(D43L, S44A), respectively (Figures S57 and S58). LC‐ESI‐HRMS analysis of culture extracts showed an abolished HTM biosynthesis in both cases, indicating the involvement of ACP_2_ and ACP_3_ in HTM biosynthesis. These results disfavor models 1–3 and support model 4. Furthermore, also the site‐directed mutation of the transacylation site of KS_3_ (KS_3_(C155A), Figure S59) abolished HTM production, which further supports model 4. In particular, the negative HTM biosynthesis by KS_3_(C155A) suggests that KS_3_ plays a role as a “springboard” in the intermediate transfer from ACP_2_ to ACP_3_.

A detailed mechanistic hypothesis for the cooperativity of modules 2 and 3 is shown in Figure S60. This mechanistic model was further investigated by in vitro enzymatic reactions. Modules 1, 2 and 3 of HtmA1 were expressed as recombinant multidomain proteins in *Escherichia coli* BAP1 and purified (Figure S61). This strain contains the *sfp* gene under control of the T7 promoter leading to efficient phosphopantetheinylation of carrier proteins (*holo*‐CPs).^[22]^ For loading of the malonyl‐CoA extender unit, HtmA7 was also expressed in *E. coli* BL21(DE3) containing plasmid pGro7 with GroEL/ES chaperone (Figure S61).^[23]^ As a substrate surrogate the *N*‐acetylcysteamine thioester (SNAC ester) of crotonic acid, (*E*)‐2‐butenoyl‐SNAC (**2**), was synthesized (Figures S62–S64) and incubated with *holo*‐module 1 in the presence of malonyl‐CoA and NADPH, followed by alkaline hydrolysis of the thioester‐bound products.[^17^, ^24^] No chain elongation product (**3**) was detected (Figure 3 a), confirming again in vitro that module 1 is not used iteratively. Incubation of *holo*‐module 2 with malonyl‐CoA and NADPH, alkaline workup and LC‐ESI‐HRMS analysis revealed formation of a single product that showed an identical chromatographic behaviour and mass spectrum as authentic (*E*)‐3‐hydroxyhex‐4‐enoic acid (**3**), demonstrating that module 2 catalyzes one chain elongation step with ketoreduction (Figures 3 b,h). The absolute configuration of **3** was determined by synthesis of (*rac*)‐**3** and enantiomerically enriched (*R*)‐**3** by kinetic resolution through Sharpless epoxidation of ethyl (*rac*)‐3‐hydroxyhex‐4‐enoate and saponification (Scheme S2, Figures S65–S67).^[25]^ HPLC analysis of the synthetic materials and the enzyme product using a chiral stationary phase demonstrated *R* configuration for enzymatically generated **3** (Figure S68). In a second approach, enantiomerically pure (*R*)‐ and (*S*)‐**3** were prepared by using the Evans oxazolidinone method (Scheme S3).^[26]^ As the assignment for the absolute configurations to the enantiomers of **3** is not very well explained in the literature, their absolute configurations were independently confirmed by chemical correlation with (*S*)‐ and (*R*)‐3‐hydroxyhexanoic acid by catalytic hydrogenation.[^26^, ^27^]

**Figure 3:**
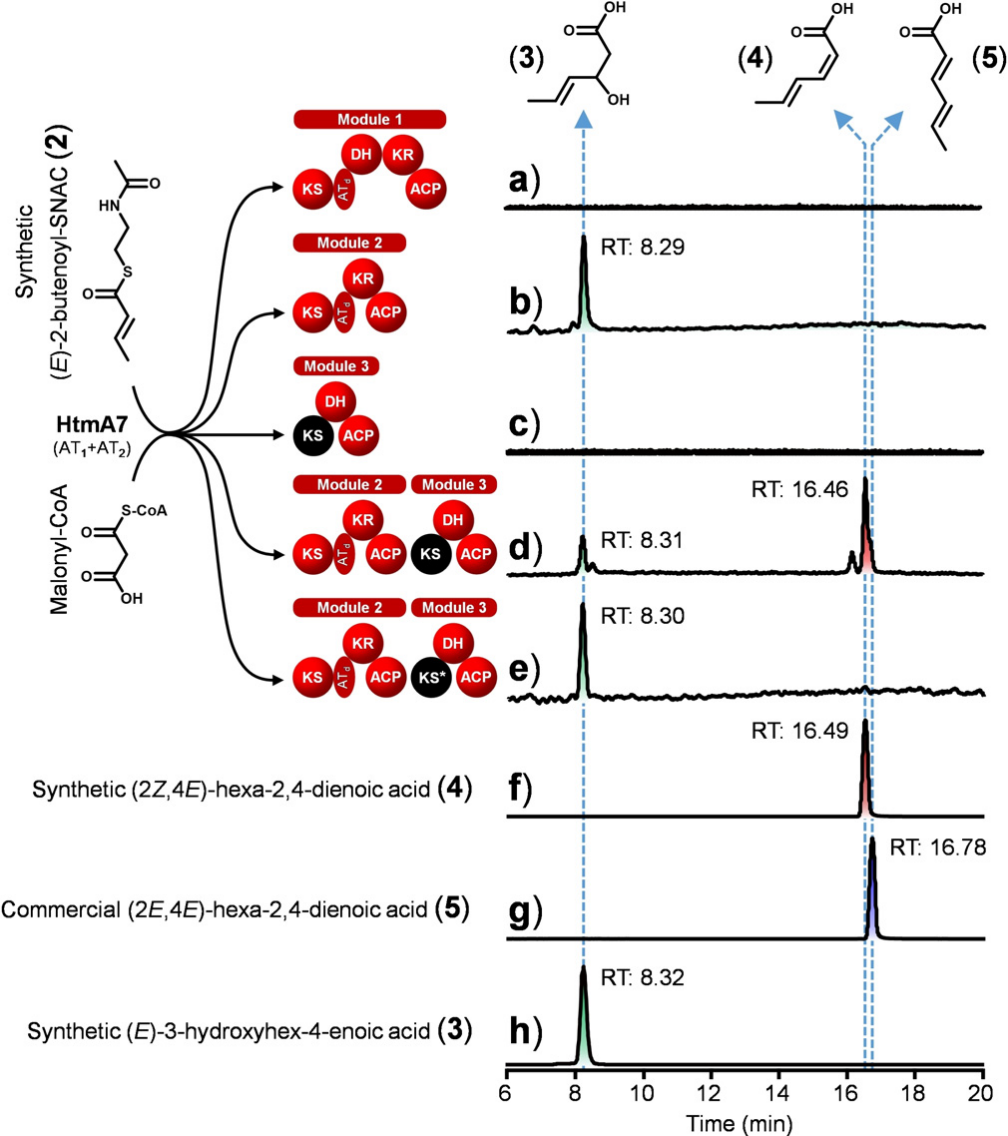
In vitro reconstitution of triketide biosynthesis by HtmA1. The SNAC ester **2** serves as a substrate mimic of module 2 and is condensed with one unit of malonyl‐CoA. The extension products were released from HtmA1 by alkaline hydrolysis and monitored by LC‐ESI‐HRMS. The KS_3_ domain that is unable to catalyze chain extension and only acts as a transacylase is shown in black. The KS_3_ domain fully inactivated by site‐directed mutation on the transacylation active site (C155A) is indicated by an asterisk. Synthetic **3** and **4** and commercial **5** were used as standards for compound identification.

When the same reaction was done in the presence of *holo*‐module 2 plus *holo*‐module 3, a major compound identical to authentic (2*Z*,4*E*)‐hexa‐2,4‐dienoic acid (**4**) was produced, confirming chain elongation with ensuing ketoreduction and dehydration (Figure 3 d). The retention time of the product (16.46 min) closely matched that of synthetic **4** (16.49 min, Figure 3 f), while a small, but noticeable difference was observed for the retention time of commercial (2*E*,4*E*)‐hexa‐2,4‐dienoic acid (**5**, 16.78 min, Figure 3 g), clearly proving *Z* configuration of the newly installed double bond in the triketide, as observed in HTM and HTM_222_. Product **4** was not detected with *holo*‐module 3 alone (Figure 3 c), showing that module 3 can take over the intermediate polyketide chain from module 2 and subsequently dehydrate the hydroxyl group, but cannot condense and extend the carbon chain. Consistent with the corresponding in vivo results, the combination of wild‐type *holo*‐module 2 and the *holo*‐module 3‐KS_3_(C155A) enzyme variant obtained by site‐directed mutation made the in vitro extension of module 3 impossible (Figure 3 e, Figure S59), which excludes model 3 (Figure 2).

Taken together, HTM biosynthesis is achieved by a hybrid *trans*‐AT PKS‐NRPS and starts with the formation of a triketide by HtmA1 with involvement of an internal type A dehydrating bimodule. Such dehydrating bimodules are frequently observed in *trans*‐AT PKSs but are usually split over two proteins after the second KS domain. Apart from the recently reported importance of a docking domain for efficient cooperation of these split didomains^[19]^ not much was known about the mechanism of dehydrating bimodules, although their function has often been hypothesized in previous reports. We here show by in vivo mutations and in vitro enzyme reactions how they act: The first module (KS‐KR‐ACP) takes over the acyl group from the preceding module with its KS, followed by decarboxylative Claisen condensation with an ACP‐bound malonate unit and ketoreduction. The KS^0^ of the second module, composed of KS^0^‐DH‐ACP, is a non‐elongating transacylase and passes on the acyl group to the ACP, followed by dehydration. Dehydrating bimodules usually contain A‐type ketoreductases,^[28]^ which introduce *R* configured hydroxyl groups. In cooperation with dehydratases these systems are generally involved in the biosynthesis of polyketides with *Z* double bonds.[^12^, ^13^, ^29^]

## Conflict of interest

The authors declare no conflict of interest.

## Supporting information

As a service to our authors and readers, this journal provides supporting information supplied by the authors. Such materials are peer reviewed and may be re‐organized for online delivery, but are not copy‐edited or typeset. Technical support issues arising from supporting information (other than missing files) should be addressed to the authors.

Supporting Information
