# Supplementary material for: The Mechanism of Dehydrating Bimodules in trans‐Acyltransferase Polyketide Biosynthesis: A Showcase Study on Hepatoprotective Hangtaimycin
Source: Angew Chem Int Ed Engl. 2021 Jul 28;60(35):19139–43. doi: 10.1002/anie.202106250 (PMC8456789; doi:10.1002/anie.202106250)
Supplement: Supplementary file 1 — Supporting Information [file ANIE-60-19139-s001.pdf]

Angewandte Chemie
Eine Zeitschrift der Gesellschaft Deutscher Chemiker

Supporting Information

The Mechanism of Dehydrating Bimodules in *trans*-Acyltransferase Polyketide Biosynthesis: A Showcase Study on Hepatoprotective Hangtaimycin

Minghe Luo[+], Houchao Xu[+], Yulu Dong, Kun Shen, Junlei Lu, Zhiyong Yin, Miaomiao Qi, Guo Sun, Lingjie Tang, Jin Xiang, Zixin Deng, Jeroen S. Dickschat, and Yuhui Sun**

anie_202106250_sm_miscellaneous_information.pdf

Supporting Information

Table of Contents

Table S1. Bacterial strains and plasmids used in this study....4
Table S2. List of oligonucleotide primers used in this study 5
Figure S1. Hepatoprotective effects of HTM (**1**) against damage induced by CCl_4 in HepG2 cells 8
Table S3. ^{13}C (150 MHz) and ^{1}H (600 MHz) NMR data for HTM (**1**) 10
Figure S2. Key COSY and HMBC correlation of HTM (**1**)....11
Figure S3. ESI-HRMS spectrum of HTM (**1**)....12
Figure S4. ^{1}H NMR spectrum of HTM (**1**) (600 MHz, $(CD_3)_2SO$) 12
Figure S5. ^{13}C NMR spectrum of HTM (**1**) (150 MHz, $(CD_3)_2SO$)....13
Figure S6. HSQC spectrum of HTM (**1**) (600 MHz, $(CD_3)_2SO$)....13
Figure S7. ^{1}H-^{1}H COSY spectrum of HTM (**1**) (600 MHz, $(CD_3)_2SO$) 14
Figure S8. HMBC spectrum of HTM (**1**) (600 MHz, $(CD_3)_2SO$)....14
Figure S9. ROESY spectrum of HTM (**1**) (600 MHz, $(CD_3)_2SO$)....15
Scheme S1. a) Putative degradation of hangtaimycin to sarmentosamide (HTM_{222}). b) Synthesis of sarmentosamide....16
Figure S10. ^{1}H NMR spectrum (500 MHz, CD_3OD) of **S2** 21
Figure S11. ^{13}C NMR spectrum (126 MHz, CD_3OD) of **S2** 21
Figure S12. ^{13}C-DEPT-135 NMR spectrum (126 MHz, CD_3OD) of **S2**....22
Figure S13. ^{1}H NMR spectrum (500 MHz, $CDCl_3$) of **S3**....22
Figure S14. ^{13}C NMR spectrum (126 MHz, $CDCl_3$) of **S3**....23
Figure S15. ^{13}C-DEPT-135 NMR spectrum (126 MHz, $CDCl_3$) of **S3**....23
Figure S16. ^{1}H NMR spectrum (500 MHz, $CDCl_3$) of **S4**....24
Figure S17. ^{13}C NMR spectrum (126 MHz, $CDCl_3$) of **S4**....24
Figure S18. ^{13}C-DEPT-135 NMR spectrum (126 MHz, $CDCl_3$) of **S4**....25
Figure S19. ^{1}H NMR spectrum (500 MHz, CD_3OD) of **S5**....25
Figure S20. ^{13}C NMR spectrum (126 MHz, CD_3OD) of **S5**....26
Figure S21. ^{13}C-DEPT-135 NMR spectrum (126 MHz, CD_3OD) of **S5**....26
Figure S22. ^{1}H NMR spectrum (700 MHz, $CDCl_3$) of **S12**....27
Figure S23. ^{13}C NMR spectrum (176 MHz, $CDCl_3$) of **S12**....27
Figure S24. ^{13}C-DEPT-135 NMR spectrum (176 MHz, $CDCl_3$) of **S12**....28
Figure S25. ^{1}H NMR spectrum (700 MHz, $CDCl_3$) of **4**....28
Figure S26. ^{13}C NMR spectrum (176 MHz, $CDCl_3$) of **4**....29
Figure S27. ^{13}C-DEPT-135 NMR spectrum (176 MHz, $CDCl_3$) of **4**....29
Figure S28. ^{1}H NMR spectrum (500 MHz, CD_3OD) of **S7**....30
Figure S29. ^{13}C NMR spectrum (126 MHz, CD_3OD) of **S7**....30
Figure S30. ^{13}C-DEPT-135 NMR spectrum (126 MHz, CD_3OD) of **S7**....31
Figure S31. ^{1}H-^{1}H COSY spectrum (500 MHz, CD_3OD) of **S7**....31
Figure S32. HSQC spectrum (CD_3OD) of **S7**....32
Figure S33. HMBC spectrum (CD_3OD) of **S7**....32
Figure S34. NOESY spectrum (CD_3OD) of **S7**....33
Figure S35. ^{1}H NMR spectrum (500 MHz, CD_3OD) of **S8**....33
Figure S36. ^{13}C NMR spectrum (126 MHz, CD_3OD) of **S8**....34
Figure S37. ^{13}C-DEPT-135 NMR spectrum (126 MHz, CD_3OD) of **S8**....34
Figure S38. ^{1}H-^{1}H COSY spectrum (500 MHz, CD_3OD) of **S8**....35

Figure S39. HSQC spectrum (CD_3OD) of **S8**......35
Figure S40. HMBC spectrum (CD_3OD) of **S8**......36
Figure S41. NOESY spectrum (CD_3OD) of **S8**......36
Figure S42. ^{1}H NMR spectrum (700 MHz, CD_3OD) of HTM_{222}......37
Figure S43. ^{13}C NMR spectrum (176 MHz, CD_3OD) of HTM_{222}......37
Figure S44. ^{13}C-DEPT-135 NMR spectrum (176 MHz, CD_3OD) of HTM_{222}......38
Figure S45. ^{1}H-^{1}H COSY spectrum (700 MHz, CD_3OD) of HTM_{222}......38
Figure S46. HSQC spectrum (CD_3OD) of HTM_{222}......39
Figure S47. HMBC spectrum (CD_3OD) of HTM_{222}......39
Figure S48. NOESY spectrum (CD_3OD) of HTM_{222}......40
Figure S49. Identification of HTM_{222} by LC-ESI-HRMS......41
Table S4. Deduced functions of ORFs in putative HTM biosynthetic gene cluster......42
Figure S50. Deletion of the entire HTM biosynthetic gene cluster and its verification......44
Figure S51. Scheme presentation of *htmA7* deletion and verification......44
Figure S52. Site-directed mutation of DH_1 in module 1 of HtmA1......45
Figure S53. Site-directed mutation of KR_1 in module 1 of HtmA1......45
Figure S54. The partial sequence alignment of KS in HTM PKS......46
Figure S55. Site-directed mutation of KR_2 in module 2 of HtmA1......46
Figure S56. Site-directed mutation of DH_3 in module 3 of HtmA1......47
Figure S57. Site-directed mutation of ACP_2 in module 2 of HtmA1......47
Figure S58. Site-directed mutation of ACP_3 in module 3 of HtmA1......48
Figure S59. Site-directed mutation of KS_3 in module 3 of HtmA1......48
Figure S60. Proposed mechanism of the dehydrating bimodule (modules 2 and 3) of HtmA1......49
Figure S61. SDS-PAGE analysis of all recombinant enzymes used in this study......50
Figure S62. ^{1}H NMR spectrum of compound **2** (400 MHz, $(CD_3)_2SO$) 51
Figure S63. ^{13}C NMR spectrum of compound **2** (150 MHz, $(CD_3)_2SO$) 52
Figure S64. ESI-HRMS of compound **2**......53
Scheme S2. Preparation of a) (*rac*)-**3** and b) enantiomerically enriched (*R*)-**3** by kinetic resolution through Sharpless epoxidation of ethyl (*rac*)-3-hydroxyhex-4-enoate (**S13**) and saponification......54
Figure S65. ^{1}H NMR spectrum of compound **3** (400 MHz, CD_3OD) 56
Figure S66. ^{13}C NMR spectrum of compound **3** (100 MHz, CD_3OD) 56
Figure S67. ESI-HRMS of compound **3**......57
Figure S68. Confirmation of configuration of C3-hydroxy group in the product **3** from in vitro assay of *holo*-module 2 with synthetic **2** by chiral HPLC analysis......57
Scheme S3. Preparation of (*S*)-**3** and (*R*)-**3** by Evans' acyl oxazolidinone......58
Figure S69. ^{1}H NMR spectrum of compound **S14** (500 MHz, $CDCl_3$) 60
Figure S70. ^{13}C NMR spectrum of compound **S14** (125 MHz, $CDCl_3$) 60
Figure S71. ^{1}H NMR spectrum of compound (*S*)-**S15** (500 MHz, C_6D_6) 61
Figure S72. ^{13}C NMR spectrum of compound (*S*)-**S15** (125 MHz, C_6D_6) 61
Figure S73. ^{1}H NMR spectrum of compound (*R*)-**S15** (500 MHz, C_6D_6) 62
Figure S74. ^{13}C NMR spectrum of compound (*R*)-**S15** (125 MHz, C_6D_6) 62
Figure S75. ^{1}H NMR spectrum of compound **3** (500 MHz, C_6D_6) 63
Figure S76. ^{13}C NMR spectrum of compound **3** (125 MHz, C_6D_6) 63
Figure S77. ^{1}H NMR spectrum of compound **S16** (500 MHz, $CDCl_3$) 64
Figure S78. ^{13}C NMR spectrum of compound **S16** (125 MHz, $CDCl_3$) 64
References 65
Author Contributions......65

Bacterial strains, plasmids and DNA manipulation. Bacterial strains and plasmids of this study are summarized in Table S1. DNA manipulations were performed using standard procedures for *E. coli* and *Streptomyces*. The chemical reagents and antibiotics were purchased from Sigma-Aldrich. The test kits for alanine aminotransferase (ALT) and aspartate aminotransferase (AST) activity were purchased from Nanjing Jiancheng Bioengineering Institute. Oligonucleotide primers used in this study (Table S2) were synthesized by Tsingke. DNA sequencing of PCR products was recorded by Tsingke.

Table S1. Bacterial strains and plasmids used in this study.

Strain/Plasmid	Characteristic	Reference
Escherichia coli		
DH10B	Host for general cloning	Invitrogen
ET12567/pUZ8002	Donor strain for conjugation between *E. coli* and *Streptomyces*	[1]
BL21 (DE3)	Host for protein expression	Invitrogen
BAP1	Host for protein expression	[2]
pGro7	Host for protein expression	Takara
Streptomyces spectabilis		
CCTCC M2017417	Hangtaimycin (HTM) producing wild-type strain	[3]
∆htm	HTM biosynthetic gene cluster deletion mutant strain	Figure S50
∆htmA7	*htmA7* in-frame deletion mutant strain	Figure S51
DH_1(H26A)	Site-directed mutation strain of DH_1 in module 1 of HtmA1	Figure S52
KR_1(G10A, G12A, G15A)	Site-directed mutation strain of KR_1 in module 1 of HtmA1	Figure S53
KR_2(Y168A)	Site-directed mutation strain of KR_2 in module 2 of HtmA1	Figure S55
DH_3(H25A)	Site-directed mutation strain of DH_3 in module 3 of HtmA1	Figure S56
ACP_2(S40A)	Site-directed mutation strain of ACP_2 in module 2 of HtmA1	Figure S57
ACP_3(D43L, S44A)	Site-directed mutation strain of ACP_3 in module 3 of HtmA1	Figure S58
∆KS_3(C155A)	Site-directed mutation strain of KS_3 in module 3 of HtmA1	Figure S59
**Plasmid**		
pYH7	*Streptomyces-E. coil* shuttle vector	[4]
pET28a(+)	Vector for protein expression	Invitrogen
pWHU5001	Recombinant plasmid used for HTM biosynthetic gene cluster deletion in vivo	Figure S50
pWHU5002	Recombinant plasmid used for *htmA7* in vivo in-frame deletion	Figure S51
pWHU5003	Recombinant plasmid used for in vivo site-directed mutation of DH_1 in module 1 of HtmA1	Figure S52
pWHU5004	Recombinant plasmid used for in vivo site-directed mutation of KR_1 in module 1 of HtmA1	Figure S53
pWHU5005	Recombinant plasmid used for in vivo site-directed mutation of KR_2 in module 2 of HtmA1	Figure S55
pWHU5006	Recombinant plasmid used for in vivo site-directed mutation of DH_3 in module 3 of HtmA1	Figure S56
pWHU5007	Recombinant plasmid used for in vivo site-directed mutation of ACP_2 in module 2 of HtmA1	Figure S57
pWHU5008	Recombinant plasmid used for in vivo site-directed mutation of ACP_3 in module 3 of HtmA1	Figure S58
pWHU5009	Recombinant plasmid used for in vivo site-directed mutation of KS_3 in module 3 of HtmA1	Figure S59
pWHU5011	Recombinant plasmid used for module 1 protein expression in *E. coli*	This work
pWHU5012	Recombinant plasmid used for module 2 protein expression in *E. coli*	This work
pWHU5013	Recombinant plasmid used for module 3 protein expression in *E. coli*	This work
pWHU5014	Recombinant plasmid used for module 3 KS_3(C155A) protein expression in *E. coli*	Figure S59
pWHU5015	Recombinant plasmid used for HtmA7 (*trans*-ATs) protein expression in *E. coli*	This work

Table S2. List of oligonucleotide primers used in this study.

Primer	Oligonucleotide sequence (5' to 3')	Restriction site
htm-L-up	TCAAGGCGAATACTT**CATATG**GCGCGGCTGATCGGCCGACG	*Nde*I
htm-L-re	GTGCGGGCCCCCGCCGAAAGGCCCACGCGC	
htm-R-up	CTTTCGGCGGGGGCCCGCACCCCAGAGGCA	
htm-R-re	GAC**CTGCAG**GCATGC**AAGCTT**TCTCCTCGTCCGACGTCGTC	*Pst*I/*Hind*III
htmA7-L-up	AAGGCGAATACTT**CATATG**TCGTGCTGCCCTTCCTGAT	*Nde*I
htmA7-L-re	TTGAGGAAGTTCAGCGAGTAGCCGAGCAGCC	
htmA7-R-up	CTACTCGCTGAACTTCCTCAAGCACGGCTA	
htmA7-R-re	TGCAGGCATGC**AAGCTT**AAGTACACCGCGCAGGCCCT	*Hind*III
KR$_1$-L-up	AGGCGAATACTT**CATATG**CCTCGATGGTCGTCCTGT	*Nde*I
KR$_1$-L-re	GGCGAGGCCCAGCGCCTGGCCGGTGATGAGGTACACGC	
KR$_1$-R-up	GCCAGGCGCTGGGCCTCGCCCGGATCTTCGCCAGGGACATCG	
KR$_1$-R-re	CTGCAGGCATGC**AAGCTT**CGTGTAGCCGTTGGTGCG	*Hind*III
DH$_1$(H26A)-L-up	AGGCGAATACTT**CATATG**CCATGCGCCGGACGCCCGGC	*Nde*I
DH$_1$(H26A)-L-re	CCGCCA**CCCGAG**CGTCGCGCAGGAAGAAG	*Ava*I
DH$_1$(H26A)-R-up	GCGCGACG**CTCGGG**TGGCGGGCGAGC	*Ava*I
DH$_1$(H26A)-R-re	CTGCAGGCATGC**AAGCTT**GTGGCGCGGTGGCGTGCGAAC	*Hind*III
KR$_2$(Y168A)-L-up	CAAGGCGAATACTT**CATATG**CGCTGCACTCCGCGACGCTC	*Nde*I
KR$_2$(Y168A)-L-re	TGCCCATAGCGGCGTCGCTCTGTCCGGCGCCG	
KR$_2$(Y168A)-R-up	GCGACGCCGCTATGGGCAACGCCTTCATGGAC	
KR$_2$(Y168A)-R-re	ACCTGCAGGCATGC**AAGCTT**CGTCGACGCGCCGCGTCCAC	*Hind*III
DH$_3$(H25A)-L-up	AGGCGAATACTT**CATATG**CGTCACCAGCGTCCAGTGG	*Nde*I
DH$_3$(H25A)-L-re	CGTAG**ACGCGT**GCTCCGTCCACGACGGC	*Mlu*I/*Afl*III
DH$_3$(H25A)-R-up	GGACGGAGC**ACGCGT**CTACGGACGGCGGCT	*Mlu*I/*Afl*III
DH$_3$(H25A)-R-re	CTGCAGGCATGC**AAGCTT**GCGTACACGGCGTCACCG	*Hind*III
ACP$_2$(S40A)-L-up	CAAGGCGAATACTT**CATATG**GACACCGCCGTCGACGGCGT	*Nde*I
ACP$_2$(S40A)-L-re	GAGCAGGAACGCGTCGGCGCCGTAGTCGGCG	
ACP$_2$(S40A)-R-up	GCCGACGCGTTCCTGCTCGCGCAGGTCCTC	
ACP$_2$(S40A)-R-re	ACCTGCAGGCATGC**AAGCTT**CGAGTGCACGAGCTCCTGCG	*Hind*III
KS$_3$(C155A)-L-up	CAAGGCGAATACTT**CATATG**GCGAAGGCGTCGCGGGACGT	*Nde*I
KS$_3$(C155A)-L-re	TGAGCGCGGAGCTAGCGGCGGTGTCCATGACCATGG	
KS$_3$(C155A)-R-up	TGGACACCGCCGCTAGCTCCGCGCTCACGGCGATGAA	
KS$_3$(C155A)-R-re	ACCTGCAGGCATGC**AAGCTT**CCGGTGCCGCAGGCTCGTG	*Hind*III
ACP$_3$-L-up	CAAGGCGAATACTT**CATATG**GGCTCGCGCACCGGCGTGTA	*Nde*I
ACP$_3$-L-re	CAGCGTCGCCGCTAGCAGGCCGAGCGCGTAGT	
ACP$_3$-R-up	GCCTGCTAGCGGCGACGCTGCTGCGGATCGC	

ACP_3-R-re	ACCTGCAGGCATGCAAGCTTGGTACGTGGGCAGGTGCGCG	*Hin*dIII
htm-confirm-up	TCGTCATCGGTACGGAAGAGG	
htm-confirm-re	GCGGAGTTCTGGTTCGAGATG	
htmA7-confirm-up	CCCAAGCAGAAGATGGCCCACG	
htmA7-confirm-re	TGACCGGCCTGATCCGAGTG	
KR1-comfirm-up	TCCTCCAGGTCCTCACCCACG	
KR1-comfirm-re	CCCATGACGGACGAGAACAGC	
DH_1(H26A)-confirm-up	CGGTGTCGACTGGGAGGC	
DH_1(H26A)-confirm-re	CGCAGCGTGGCGTAGAAGT	
KR_2(Y168A)-confirm-up	CTGTGCGCCCAGCACTTCGT	
KR_2(Y168A)-confirm-re	CCTCGTCCAACTGGCCTGACTT	
DH_3(H25A)-confirm-up	GAGGGCATCGCCGCCTTCAT	
DH_3(H25A)-confirm-re	CACCGAGCCGACGACCTTCAT	
ACP_2(S40A)-confirm-up	TCGGCGAGGTCACGAACAAGA	
ACP_2(S40A)-confirm-re	TCGGCGTGCAGCATGAAGTG	
KS_3(C155A)-confirm-up	CCTGGCTGCTCGACACCTATCC	
KS_3(C155A)-confirm-re	CCTCCCGCATCACCTGCTTCT	
ACP_3-comfire-up	GTTCGTTCCACCCGGCATTG	
ACP_3-comfire-re	GCGCCCGGACTTGAGGTTCT	
HtmM1-up	TGCCGCGCGGCAGC**CATATG**CTCAGGGGCGAGGAAACAAGCCA	*Nde*I
HtmM1-re	CGGGCCCGCGGGCCACGTGGCCCGGTGGCCGCCGAGGA	
HtmM2-up	GTGCCGCGCGGCAGC**CATATG**TCCGCCCTGGACATCGCC	*Nde*I
HtmM2-re	TGTCGACGGAGCTCGAATTCTCACTCGGCGGGATAGGTGTCG	
HtmM3-up	GTGCCGCGCGGCAGC**CATATG**CACAGGGTCGCCGTGGTCGGC	*Nde*I
HtmM3-re	TGTCGACGGAGCTCGAATTCTCACGCAGGCTCGTGGTGGCGG	

Nucleotides in bold type are restriction sites introduced.

Culture and fermentation conditions. For hangtaimycin (HTM, **1**) production, the wild-type of S*treptomyces spectabilis* CCTCC M2017417, which was maintained on ABB13 plates (0.5% soytone, 0.5% soluble starch, 0.3% $CaCO_3$, 0.2% MOPS, 2% agar), was grown in TSBY liquid medium (3% tryptone soy broth, 10.3% sucrose, 0.5% yeast extract) at 28 °C with shaking of 220 rpm for 20 h as seed culture. It was then inoculated to the SFMR medium (2% soya flour, 2% mannitol, 5% AB-8 macroporous adsorption resin) and incubated for another 4 days on the fermentation condition of 28 °C and 220 rpm. *E. coli* strains were cultured in 2×TY (1.6% tryptone, 1% yeast extract, 0.5% NaCl) or on 2×TY agar medium at 37 °C with the appropriate antibiotic at a concentration of 100 μg/mL ampicillin, 35 μg/mL apramycin, 50 μg/mL kanamycin, 25 μg/mL chloramphenicol, or 30 μg/mL nalidixic acid for selection.

LC-ESI-HRMS analysis of the extracts of wild-type and mutant strains. After fermentation in SFMR medium for 4 days, the cultures of wild-type and mutant strains were extracted with equal ethyl acetate. The extracts were obtained after the ethyl acetate evaporated under reduced pressure. Subsequently, the extracts were dissolved in 1.5 mL CH_3CN and analyzed, respectively, by LC-ESI-HRMS on a Thermo Electron LTQ-Orbitrap XL mass spectrometer equipment with a Phenomenex Luna C18 column (250×4.6 mm) eluting with a linear gradient elution system of CH_3CN/H_2O (0-20 min, 35-65% B; 20-28 min, 65-95% B; 28-30 min, 95% B; 30-33 min, 95-35% B; 33-38 min, 35% B) at a flow rate of 1 mL/min. LC-ESI-HRMS analyses were carried out in the positive ionization mode with 35% relative collision energy.

Isolation of HTM from *S. spectabilis* CCTCC M2017417. After fermentation in modified SFMR medium (8 L) for 4 days at 28 °C and 220 rpm, the fermentation extracts were filtered with 40 mesh sieve to separate the broth, mycelium and resin. The supernatant was then extracted with an equal volume of ethyl acetate. The mycelium and the resin were also extracted with ethyl acetate. After evaporation to dryness under reduced pressure, the residues obtained from the culture supernatant, the mycelium and the resin were combined and subjected to silica gel CC to obtain eight fractions (Fr.A1−Fr.A8) eluting with a mixture of CH_2Cl_2/CH_3CN (100:0, 98:2, 96:4, 92:8, 90:10, 80:20, 70:30, 50:50, v/v). Fr.A7+Fr.A8 were combined and subjected to MPLC with an ODS column eluting with CH_3CN/H_2O (0−20 min, 0:100−40:60; 20−45 min, 40:60−80:20; 45−60 min, 80:20−100:0; v/v) at a flow rate of 15 mL/min to get Fr.B1−B16. Fr.B8−B9 were combined and further purified with preparative HPLC eluting with a mixture of CH_3CN/H_2O (0−20 min, 45:55−55:45) at a flow rate of 3 mL/min to yield HTM at 17.5 min with a yield of 15.5 mg. Finally, the purified HTM were recorded of its NMR and LC-ESI-HRMS spectra for structure elucidation.

Hepatoprotective effect in vitro. Human hepatoma cell line HepG2 (Wuhan Hua Lian Biotechnology Co., Ltd) were seeded into 24-well plates and cultured in Dulbecco's Modified Eagle Medium with High Glucose (DMEM-HG, TBD10569) supplemented with 10% fetal bovine serum (FBS, Hangzhou Sijiqing Bio-logical Engineering Materials Co., Ltd.), and incubated under 5% CO_2 at 37 °C. In vitro liver injury model was built based on literatures with some modification.[5,6] After 24 hours incubation, the supernatant was replaced with culture medium containing CCl_4 (70%) and incubated for 4 h except the control group. To identify the hepatoprotective effect of HTM, cells were then treated with HTM for 4 h. Silymarin was used as positive control for the liver protective effects. The activities of ALT and AST were measured by reading the absorbance at 510 nm with Microplate Spectrophotometer (Bioteck EON). All results are presented as the mean ± standard error of mean (SEM). Statistical analyses of the data with equal variances were carried out by one-way or two-way analysis of variance (ANOVA), followed by Tukey’s post-hoc test where appropriate. $P<0.001$ was considered most significant, $P<0.01$ very significant, $P<0.05$ significant and $P>0.05$ indicated no statistical difference.

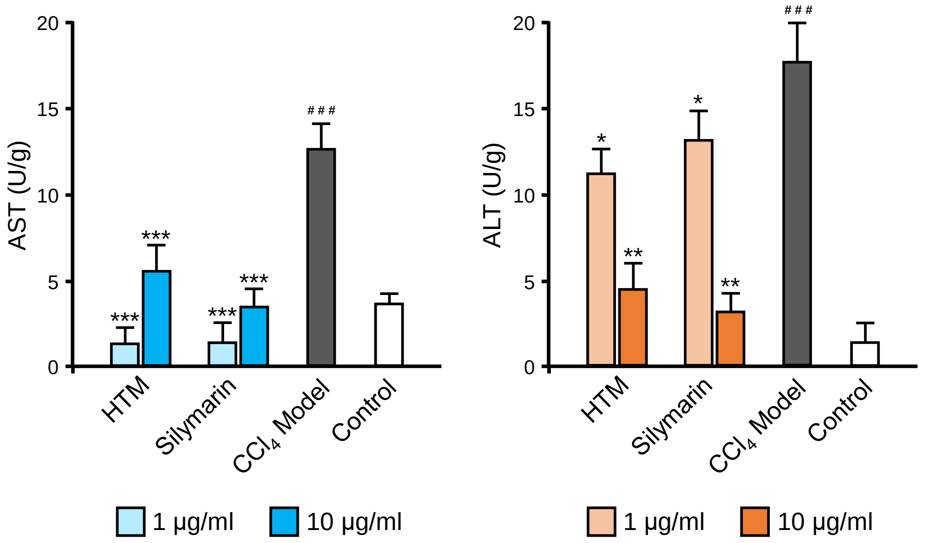

Figure S1. Hepatoprotective effects of HTM (**1**) against damage induced by CCl_4 in HepG2 cells. After treated with HTM and silymarin (positive control) for 4 hours, the activity of ALT (A) and AST (B) of HepG2 cells were measured using assay kits. The values are the mean ± SD. ###, $P<0.001$ vs control; *, $P<0.05$ vs CCl_4; **, $P<0.01$ vs CCl_4; ***, $P<0.001$ vs CCl_4, n=3.

NMR analysis of HTM and synthetic compounds. The 1D and 2D NMR spectra of the purified HTM, synthesis substrates and standards were performed on an Agilent 400/600MR DD2 NMR spectrometer. Chemical shifts (δ) are given in ppm with reference to TMS. NMR data processing was obtained by MestReNova software.

Structure elucidation of HTM. HTM was obtained as white power. It had a molecular formula of $C_{50}H_{61}N_7O_{11}$ deduced from its protonated ion at *m/z* 936.4523 ($[M+H]^+$) observed in its the LC-ESI-HRMS (calcd. for $C_{50}H_{62}N_7O_{11}$, 936.4502). The ^{1}H-NMR spectrum of HTM unveiled signals for 18 olefinic proton signals, 5 amino/amide proton signals, seven methyl groups (including one oxygenated methyl group at δ_H 3.18 and one nitrogenated methyl group at δ_H 2.94), five methylene and six methine proton signals. The ^{13}C-NMR and HSQC spectrum of HTM further assigned the signals unveiled by ^{1}H-NMR spectrum, and also revealed 15 quaternary carbons, including 8 amide or ester carbonyls, together with 7 sp2 carbons. The ^{1}H-^{1}H COSY correlations of NH-1/H-2, H-4/H-5/H-6/H-7, together with the HMBC correlations of NH-1/C-3, C-9; H-2/C-3, C-8 and C-9; H-4/C-3, C-6, C-8, C-9; H-5/C-4, C-6, C-7, C-9; H-7/C-5, C-6, C-8, C-9 indicate presence of a C-3-substituted indolyl moiety. The chemical shift of the two amide carbonyls at δ_C 162.3 (C-13), 172.1 (C-16), together with the HMBC correlation of H3-18/C-17, C-14; H-17/C-13, C-14, C-18; H-11/C-3, C-10, C-13, C-16; N-CH3/C-11, C-13 suggested the presence of a cyclodipeptide (*N*-methyltryptophan-dehydrothreonine) moiety. The left signals were assigned as five fragments of C-20/C-21(21-OCH_3)/C-22; C-25/C-26; C-29/C-30/C-31/C-32/C-33/C-34/C-35; C-37/C-38/C-42; C-45/C-46/C-47/C-48/C-49; C-52/C-53/C-54/C-55/C-56 according to the COSY and HMBC correlations of HTM. The HMBC correlations of H-20/C-19, C-24; NH-23/C-19, C-20, C-24; NH-27/C-24, C-25, C-28, C-29; H-29/C-28; H-35/C-36, C-37, C-40, C-41; H-38/C-40, C-36; H-42/C-44; H-46/C-44, C-47; H-47/C-44, C-48, C-49; H-48/C-47, C-51; NH-50/C-48, C-51, C52 linked these fragments as a long fragment of C19-C56. This long fragment was further connected to the cyclodipeptide moiety via C-19, which was suggested by its chemical shift of δ_C 167.0 (C-19). The NMR data of HTM were nearly the same with that of HTM reported previously. [7] By careful comparison and analysis, we confirmed that HTM was HTM and revised the chemical structure of HTM of the published structure with respect to the $\Delta^{29,30}$ double bond configuration according to the NMR data. As showed in the NMR datasets (Table S3), there is no ^{1}H-^{1}H ROESY's correlations between H_{29} and H_{30}, and the coupling constants for H_{30} (dd, 15.0, 10.2 Hz) also indicate an *E* configuration for the $\Delta^{29,30}$ double bond. Although the signals for H_{29} were unfortunately overlapped, but the 15.0 Hz coupling constant could only be for the coupling constants of $^3J_{H\text{-}29/H\text{-}30}$. The other coupling constant of 10.2 Hz must be for the coupling constants of $^3J_{H\text{-}30/H\text{-}31}$ (this coupling is not over a double bond, so this should not be 15.0 Hz). For this reason, the $\Delta^{29,30}$ double bond configuration was revised as *E* configuration. Thus the structure of HTM was determined as depicted in Figure 1.

Table S3. ^{13}C (150 MHz) and ^{1}H (600 MHz) NMR data for HTM (**1**).

Position	δ_H multi.(*J* in Hz)	δ_C	HMBC	^{1}H-^{1}H COSY
1	10.94, brs		C-2,3,8,9	H-2
2	6.94, d (2.4)	125.7	C-8,9	H-1
3		107.3		
4	7.32, d (8.4)	118.0	C-6,8	H-5
5	6.92, t (7.2)	118.7	C-7,9	H-4
6	7.02, t (7.2)	121.1	C-4,8	H-7
7	7.29, d (8.4)	111.2	C-5,9	H-6
8		136.2		
9		127.0		
10	3.24, dd (14.4, 4.8)；3.34 dd (14.4, 4.2)	26.4	C-11,3,16,9	H-11
11	4.7, dd (4.8, 4.2)	64.7	C-3,10,13,16	H-10
12N-CH_3	2.94, s	31.4	C-11,13	
13		162.3		
14		125.3		
16		172.1		
17	5.52, q (7.2)	129.6	C-13,14,18	H-18
18	0.60, d (7.2)	13.4	C-14,17	H-17
19		167.0		
20	5.72, dd (8.4, 3.6)	55.8	C-19,21	H-21,23
21	3.66, m	75.9	C-21-OCH_3	H-20,22
21-OCH_3	3.18, s	56.4	C-21,22	
22	0.98, d (6.0)	15.5	C-21,20	H-21
23	8.02, d (8.4)		C-20,21	H-20
24		164.2		
25		135.8		
26	5.49, s	105.2	C-24,25	
27	9.28, s		C-28	
28		164.7		
29	6.27, d (15.0)	123.1	C-28,31	H-30
30	7.09, dd (15.0, 10.2)	141.0	C-28,29,31,32	H-29
31	6.22, m (overlap)	128.9	C-29,30,33	H-29,33
32	6.19, dt (15.0, 6.6)	142.3	C-30,31,33,34	H-33
33	2.17, q (7.2)	31.7	C-31,32,34,35	H-32,34
34	1.62, m	25.1	C-32,33,35	H-33,35
35	2.29, t (7.2)	35.4	C-33,34,37, 41	H-34,41
36		163.7		
37	2.47, m (overlap)	28.6	C-35,38, 42, 41	H-38,41
38	4.37, m	77.2	C-36,37,40,42	H-37,42
40		161.6		
41	5.72, s	114.5		
42	5.36, dd (13.2, 7.2)	73.4	C-37,38, 44	H-38
43	8.22, d (8.4)		C-44	
44		168.3		
45		130.5		
46	1.83, s	12.8	C-44,45,47	H-47
47	6.13, d (9.0)	137.4	C-46,44,45,49	H-46,48
48	4.64, m	42.4	C-45,47,49,51	H-47,49,50
49	1.16, d (6.6)	20.3	C-47,48	H-48
50	8.14, d (7.2)		C-49,51,52	H-48
51		164.6		
52	5.57, d (11.4)	119.1	C-51,53,54	H-53
53	6.35, t (11.4)	140.1	C-51,54,55	H-52,54
54	7.49, dd (14.4, 12.0)	128.6	C-53,56	H-53,55
55	5.94, m	136.9	C-53,56	H-54,56
56	1.78,d (6.6)	18.3	C-54,55	H-55

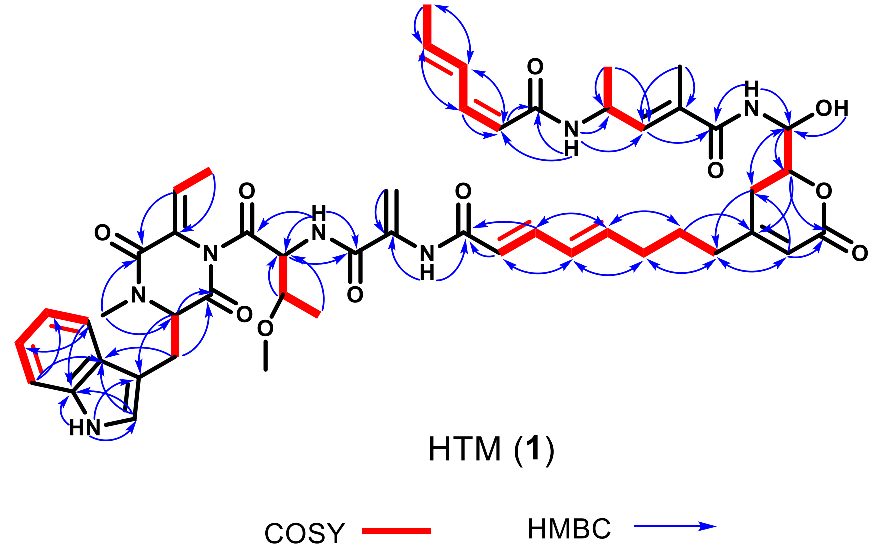

Figure S2. Key COSY and HMBC correlation of HTM (**1**).

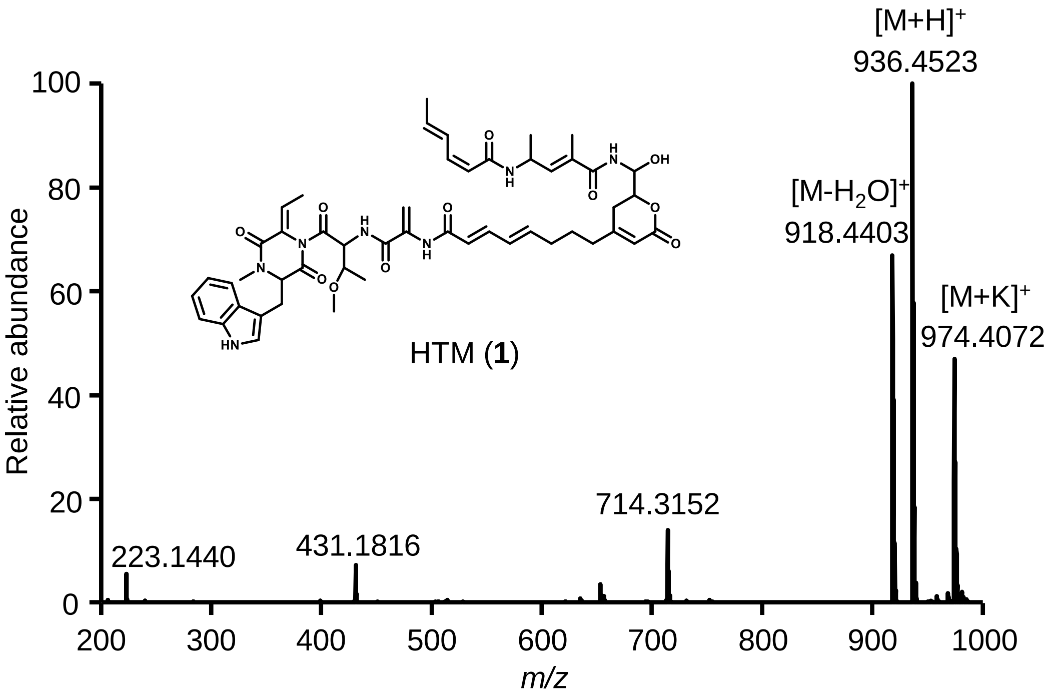

Figure S3. ESI-HRMS spectrum of HTM (**1**).

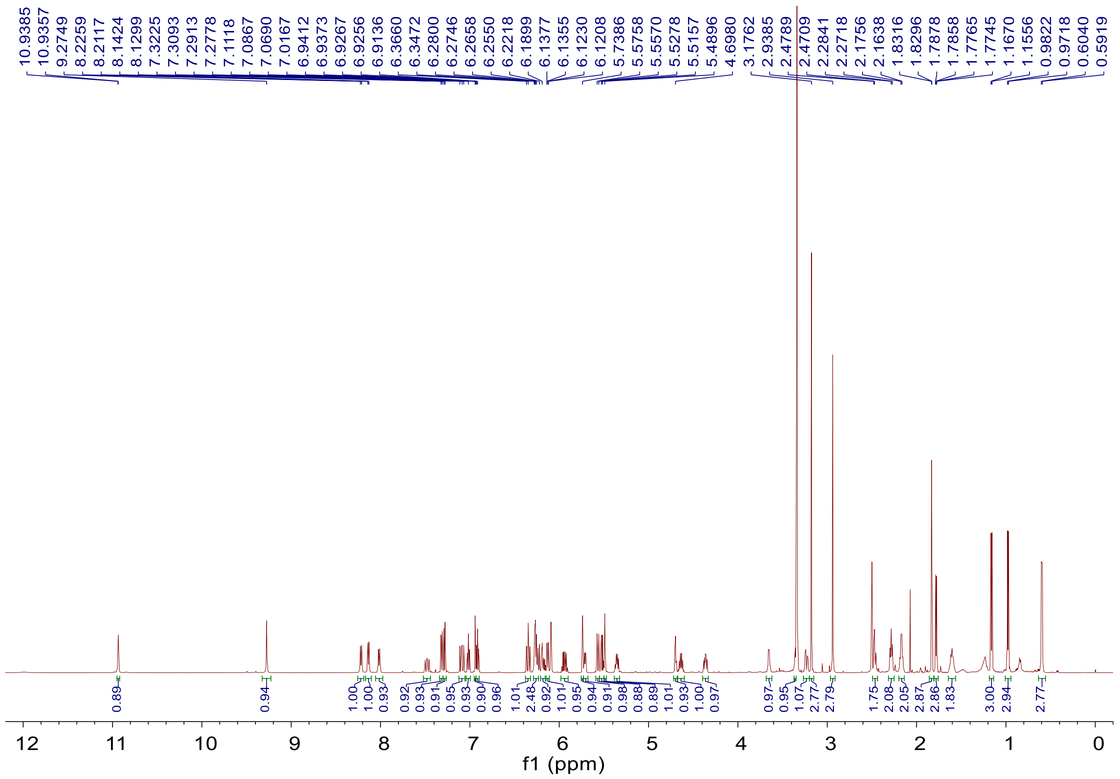

Figure S4. ^1H NMR spectrum of HTM (**1**) (600 MHz, $(CD_3)_2SO$).

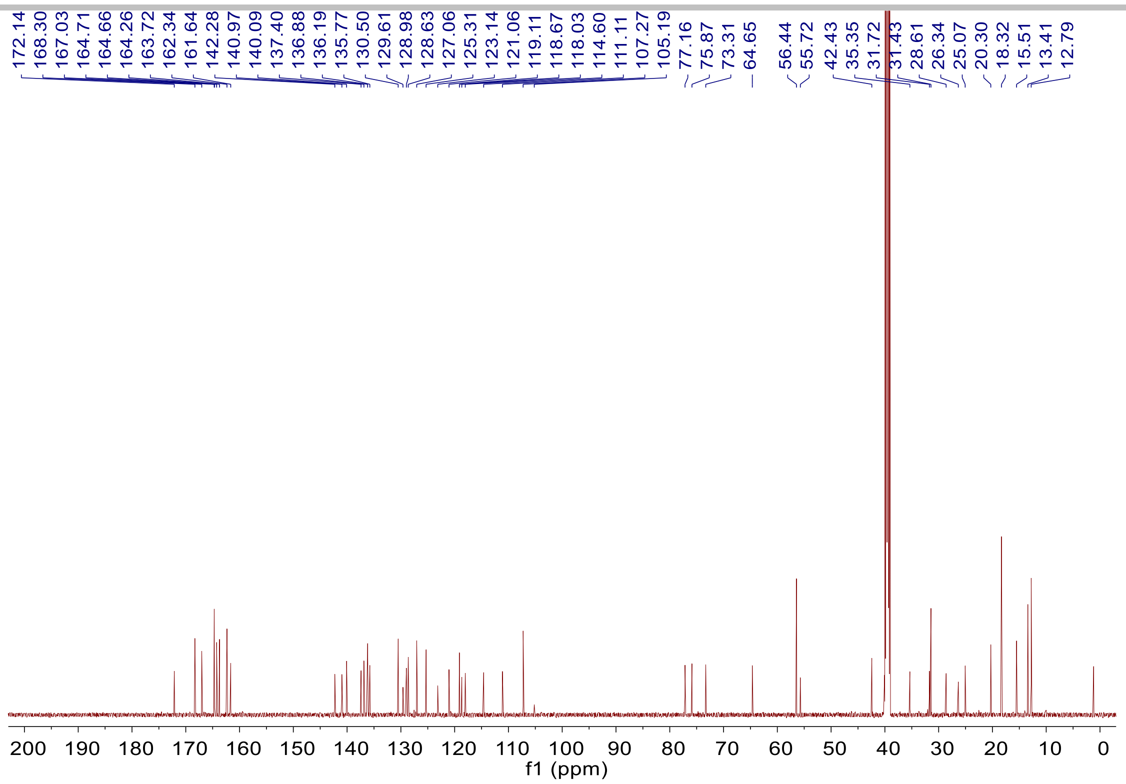

Figure S5. ^{13}C NMR spectrum of HTM (**1**) (150 MHz, $(CD_3)_2SO$).

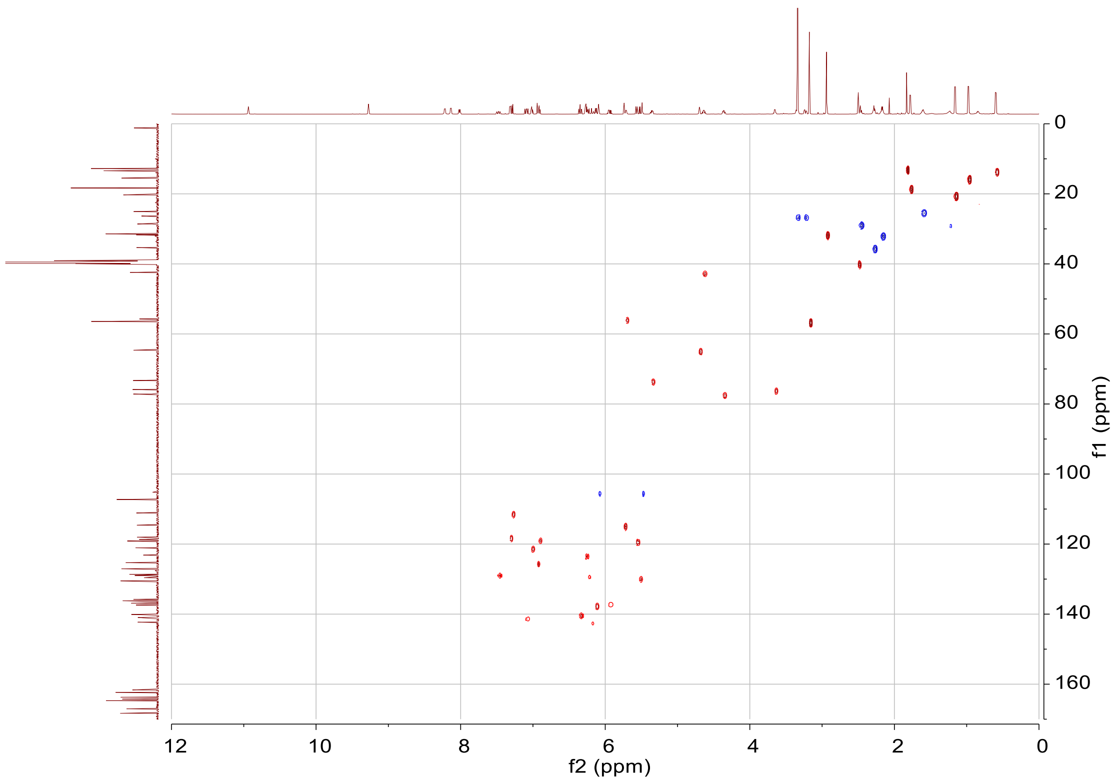

Figure S6. HSQC spectrum of HTM (**1**) (600 MHz, $(CD_3)_2SO$).

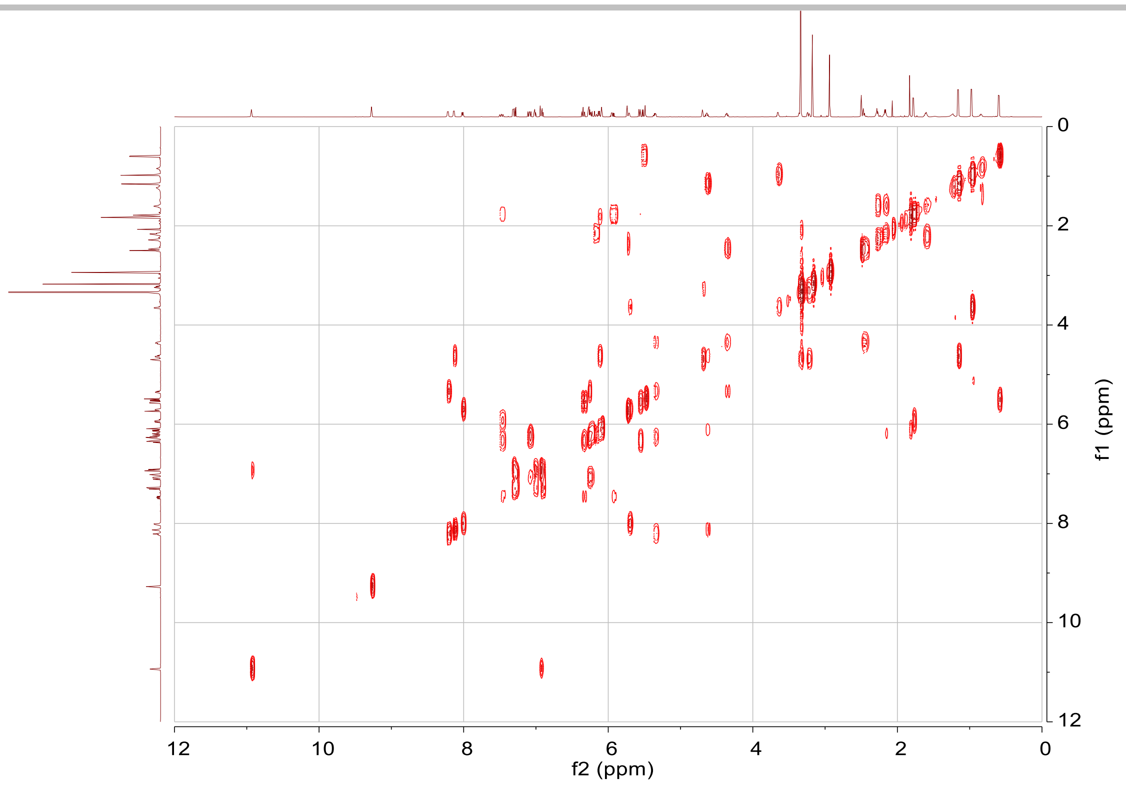

Figure S7. ^{1}H-^{1}H COSY spectrum of HTM (**1**) (600 MHz, $(CD_3)_2SO$).

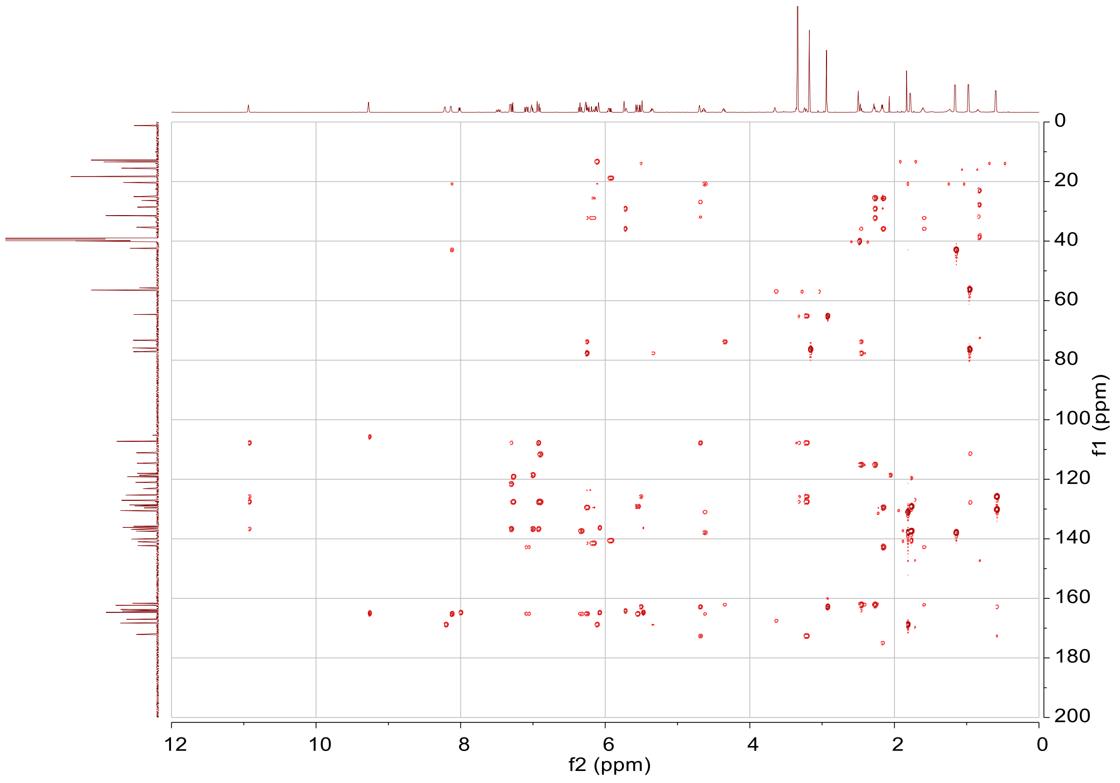

Figure S8. HMBC spectrum of HTM (**1**) (600 MHz, $(CD_3)_2SO$).

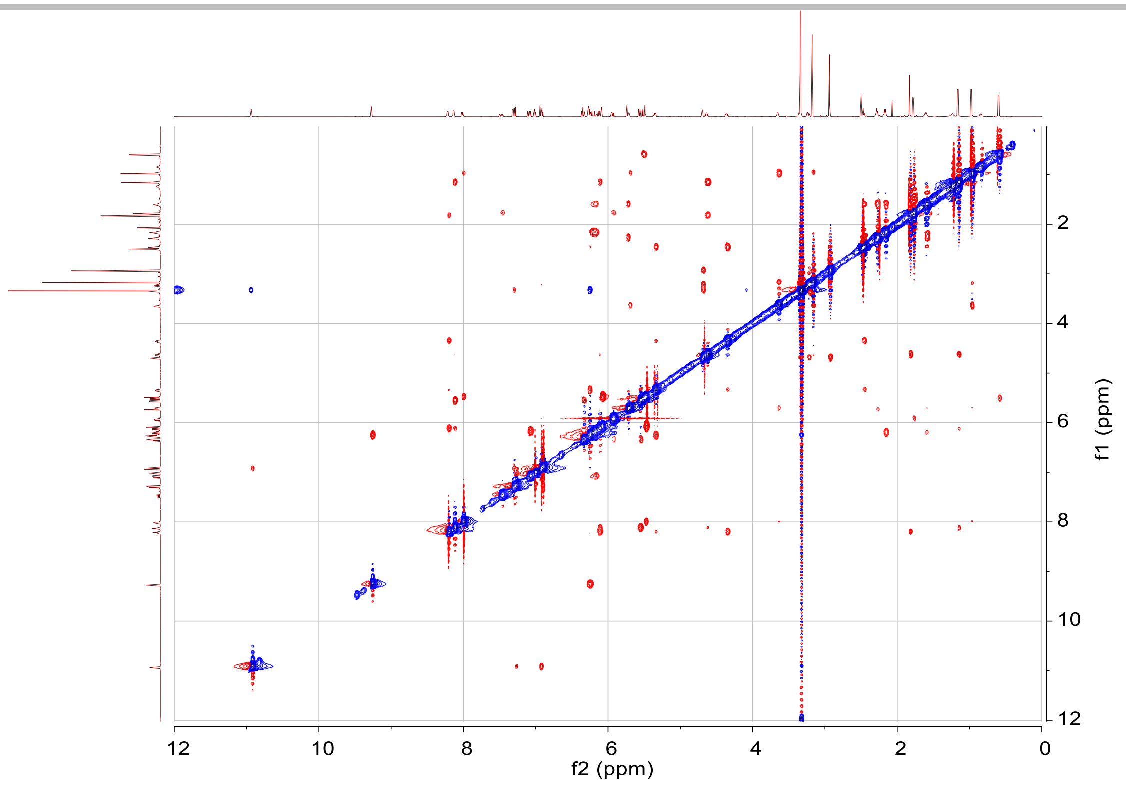

Figure S9. ROESY spectrum of HTM (**1**) (600 MHz, $(CD_3)_2SO$).

a)

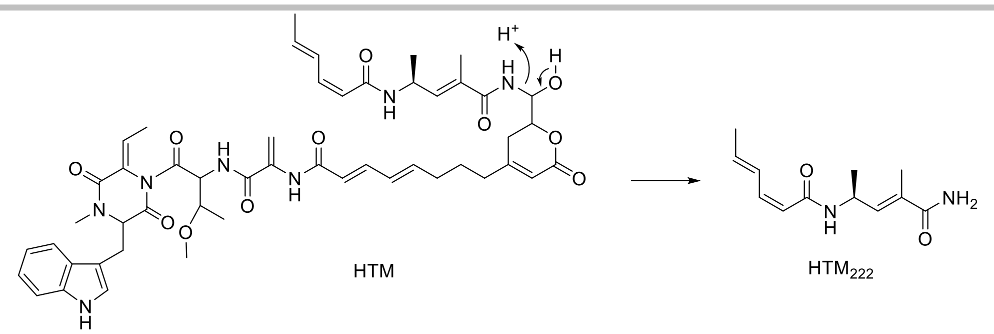

b)

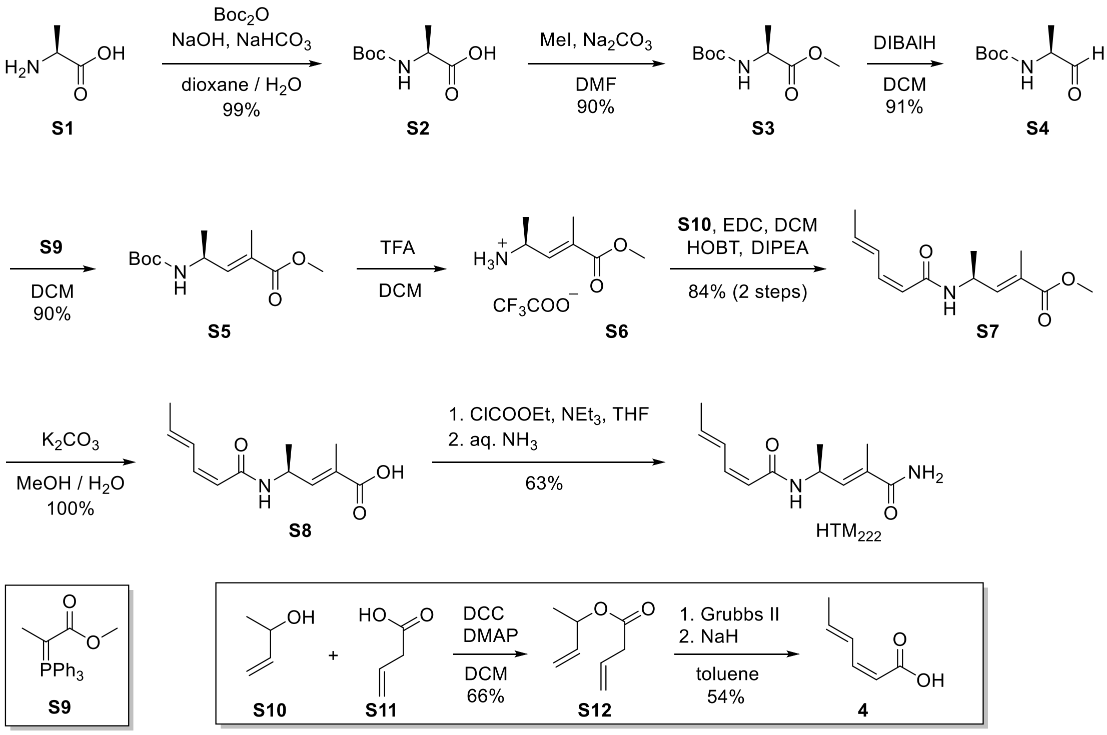

Scheme S1. a) Putative degradation of hangtaimycin to sarmentosamide (HTM_{222}). b) Synthesis of sarmentosamide.

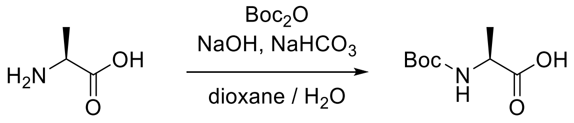

S1 **S2**

To a solution of dioxane / water (2 : 1, 150 mL) was added L-alanine **S1** (4.45 g, 50.0 mmol), followed by NaOH (aq., 1 M, 50 mL). The reaction mixture was cooled to 0 °C. $(Boc)_2O$ (16.37 g, 75.0 mmol) and $NaHCO_3$ (4.20 g, 50.0 mmol) were then added. After being stirred at room temperature for 18 h, the reaction solvent was evaporated to half of the original volume. The residue was diluted with EtOAc (200 mL), followed by cooling to 0 °C and acidification to pH = 2 with HCl (aq., 1 mM). The layers were separated and the aqueous phase was extracted with EtOAc (2 × 100 mL). The combined organic phase was washed with brine, dried over $MgSO_4$ and then concentrated under reduced pressure to give **S2** as white solid.

(*tert*-Butoxycarbonyl)-L-alanine (S2). Yield: 9.41 g, 49.8 mmol (99%); **Optical rotation**: $[\alpha]_D^{25}$ = –21.7 (*c* 0.23, MeOH). **HRMS** (ESI): calculated for $C_8H_{15}NO_4Na^+$ 212.0893, found 212.0897; **IR** (diamond ATR): ṽ / cm^{-1} = 2979 (m), 2933 (w), 1694 (s), 1511 (m), 1453 (w), 1367 (m), 1158 (s), 1070 (m), 1023 (w), 638 (m); **^{1}H-NMR** (500 MHz, CD_3OD): δ (ppm) 4.11 (q, 1H, J = 7.2 Hz), 1.44 (s, 9H), 1.35 (d, 3H, J = 7.3 Hz); **^{13}C-NMR** (126 MHz, CD_3OD): δ (ppm) 176.8, 157.9, 80.4, 50.4, 28.7, 17.9.

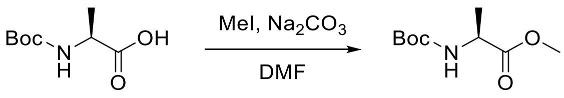

S2 **S3**

Boc-protected glycine **S2** (5.00 g, 26.5 mmol) was added to DMF (40 mL), followed by Na_2CO_3 (6.05 g, 57.1 mmol) and methyl iodide (7.00 mL, 112.4 mmol). The solution was stirred at room temperature for 16 h and then diluted with EtOAc (200 mL). The organic layer was washed with water (40 mL) and brine (40 mL), and dried over $MgSO_4$. After removal of the solvent under reduced pressure, the residue was purified by column chromatography on silica gel (cyclohexane : EtOAc = 2 : 1) to give **S3** as white solid.

Methyl (*tert*-butoxycarbonyl)-L-alaninate (S3). TLC (cyclohexane : EtOAc = 2 : 1): R_f = 0.52; **Yield**: 4.83 g, 23.8 mmol (90%); **Optical rotation**: $[\alpha]_D^{20}$ = –39.3 (*c* 0.20, MeOH); **HRMS** (ESI): calculated for $C_9H_{17}NO_4Na^+$ 226.1050, found 226.1051; **IR** (diamond ATR): ṽ / cm^{-1} = 3365 (m), 2979 (m), 1744 (m), 1714 (s), 1516 (m), 1454 (w), 1367 (w), 1164 (s), 1069 (m), 981 (w); **^{1}H-NMR** (500 MHz, $CDCl_3$): δ (ppm) 5.09 (s, 1H), 4.27 (q, 1H, J = 7.2 Hz), 3.70 (s, 3H), 1.40 (s, 9H), 1.34 (d, 3H, J = 7.2 Hz); **^{13}C-NMR** (126 MHz, $CDCl_3$) δ (ppm) 173.92, 155.18, 79.86, 52.35, 49.22, 28.38, 18.68.

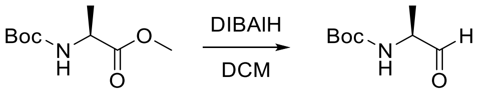

S3 **S4**

A solution of DIBAlH in hexane (1 M, 21.0 mL, 21.0 mmol) was added dropwise to a cooled (–78 °C) solution of **S3** (2.03 g, 10.0 mmol) in CH_2Cl_2 (20 mL). The reaction mixture was stirred at –78 °C for 2 h and then quenched by the addition of saturated potassium sodium tartrate solution (20 mL). The reaction mixture was warmed to room temperature and extracted with EtOAc (3 × 20 mL). The combined organic layers were dried over $MgSO_4$ and concentrated under reduced pressure. The residue was purified by column chromotography on silica gel (cyclohexane : EtOAc = 4 : 1) to give **S4** as white solid.

***tert*-Butyl (*S*)-(1-oxopropan-2-yl)carbamate (S4). TLC** (cyclohexane : EtOAc = 2 : 1): R_f = 0.50; **Yield**: 1.57 g, 9.1 mmol (91%); **Optical rotation**: $[\alpha]_D^{25}$ = –24.7 (*c* 0.22, MeOH); **HRMS** (ESI): calculated for $C_8H_{15}NO_3Na^+$ 196.0944, found 196.0947; **IR** (diamond ATR): ṽ / cm^{-1} = 3347 (m), 2979 (m), 2935 (w), 1687 (s), 1509 (s), 1366 (m), 1247 (m), 1163 (s), 1054 (m), 782 (w); **^{1}H-NMR** (500 MHz, $CDCl_3$): δ (ppm) 9.55 (s, 1H), 5.12 (br, 1H), 4.20 (q, 1H, J =7.4 Hz), 1.44 (s, 9H), 1.32 (d, 3H, J = 7.5 Hz); **^{13}C-NMR** (125 MHz, $CDCl_3$) δ (ppm) 199.9, 155.4, 80.2, 55.6, 28.4, 15.0.

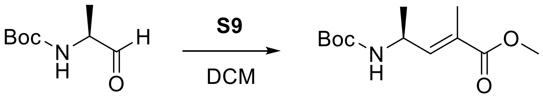

S4 **S5**

S4 (0.69 g, 4.0 mmol) dissolved in CH_2Cl_2 (6 mL) was added to a solution of **S9** (1.73 g, 4.4 mmol, synthesized as reported[8]) in CH_2Cl_2 (8 mL). The solution was stirred at room temperature for 6 h and then concentrated under reduced pressure. The residue was purified by column chromotography on silica gel (cyclohexane : EtOAc = 3 : 1) to give **S5** as white solid.

Methyl (*S*,*E*)-4-((*tert*-butoxycarbonyl)amino)-2-methylpent-2-enoate (S5). TLC (cyclohexane : EtOAc = 1 : 1): R_f = 0.77; **Yield**: 0.87 g, 3.6 mmol (90%); **Optical rotation**: $[\alpha]_D^{25}$ = –9.1 (*c* 0.22, MeOH); **HRMS** (ESI): calculated for $C_{12}H_{21}NO_4Na^+$ 266.1363, found 266.1365; **IR** (diamond ATR): $\tilde{\nu}$ / cm^{-1} = 3359 (m), 2977 (m), 2932 (w), 1690 (s), 1514 (m), 1366 (m), 1246 (s), 1162 (s), 1047 (m), 749 (m); **^{1}H-NMR** (500 MHz, CD_3OD): δ (ppm) 6.58 (m, 1H), 4.47 (br, 1H), 3.77 (s, 3H), 1.93 (s, 3H), 1.46 (s, 3H), 1.22 (d, 3H, *J* = 6.0 Hz); **^{13}C-NMR** (126 MHz, CD_3OD) δ (ppm) 170.0, 157.5, 144.9, 128.4, 80.1, 52.4, 28.7, 20.3, 12.6.

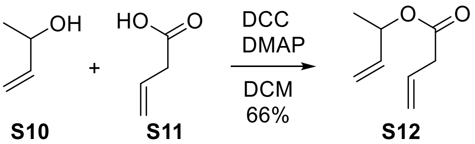

S10 **S11** **S12**

3-Buten-2-ol (**S10**, 3.46 mL, 40 mmol) was dissolved in CH_2Cl_2 (100 mL) and cooled to 0 °C. But-3-enoic acid (**S11**, 3.74 mL, 44 mmol), DCC (9.08 g, 44 mmol) and DMAP (0.49 g, 4 mmol) were added to the cooled solution sequencially. The mixture was stirred at room temperature for 5 h and then filtered, followed by washing with CH_2Cl_2 (3 × 20 mL). Then the combined organic layers were washed with HCl (aq., 1 M, 20 mL) and saturated $NaHCO_3$ solution. After drying over $MgSO_4$ and concentration under reduced pressure, distillation (84 – 85 °C, 100 mbar) was conducted to give **S12** as colorless oil.

But-3-en-2-yl but-3-enoate (S12). TLC (cyclohexane : EtOAc = 20 : 1): R_f = 0.40; **Yield**: 3.67 g, 26.2 mmol (66%); **HRMS** (APCI): calculated for $C_8H_{13}O_2^+$ 141.0910, found 141.0909; **IR** (diamond ATR): $\tilde{\nu}$ / cm^{-1} = 3086 (w), 2985 (m), 2935 (w), 1733 (s), 1644 (w), 1424 (w), 1252 (m), 1172 (s), 1047 (m), 991 (m), 920 (s); **^{1}H-NMR** (700 MHz, $CDCl_3$): δ (ppm) 5.92 (ddt, 1H, *J* = 16.5, 10.8, 6.9 Hz), 5.83 (ddd, 1H, *J* = 17.4, 10.6, 5.9 Hz), 5.36 (m, 1H), 5.23 (dt, 1H, *J* = 17.2, 1.4 Hz), 5.17 (dq, 1H, *J* = 6.8, 1.5 Hz), 5.15 (t, 1H, *J* = 1.5 Hz), 5.13 (dt, 1H, *J* = 10.6, 1.3 Hz), 3.08 (dt, 2H, *J* = 7.0, 1.5 Hz), 1.31 (3H, d, *J* = 6.6 Hz); **^{13}C-NMR** (176 MHz, $CDCl_3$) δ (ppm) 170.8, 137.7, 130.5, 118.6, 116.0, 71.3, 39.5, 20.0.

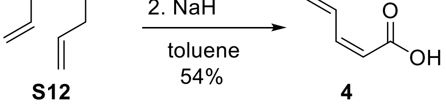

S12 **4**

To a solution of **S12** (1.12 g, 8.0 mmol) dissolved in toluene (60 mL) was added Grubbs II catalyst (67.9 mg, 0.08 mmol) at 65 °C. NaH (60 wt.-% in mineral oil, 0.48 g, 12.0 mmol) was added after 1.5 h. The solution was stirred for another 3 h at the same temperature and then cooled to room temperature. The reaction was quenched by adding water (30 mL) and HCl (aq., 1 M, 10 mL). After extraction of the mixture with diethyl ether (3 × 40 mL), the organic layer was dried over $MgSO_4$ and concentrated under reduced pressure. The residue was purified by column chromatography on silica gel (cyclohexane : diethyl ether = 3 : 1) to give **4** as colorless oil.

(2*Z*,4*E*)-Hexa-2,4-dienoic acid (S13). TLC (cyclohexane : diethyl ether = 2 : 1): R_f = 0.35; **Yield**: 0.48 g, 4.3 mmol (54%); **HRMS** (APCI): calculated for $C_6H_9O_2^+$ 113.0597, found 113.0596; **IR** (diamond ATR): $\tilde{\nu}$ / cm^{-1} = 3057 (w), 2855 (w), 1684 (s), 1637 (m), 1602

(m), 1435 (m), 1250 (m), 1227 (m), 962 (w), 840 (w); **^{1}H-NMR** (700 MHz, $CDCl_3$): *δ* (ppm) 7.36 (m, 1H), 6.65 (t, 1H, *J* = 11.5 Hz), 6.15 (m, 1H), 5.58 (dq, 1H, *J* = 11.4, 0.9 Hz), 1.90 (dd, 3H, *J* = 6.9, 1.7 Hz); **^{13}C-NMR** (176 MHz, $CDCl_3$) *δ* (ppm) 172.3, 147.7, 141.9, 128.6, 114.7, 18.9.

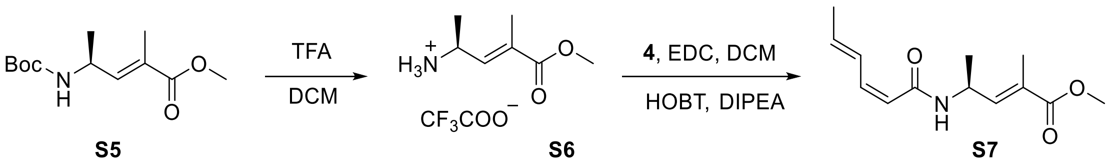

To a solution of TFA / CH_2Cl_2 (2 : 3, 2.5 mL) was added **S5** (106.9 mg, 0.44 mmol). The reaction was stirred at room temperature for 30 min and then concentrated under reduced pressure to give **S6** as white solid which was used directly in the next step.

To a solution of **S6** and **4** (49.3 mg, 0.44 mmol) in CH_2Cl_2 (5 mL) was added HOBT (70.7 mg, 0.44 mmol) and *N*,*N*-diisopropylethylamine (76.6 µL, 0.44 mmol). EDC was added sequentially at 0 °C. The mixture was stirred at 0 °C for 30 min followed by room temperature overnight. Then the solution was diluted with EtOAc (50 mL) and washed with saturated NH_4Cl solution (10 mL), $NaHCO_3$ solution (10 mL), water (10 mL) and brine (10 mL). After drying over $MgSO_4$ and concentration under reduced pressure, the residue was purified by column chromatography on silica gel (cyclohexane : EtOAc = 3 : 1). Amide **S7** was obtained as colorless oil.

Methyl (*S*,*E*)-4-((2*Z*,4*E*)-hexa-2,4-dienamido)-2-methylpent-2-enoate (S7). TLC (cyclohexane : EtOAc = 1 : 1): R_f = 0.50; **Yield**: 88.1 mg, 0.37 mmol (84%) (2 steps); **Optical rotation**: $[\alpha]_D^{25}$ = –193.1 (*c* 0.14 , MeOH); **HRMS** (ESI): calculated for $C_{13}H_{20}NO_3^+$ 238.1438, found 238.1442; **IR** (diamond ATR): ṽ / cm^{-1} = 3289 (m), 2977 (m), 1717 (s), 1651 (s), 1532 (s), 1437 (m), 1256 (s), 1145 (m), 1084 (m), 749 (m); **^{1}H-NMR** (500 MHz, CD_3OD): *δ* (ppm) 7.41 (m, 1H), 6.59 (dq, 1H, *J* = 9.0, 1.5 Hz), 6.43 (t, 1H, *J* = 11.3 Hz), 6.03 (dqt, 1H, *J* = 15.1, 6.9, 0.9 Hz), 5.58 (dq, 1H, *J* = 11.4, 0.8 Hz), 4.81 (dq, 1H, *J* = 9.0, 6.8 Hz), 3.76 (s, 3H), 1.95 (d, 3H, *J* = 1.5 Hz), 1.86 (dd, 3H, *J* = 6.9, 1.7 Hz), 1.27 (d,3H, *J* = 6.9 Hz); **^{13}C-NMR** (126 MHz, CD_3OD) *δ* (ppm) 169.9, 168.2, 143.9, 142.7, 139.0, 129.7, 129.2, 119.0, 52.4, 44.4, 20.2, 18.6, 12.8.

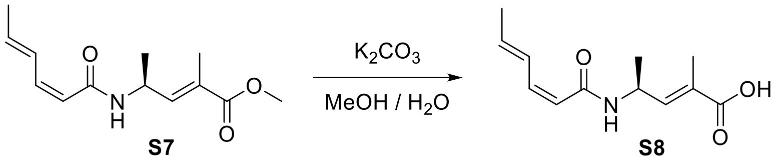

K_2CO_3 (215.6 mg, 1.56 mmol) was added to a solution of **S7** (74.0 mg, 0.31 mmol) in MeOH / H_2O (1 : 2, 3 mL). The reaction mixture was stirred at 40 °C for 4 h until **S7** was consumed completely. After the treatment with HCl solution (1 M) dropwise until pH = 2 was reached, an excess of aceton was added to precipitate the salt and filtration was conducted. The filtrate was evaporated under reduced pressure to give **S8** as white solid.

(*S*,*E*)-4-((2*Z*,4*E*)-hexa-2,4-dienamido)-2-methylpent-2-enoic acid (S8). TLC (cyclohexane : EtOAc = 1 : 1): R_f = 0.42; **Yield**: 67.1 mg, 0.30 mmol (100%); **Optical rotation**: $[\alpha]_D^{25}$ = –129.3 (*c* 0.40 , MeOH); **HRMS** (ESI): calculated for $C_{12}H_{18}NO_3^+$ 224.1281, found 224.1284; **IR** (diamond ATR): ṽ / cm^{-1} = 3271 (m), 2976 (m), 2931 (m), 1691 (s), 1649 (s), 1535 (s), 1252 (s), 1233 (s), 1157 (m), 999 (w); **^{1}H-NMR** (500 MHz, CD_3OD): *δ* (ppm) 7.41 (dddt, 1H, *J* = 15.6, 11.2, 3.0, 1.6 Hz), 6.61 (dq, 1H, *J* = 9.0, 1.5 Hz), 6.42 (t, 1H, *J* = 11.3 Hz), 6.02 (m, 1H), 5.59 (dt, 1H, *J* = 11.6, 1.0 Hz), 4.82 (dq, 1H, *J* = 8.9, 6.9 Hz), 1.93 (d, 3H, *J* = 1.5 Hz), 1.86 (dd, 3H, *J* = 6.8, 1.7 Hz), 1.27 (d, 3H, *J* = 6.8 Hz); **^{13}C-NMR** (126 MHz, $CDCl_3$) *δ* (ppm) 171.3, 168.2, 143.7, 142.6, 138.9, 129.7, 129.6, 119.1, 44.5, 20.3, 18.6, 12.7.

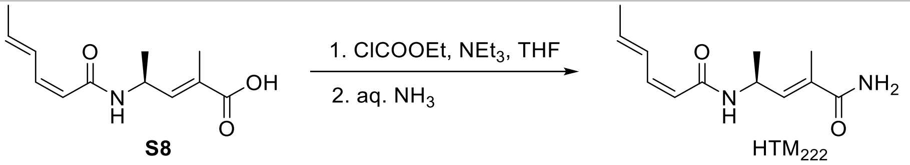

S8 → HTM$_{222}$

Triethylamine (45.2 µL, 0.32 mmol) and ethyl chloroformate (28.4 µL, 0.30 mmol) were added to a solution of **S8** (60.0 mg, 0.27 mmol) dissolved in THF (1 mL) at 0 °C. The reaction mixture was stirred at 0 °C for 30 min and then aq. NH_3 (25%, 0.1 mL, 1.1 mmol) was added at the same temperature. After 15 min, the reaction mixture was diluted with H_2O (10 mL) and extracted with CH_2Cl_2 (3 × 10 mL). The combined organic phases were dried over $MgSO_4$ and then concentrated under reduced pressure. The residue was purified by column chromatography on silica gel (EtOAc) to give sarmentosamide (HTM$_{222}$) as white solid.

(2*Z*,4*E*)-N-((*S*,*E*)-5-Amino-4-methyl-5-oxopent-3-en-2-yl)hexa-2,4-dienamide (sarmentosamide, HTM$_{222}$). TLC (EtOAc): R_f = 0.13; **Yield**: 38.0 mg, 0.17 mmol (63%); **Optical rotation**: $[\alpha]_D^{25}$ = –194.0 (*c* 0.28, MeOH); **HRMS** (ESI): calculated for $C_{12}H_{19}N_2O_2^+$ 223.1441, found 223.1445; **IR** (diamond ATR): ṽ / cm^{-1} = 3287 (s), 2927 (m), 2854 (w), 1645 (s), 1601 (s), 1533 (s), 1377 (m), 1228 (m), 998 (w), 835 (w); **^{1}H-NMR** (700 MHz, CD_3OD): *δ* (ppm) 7.42 (dddt, 1H, *J* = 14.6, 11.2, 2.7, 1.6 Hz), 6.43 (1H, t, *J* = 11.4 Hz), 6.26 (dq, 1H, *J* = 8.8, 1.4 Hz, 1H), 6.03 (1H, dqt, *J* = 15.2, 6.8, 0.8 Hz), 5.59 (dq, 1H, *J* = 11.4, 0.8 Hz), 4.81 (dq, 1H, *J* = 8.7, 6.8 Hz), 1.96 (d, 3H, *J* = 1.5 Hz), 1.86 (dd, 3H, *J* = 6.8, 1.4 Hz), 1.28 (d, 3H, *J* = 6.8 Hz); **^{13}C-NMR** (176 MHz, CD_3OD) *δ* (ppm) 174.2, 168.2, 142.6, 139.0, 138.9, 132.3, 129.8, 119.1, 44.3, 20.5, 18.6, 13.2.

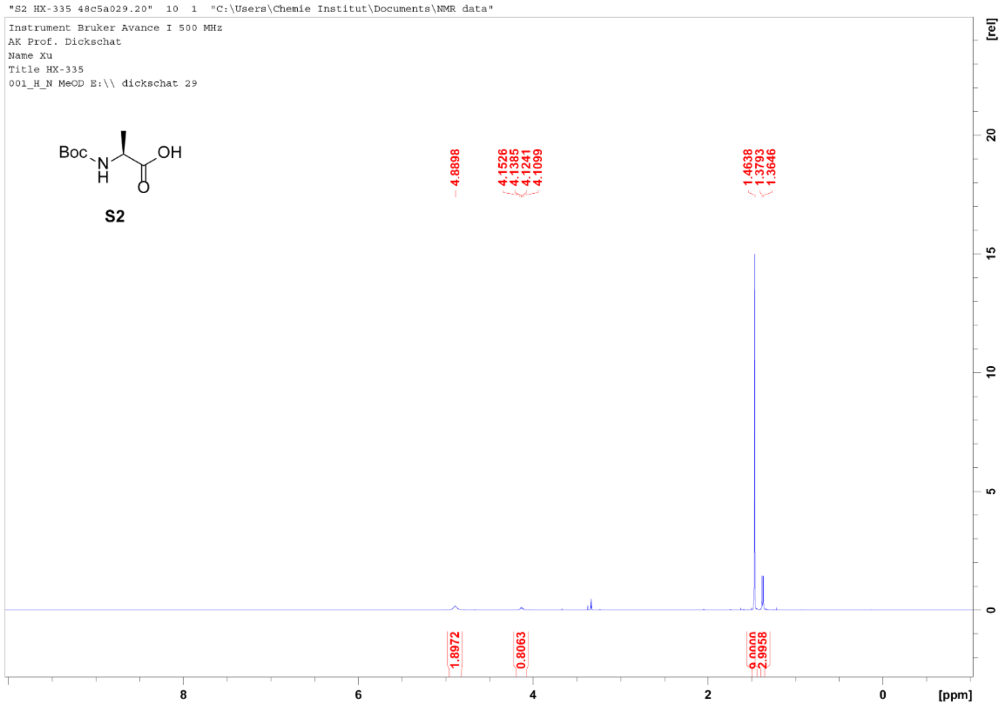

Figure S10. ^{1}H NMR spectrum (500 MHz, CD_3OD) of **S2**.

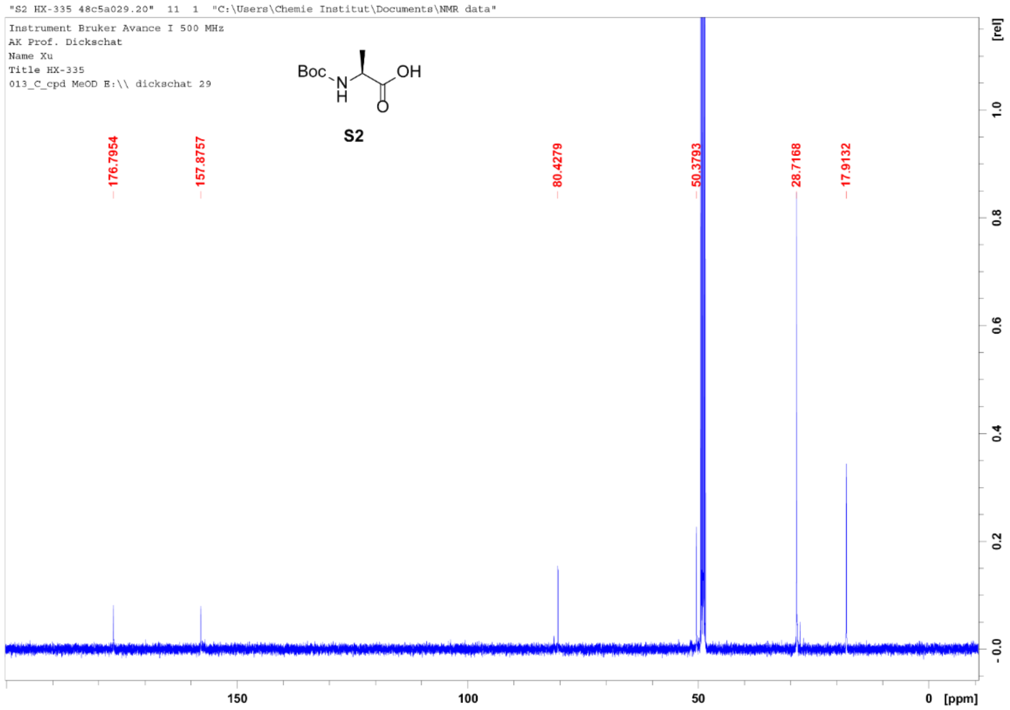

Figure S11. ^{13}C NMR spectrum (126 MHz, CD_3OD) of **S2**.

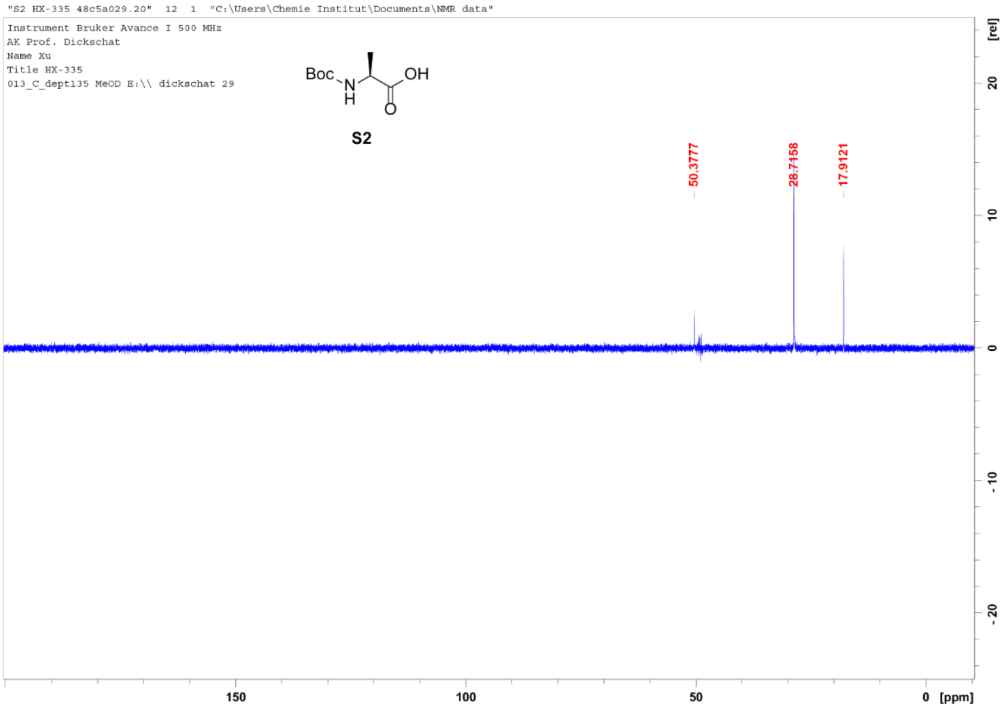

Figure S12. ^{13}C-DEPT-135 NMR spectrum (126 MHz, CD_3OD) of **S2**.

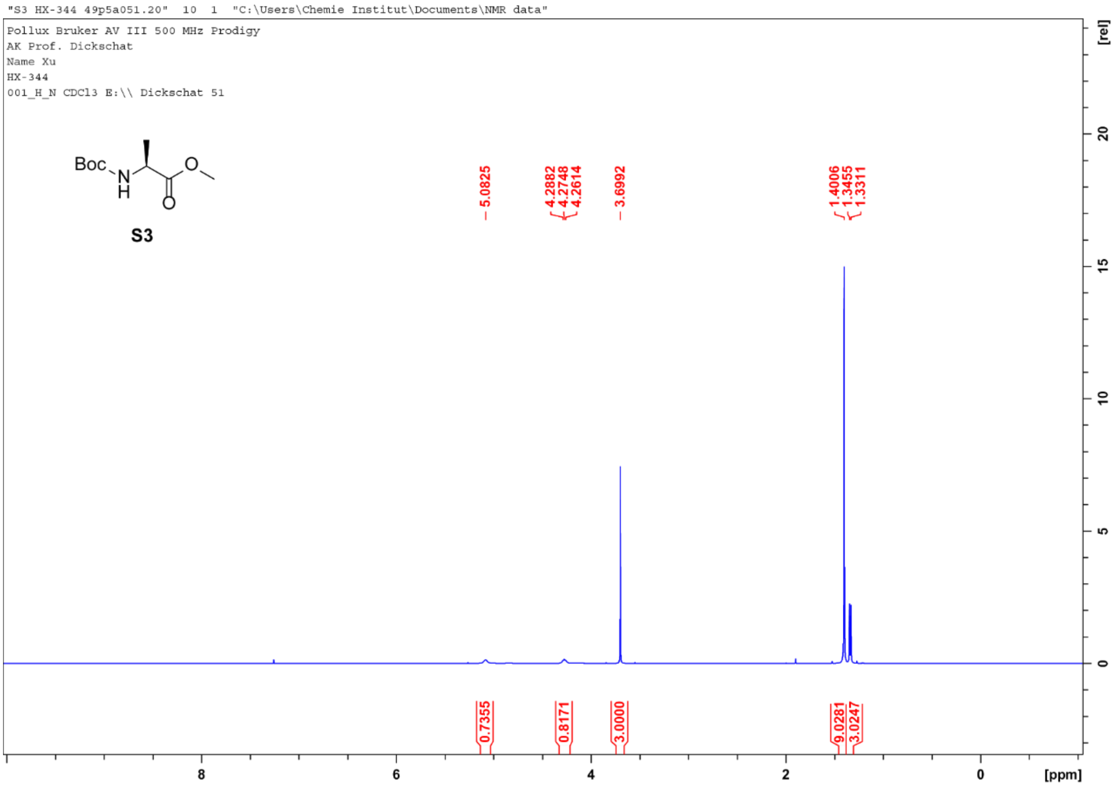

Figure S13. ^{1}H NMR spectrum (500 MHz, $CDCl_3$) of **S3**.

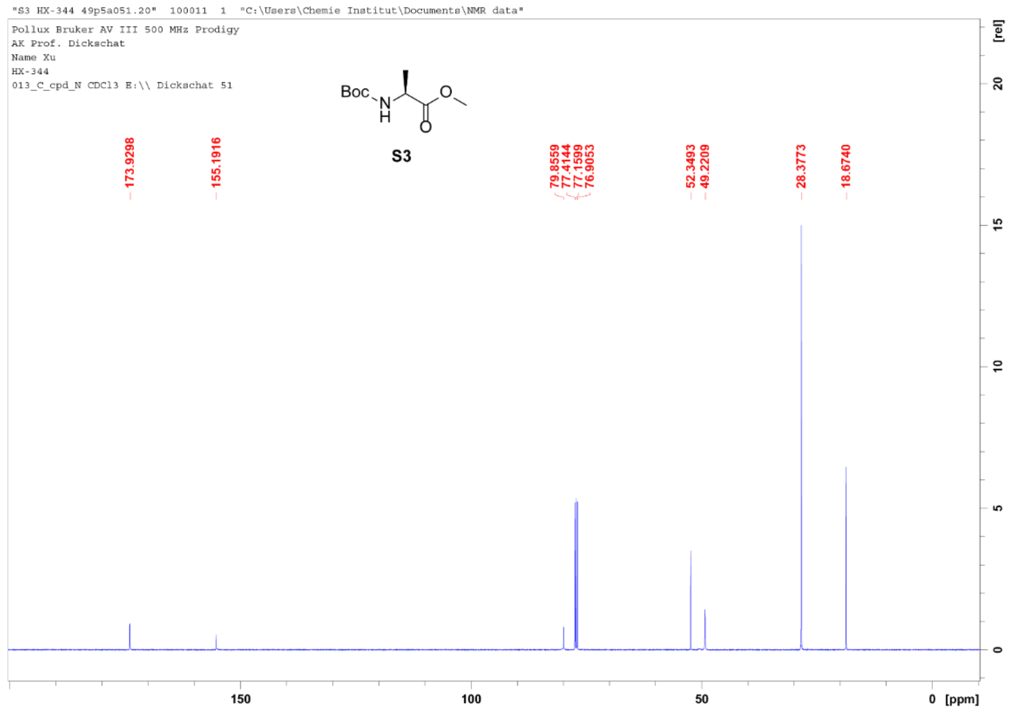

Figure S14. ^{13}C NMR spectrum (126 MHz, $CDCl_3$) of **S3**.

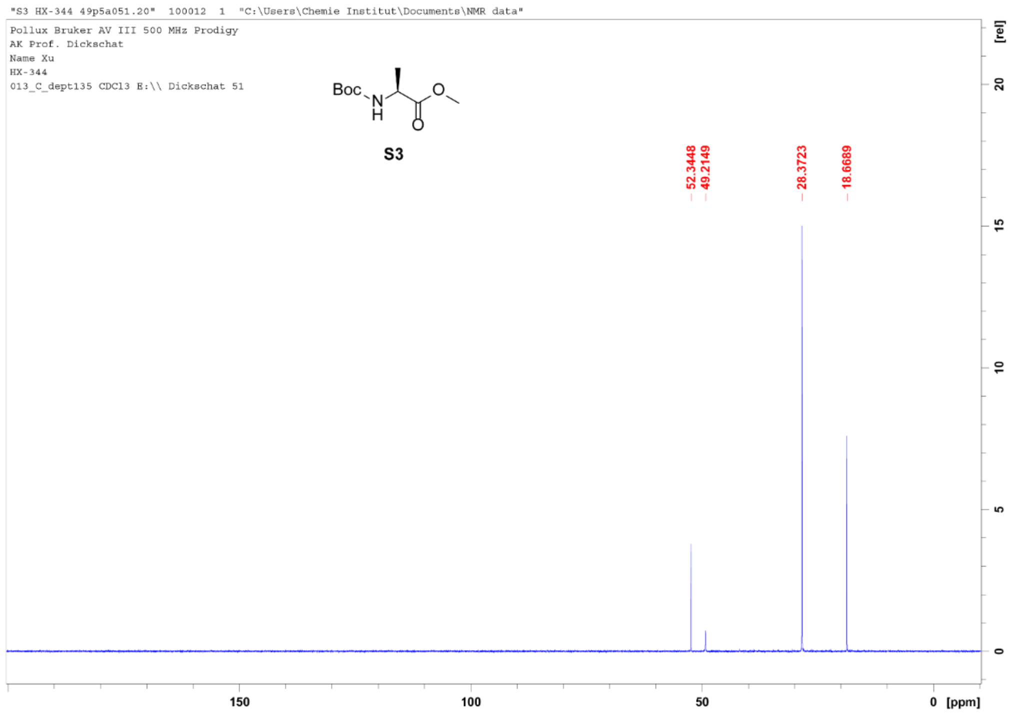

Figure S15. ^{13}C-DEPT-135 NMR spectrum (126 MHz, $CDCl_3$) of **S3**.

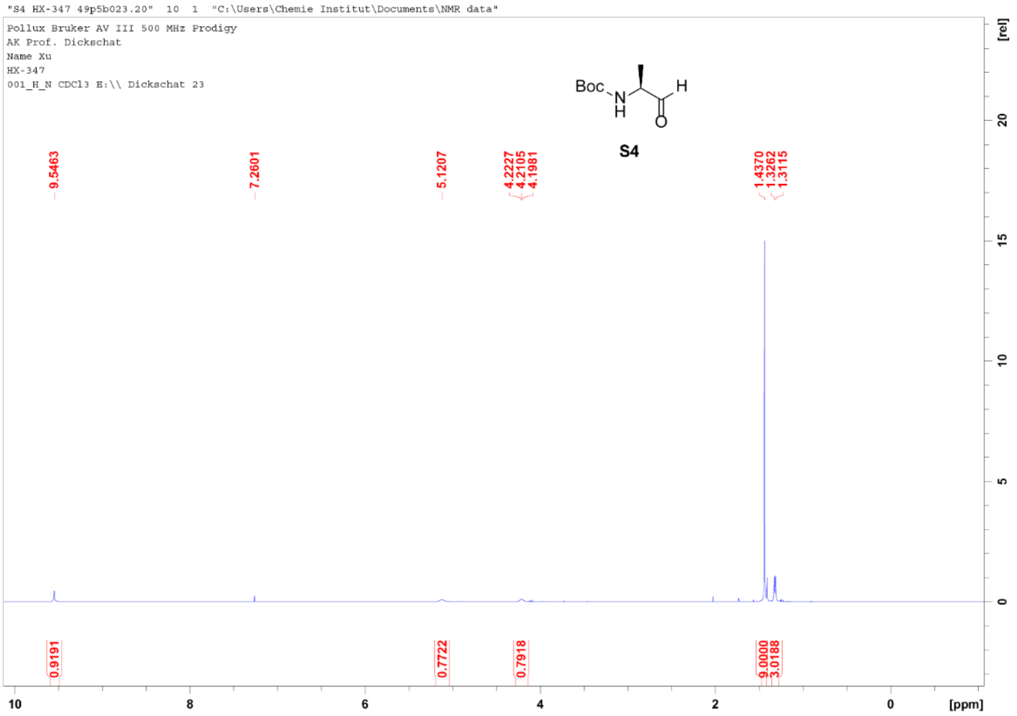

Figure S16. ^{1}H NMR spectrum (500 MHz, $CDCl_3$) of **S4**.

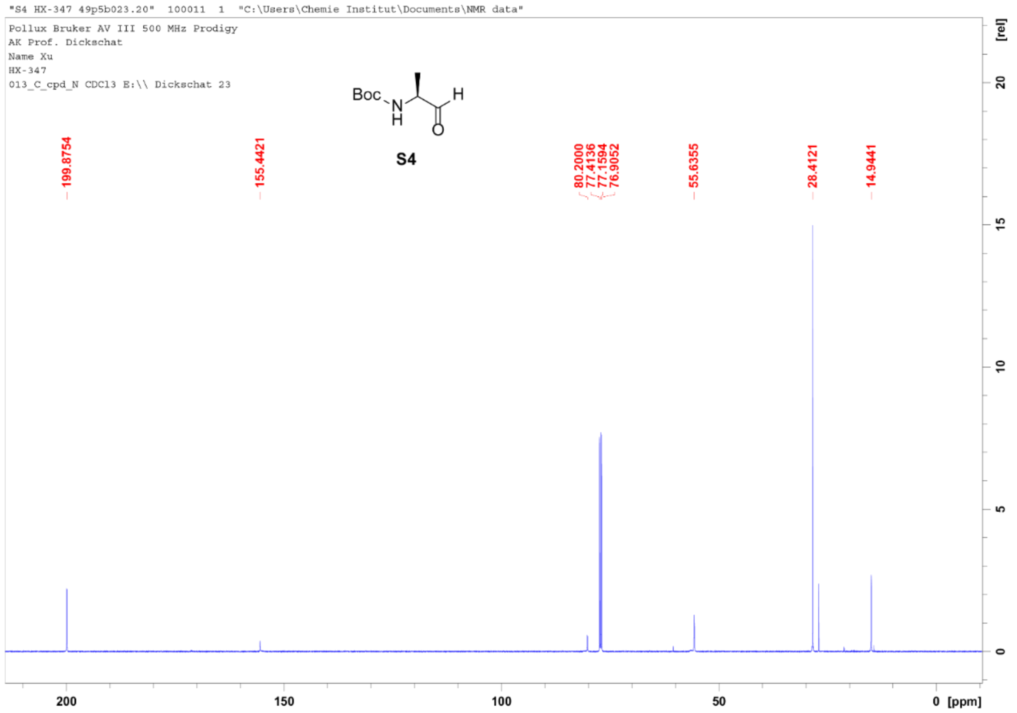

Figure S17. ^{13}C NMR spectrum (126 MHz, $CDCl_3$) of **S4**.

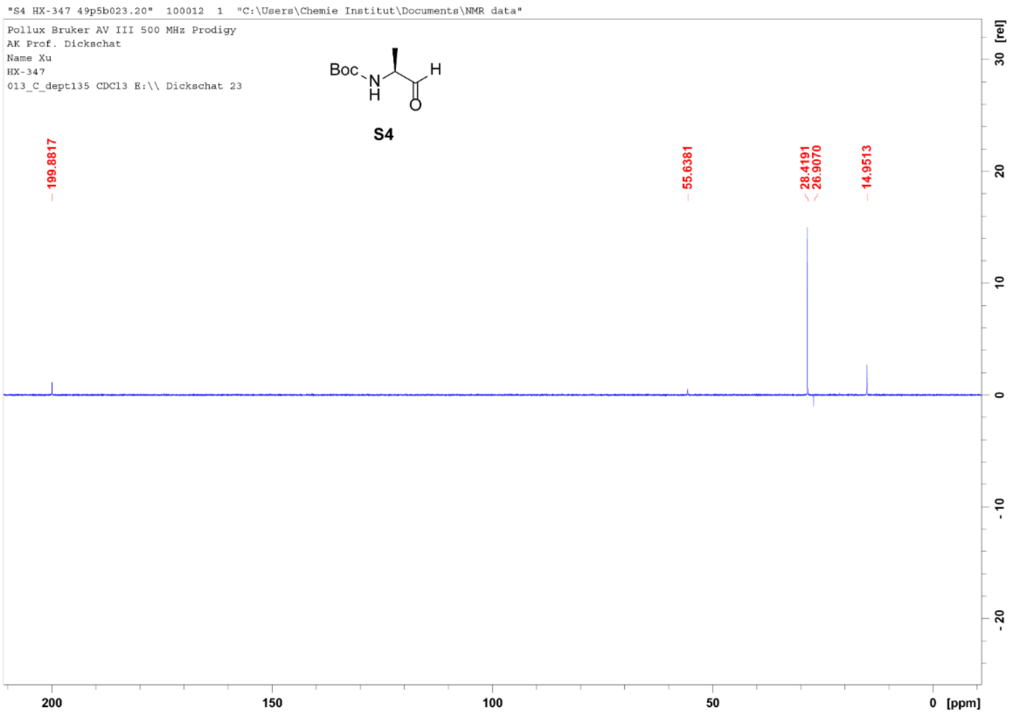

Figure S18. ^{13}C-DEPT-135 NMR spectrum (126 MHz, $CDCl_3$) of **S4**.

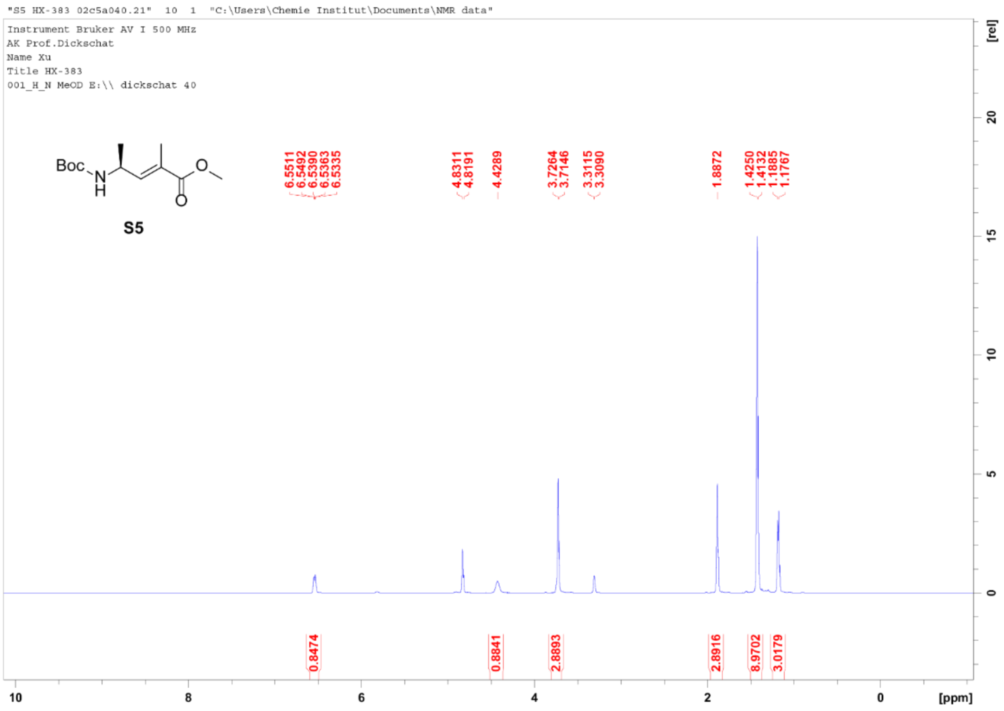

Figure S19. ^{1}H NMR spectrum (500 MHz, CD_3OD) of **S5**.

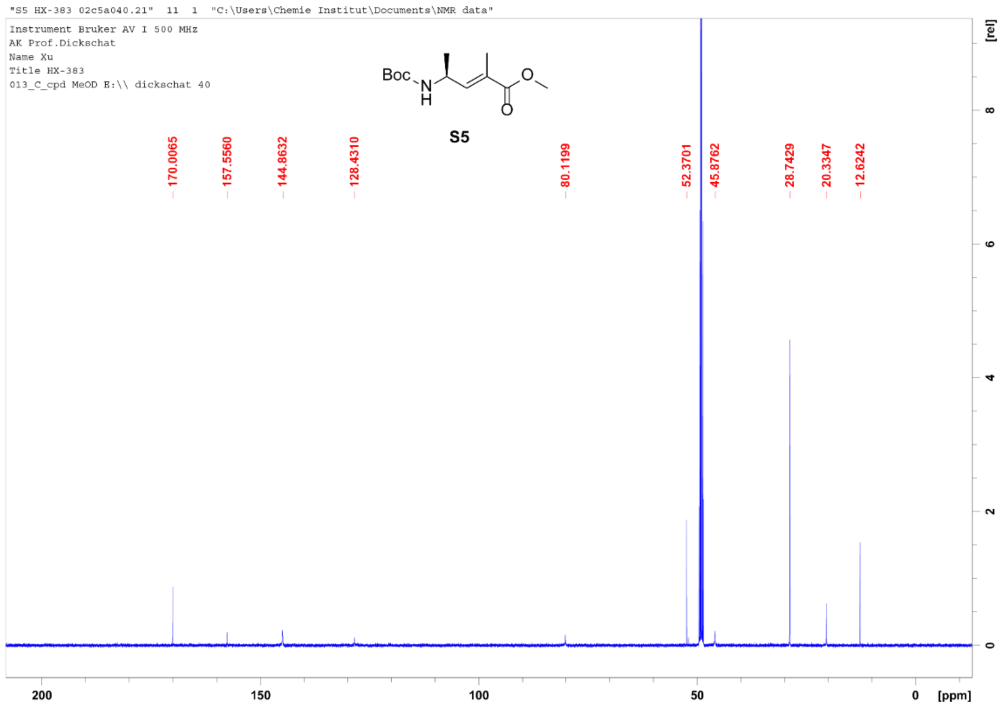

Figure S20. ^{13}C NMR spectrum (126 MHz, CD_3OD) of **S5**.

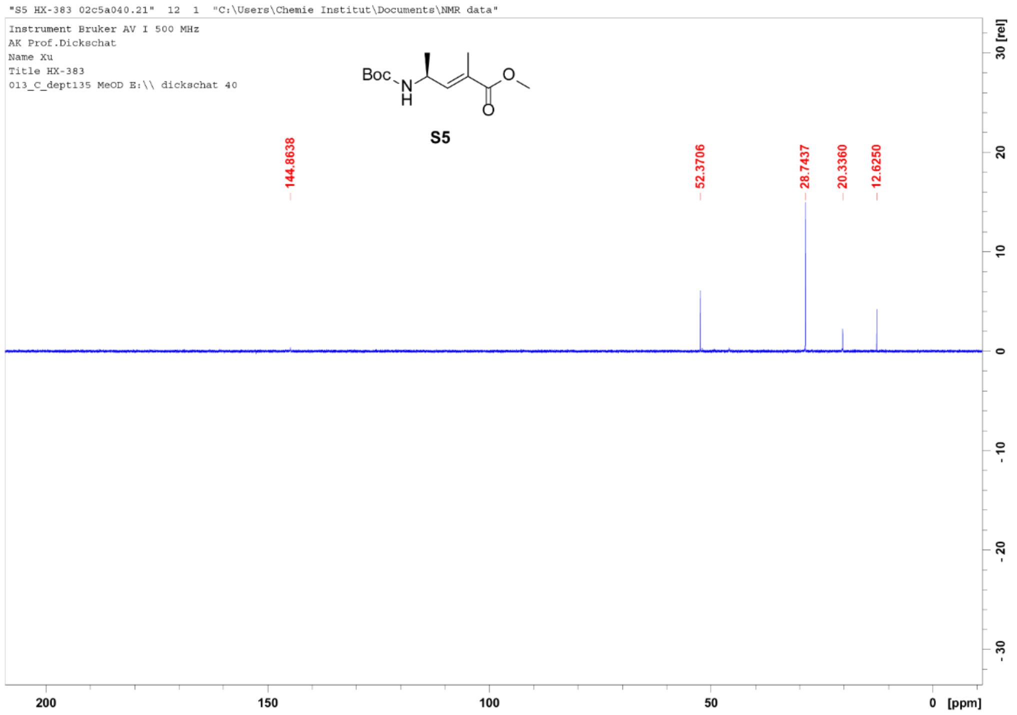

Figure S21. ^{13}C-DEPT-135 NMR spectrum (126 MHz, CD_3OD) of **S5**.

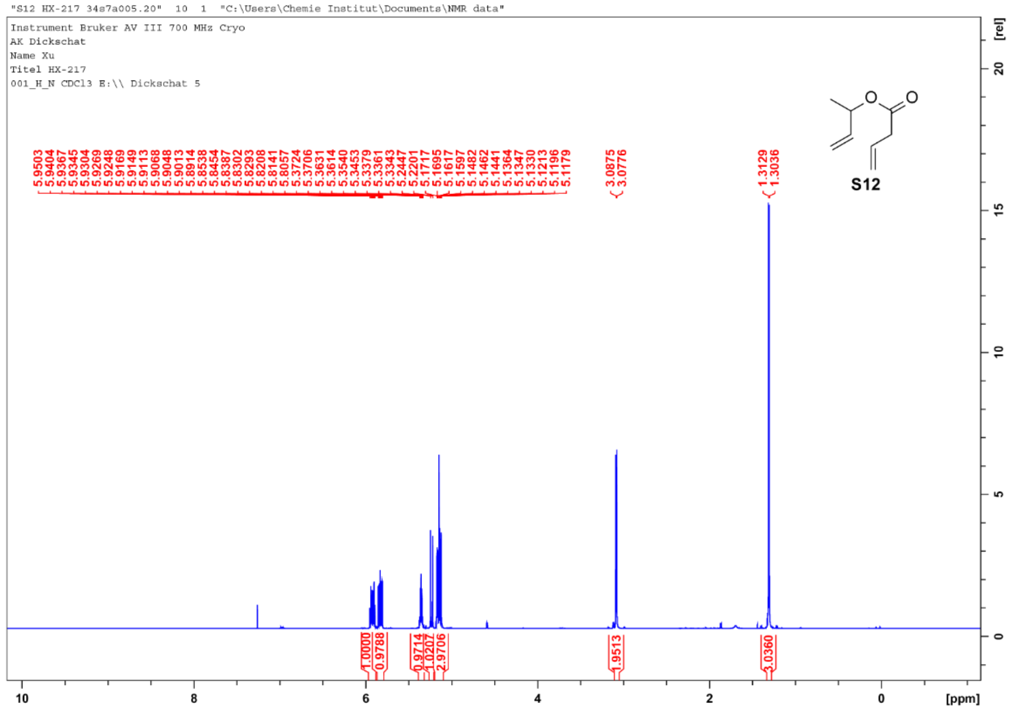

Figure S22. ^{1}H NMR spectrum (700 MHz, $CDCl_3$) of **S12**.

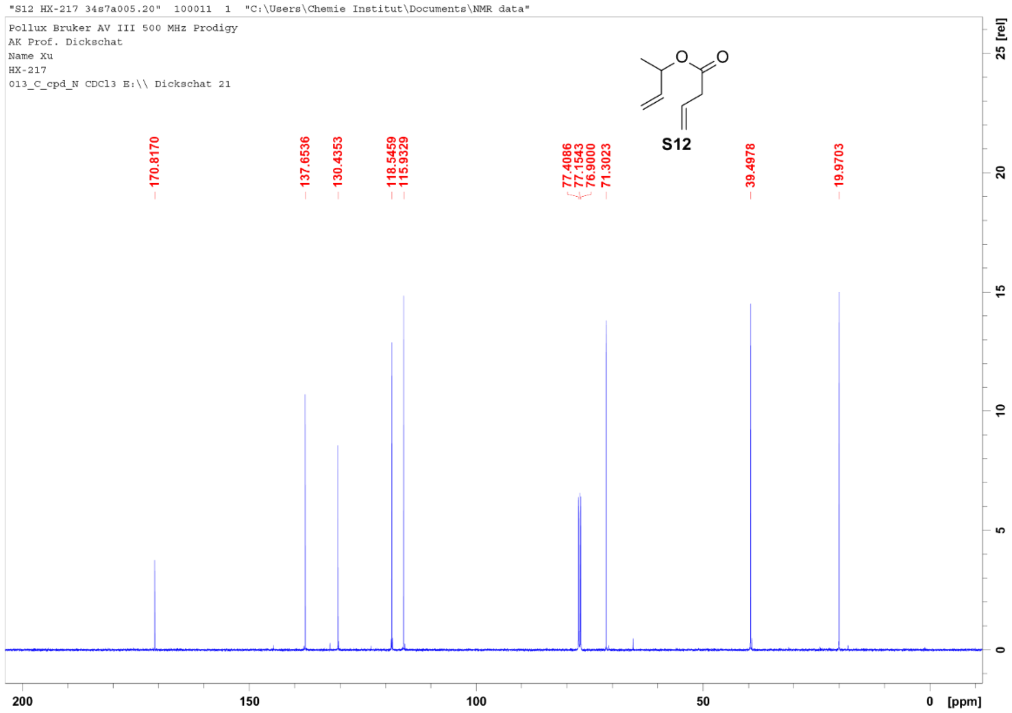

Figure S23. ^{13}C NMR spectrum (176 MHz, $CDCl_3$) of **S12**.

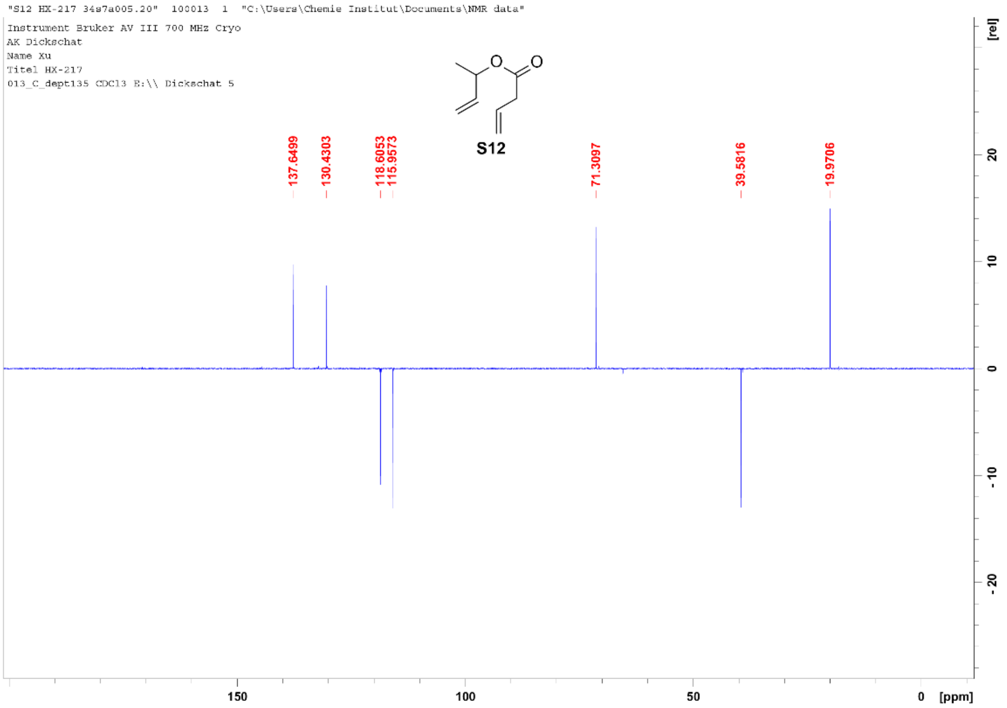

Figure S24. ^{13}C-DEPT-135 NMR spectrum (176 MHz, $CDCl_3$) of **S12**.

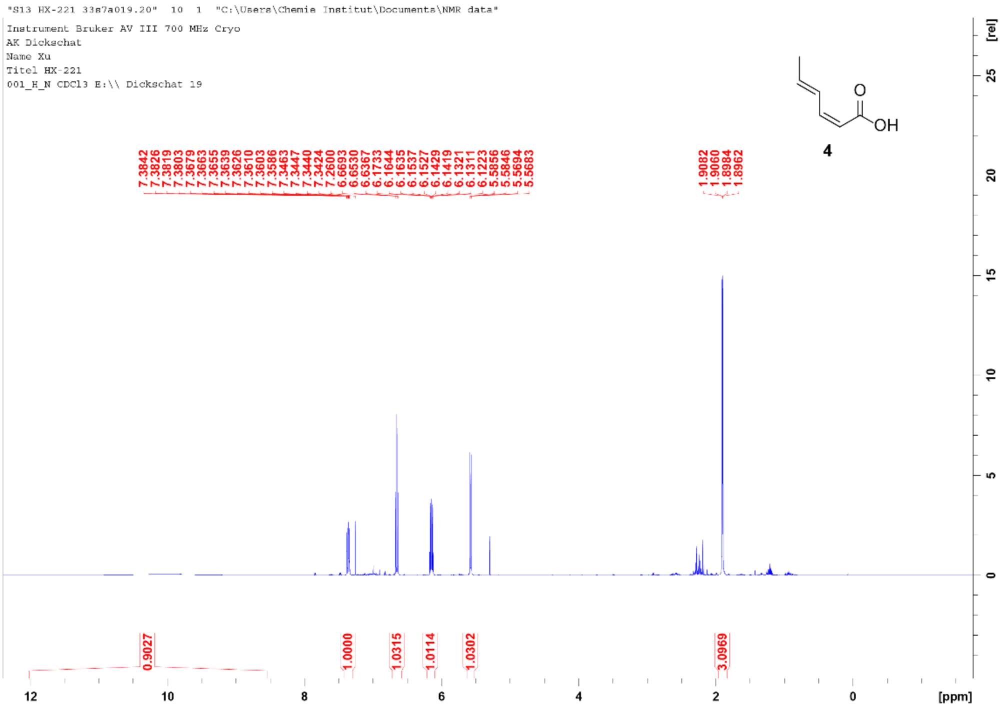

Figure S25. ^{1}H NMR spectrum (700 MHz, $CDCl_3$) of **4**.

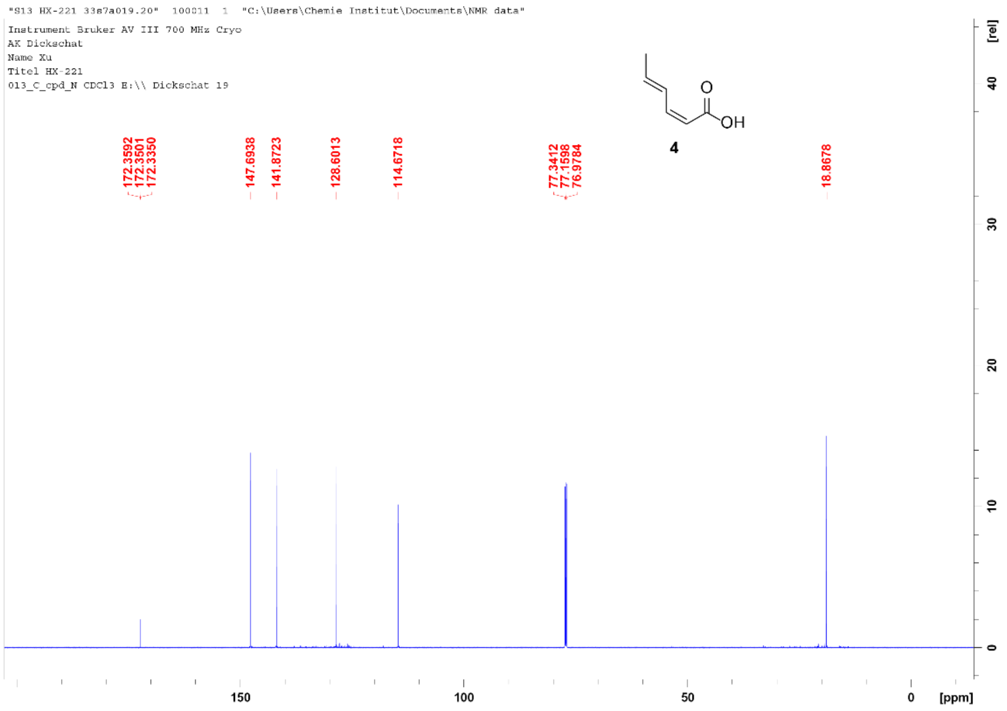

Figure S26. ^{13}C NMR spectrum (176 MHz, $CDCl_3$) of **4**.

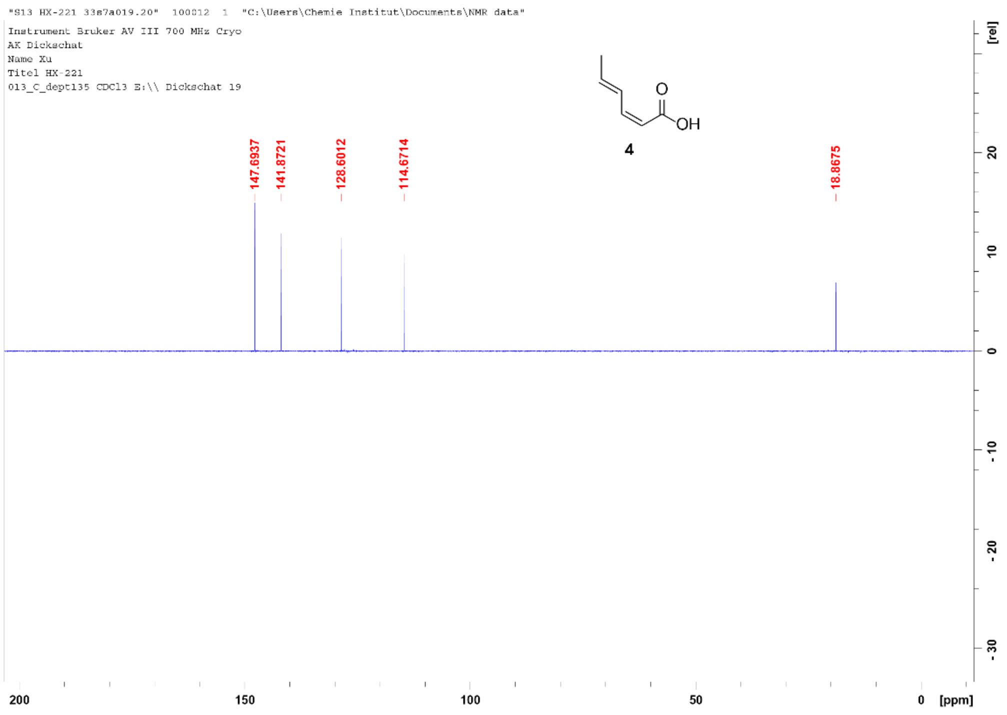

Figure S27. ^{13}C-DEPT-135 NMR spectrum (176 MHz, $CDCl_3$) of **4**.

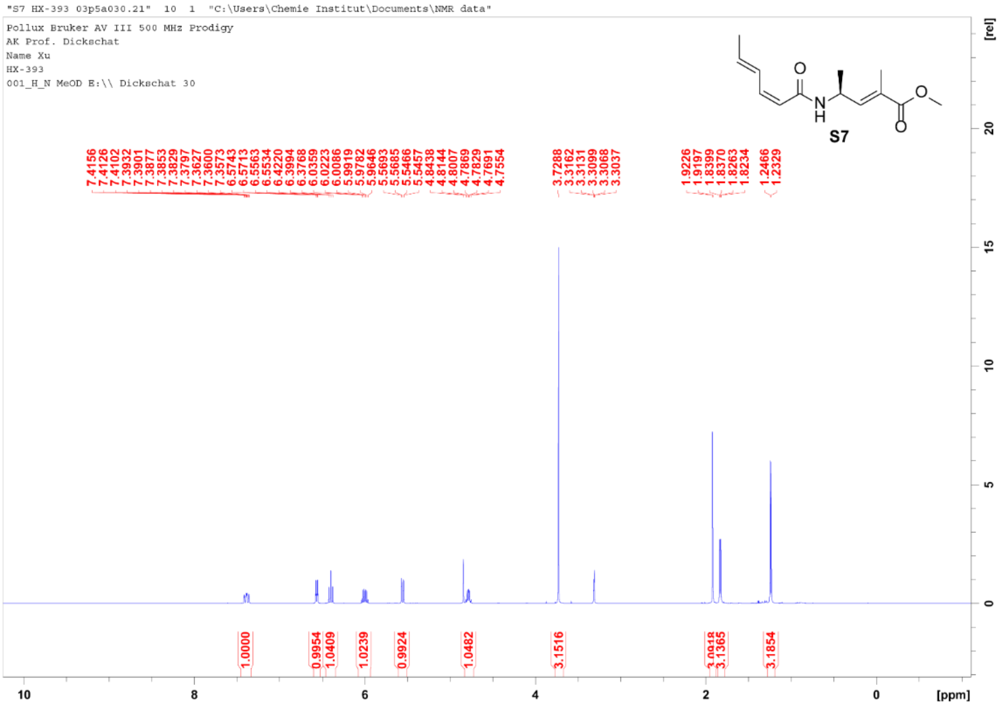

Figure S28. ^{1}H NMR spectrum (500 MHz, CD_3OD) of **S7**.

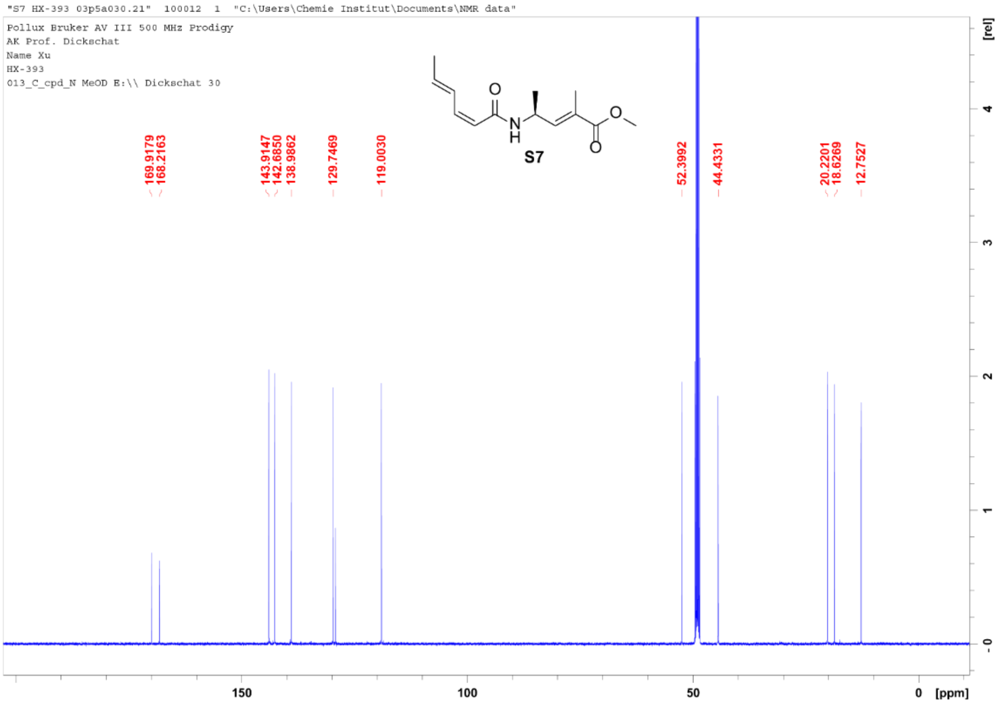

Figure S29. ^{13}C NMR spectrum (126 MHz, CD_3OD) of **S7**.

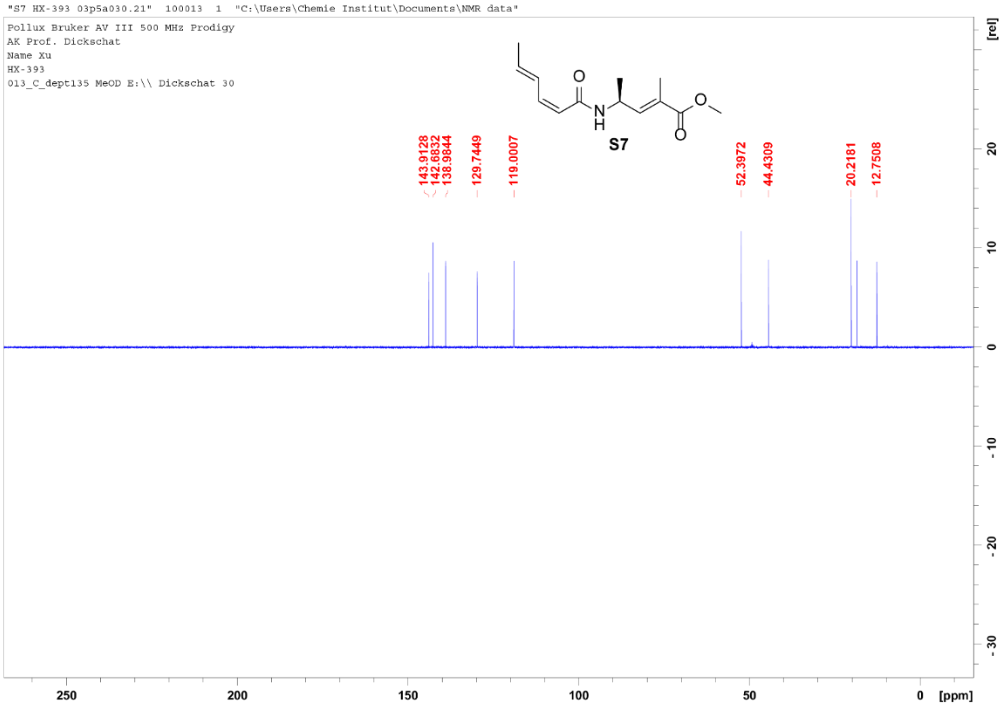

Figure S30. ^{13}C-DEPT-135 NMR spectrum (126 MHz, CD_3OD) of **S7**.

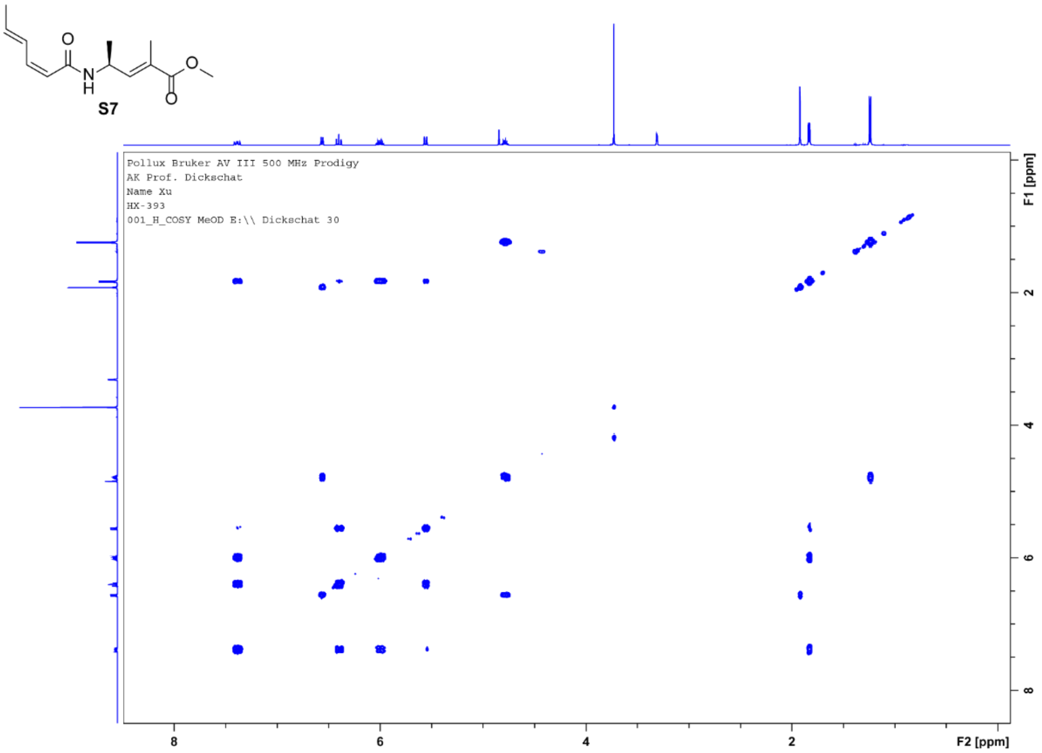

Figure S31. ^{1}H-^{1}H COSY spectrum (500 MHz, CD_3OD) of **S7**.

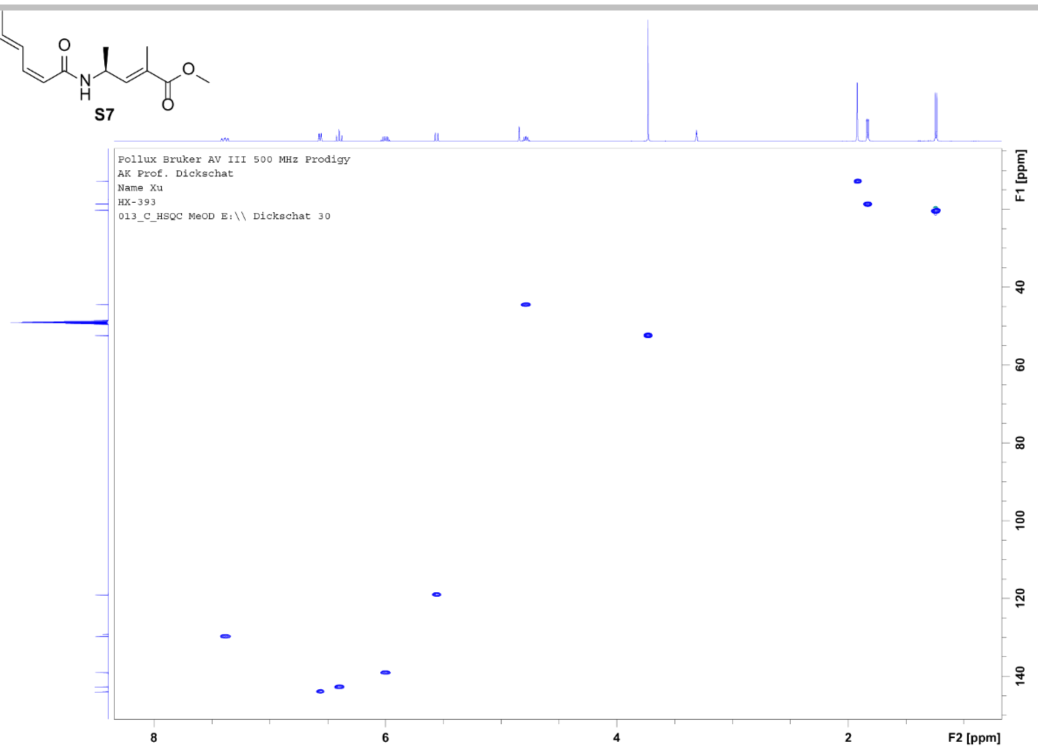

Figure S32. HSQC spectrum (CD_3OD) of **S7**.

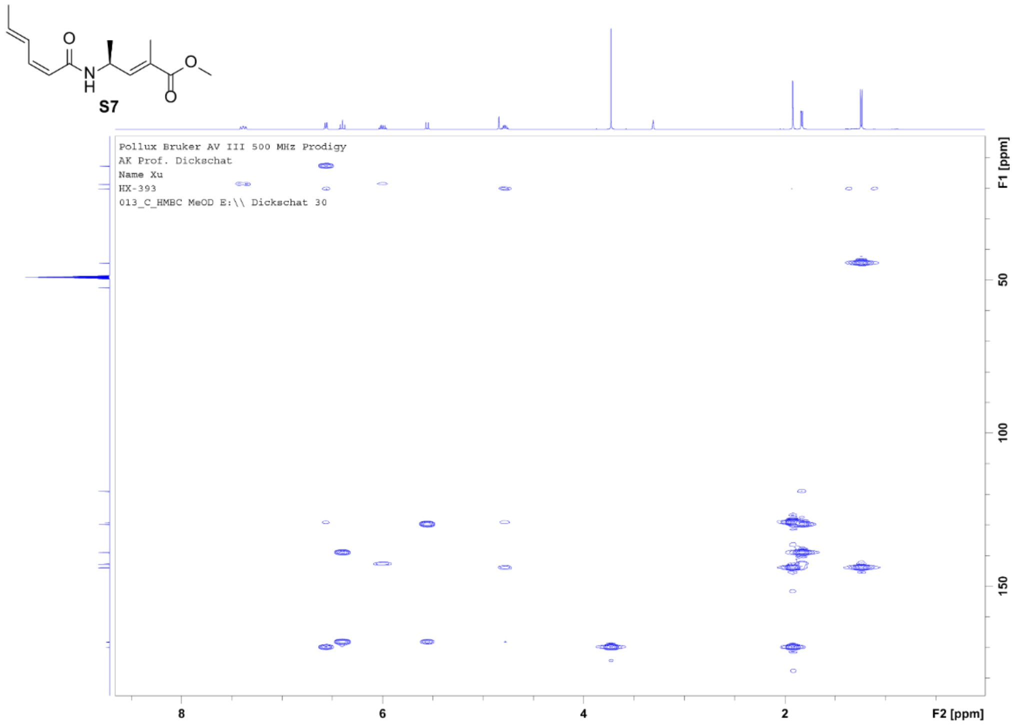

Figure S33. HMBC spectrum (CD_3OD) of **S7**.

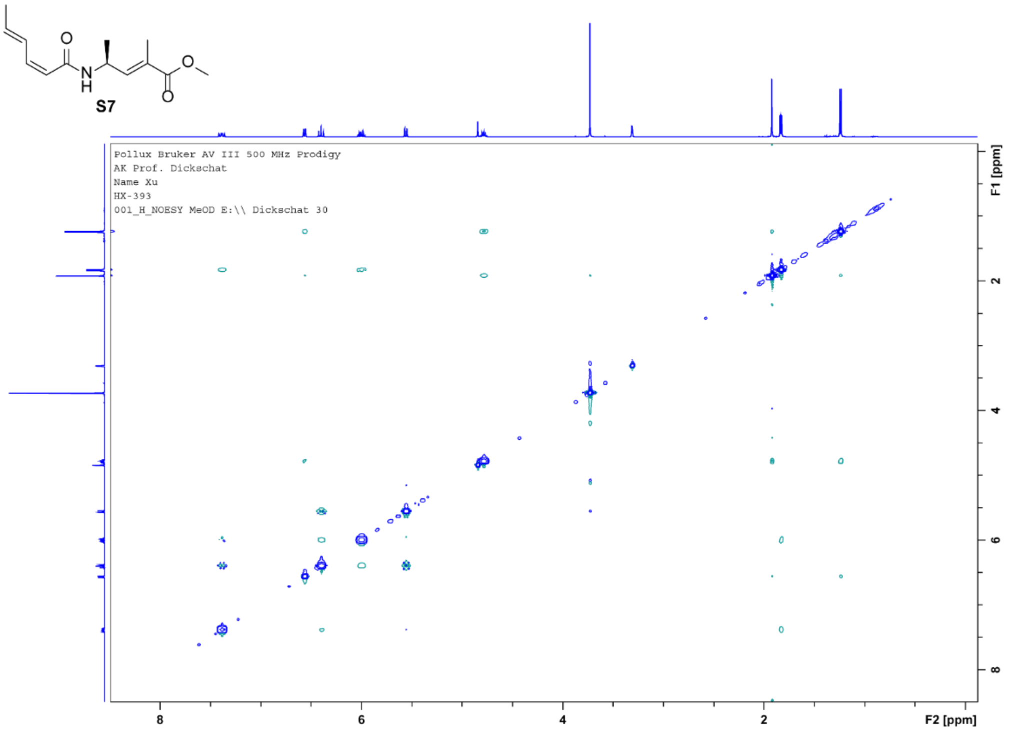

Figure S34. NOESY spectrum (CD_3OD) of **S7**.

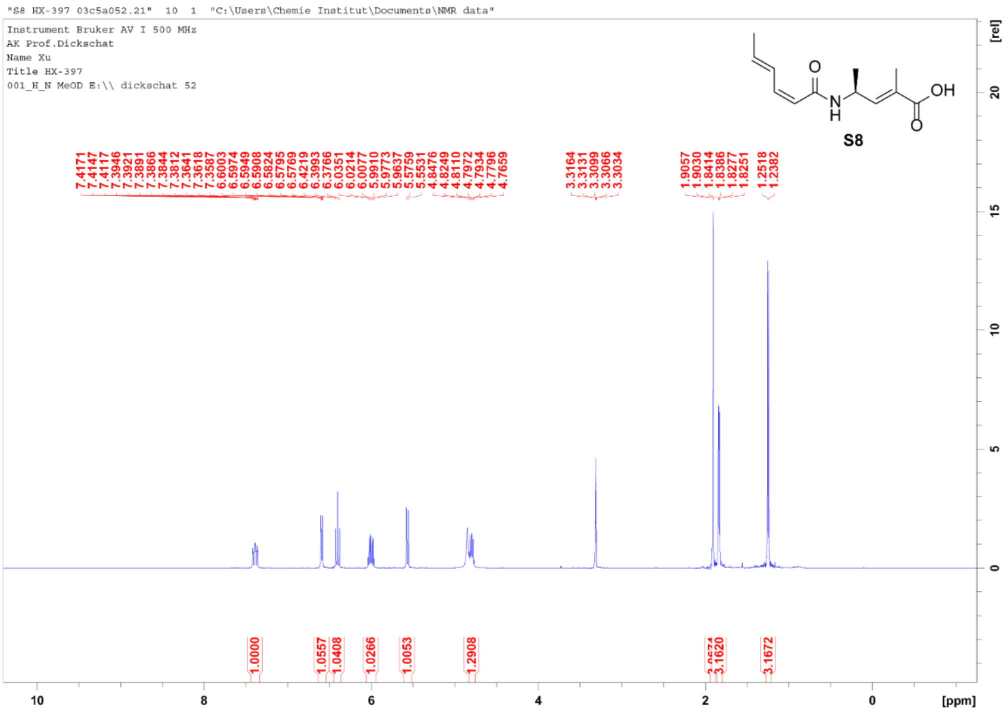

Figure S35. ^{1}H NMR spectrum (500 MHz, CD_3OD) of **S8**.

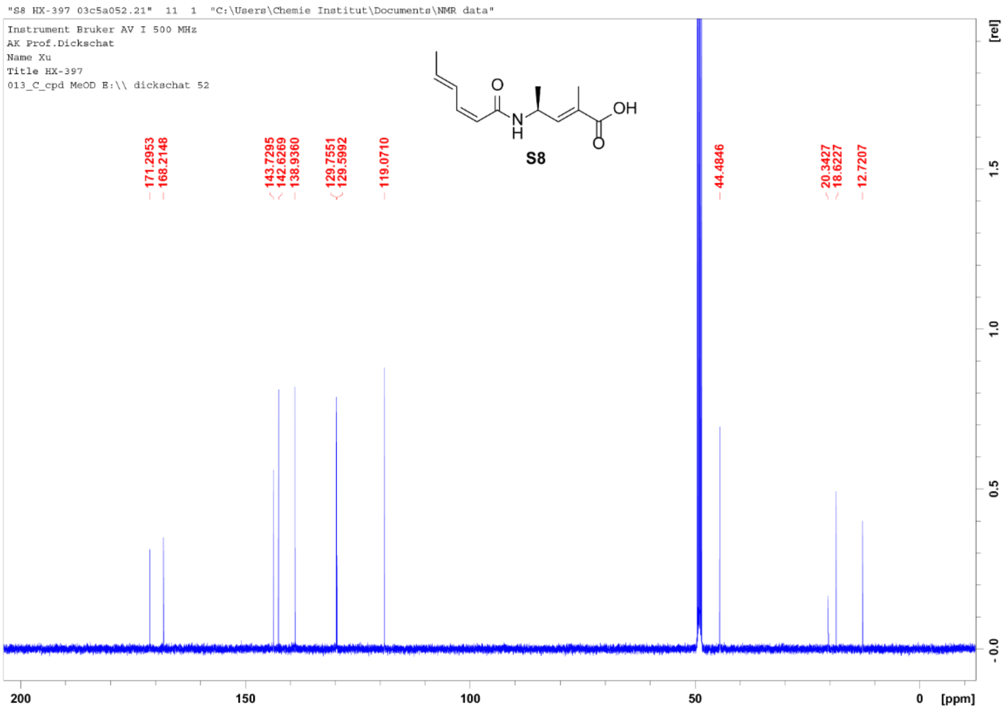

Figure S36. ^{13}C NMR spectrum (126 MHz, CD_3OD) of **S8**.

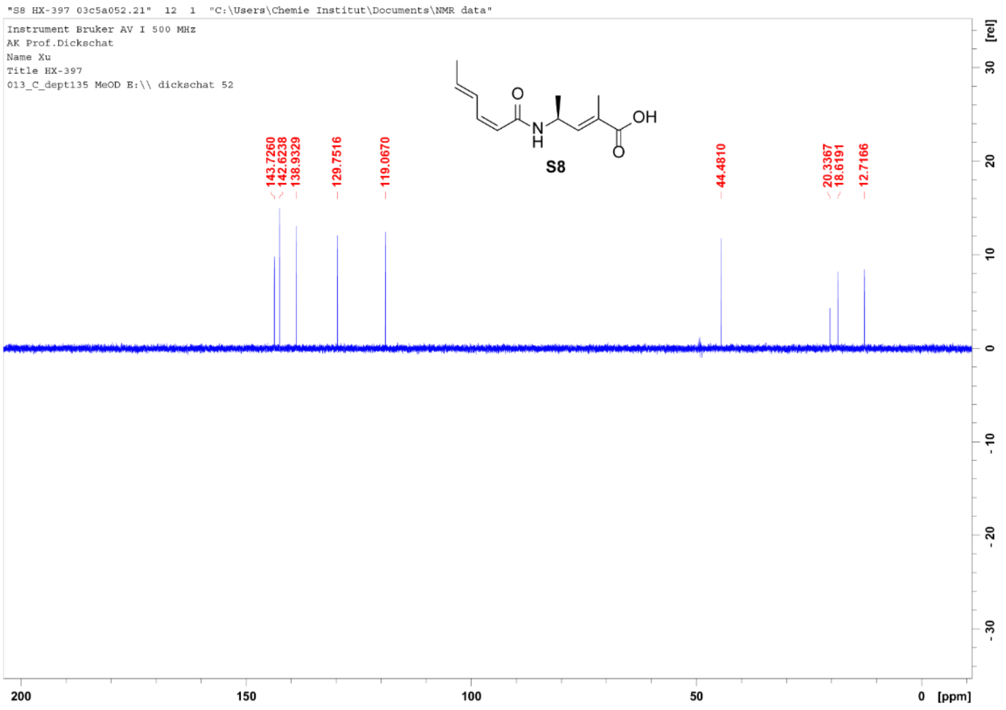

Figure S37. ^{13}C-DEPT-135 NMR spectrum (126 MHz, CD_3OD) of **S8**.

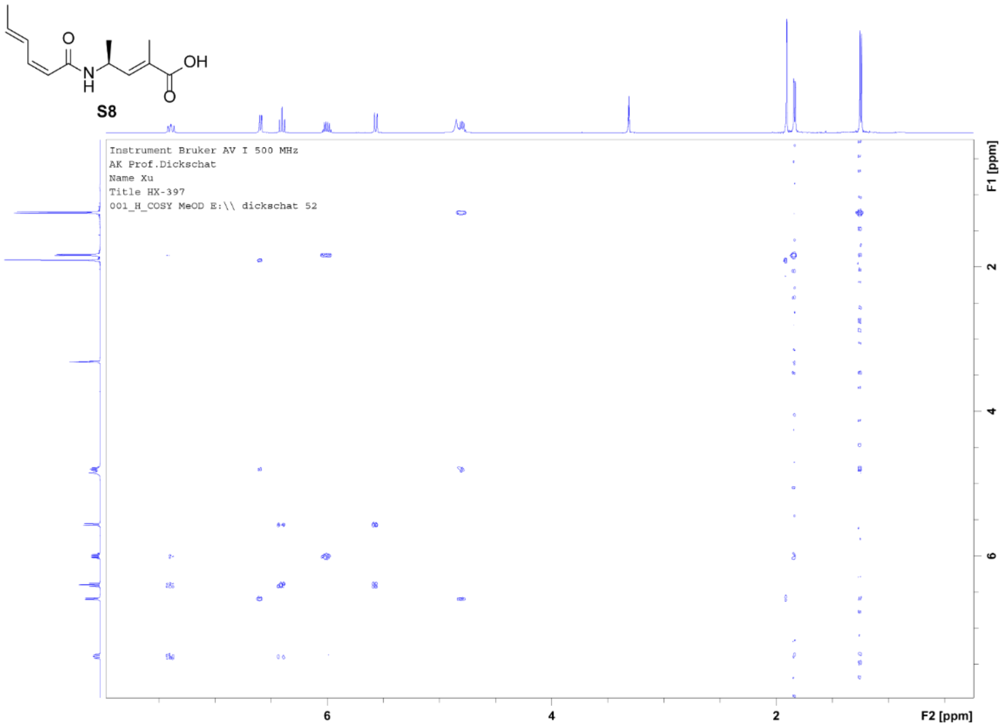

Figure S38. ^{1}H-^{1}H COSY spectrum (500 MHz, CD_3OD) of **S8**.

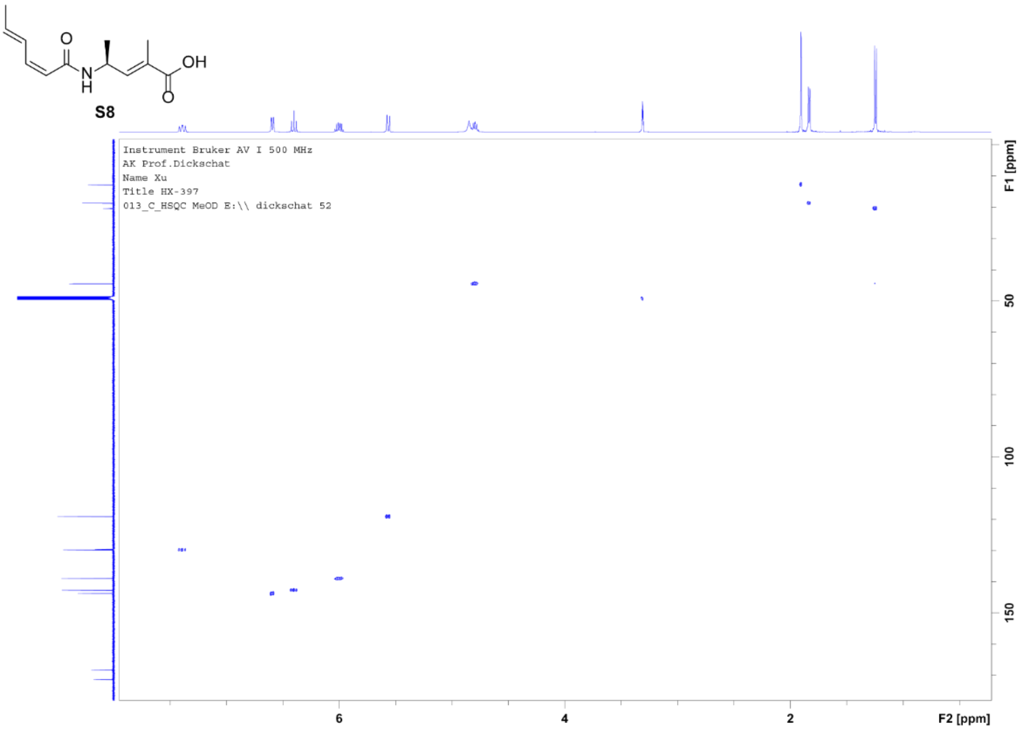

Figure S39. HSQC spectrum (CD_3OD) of **S8**.

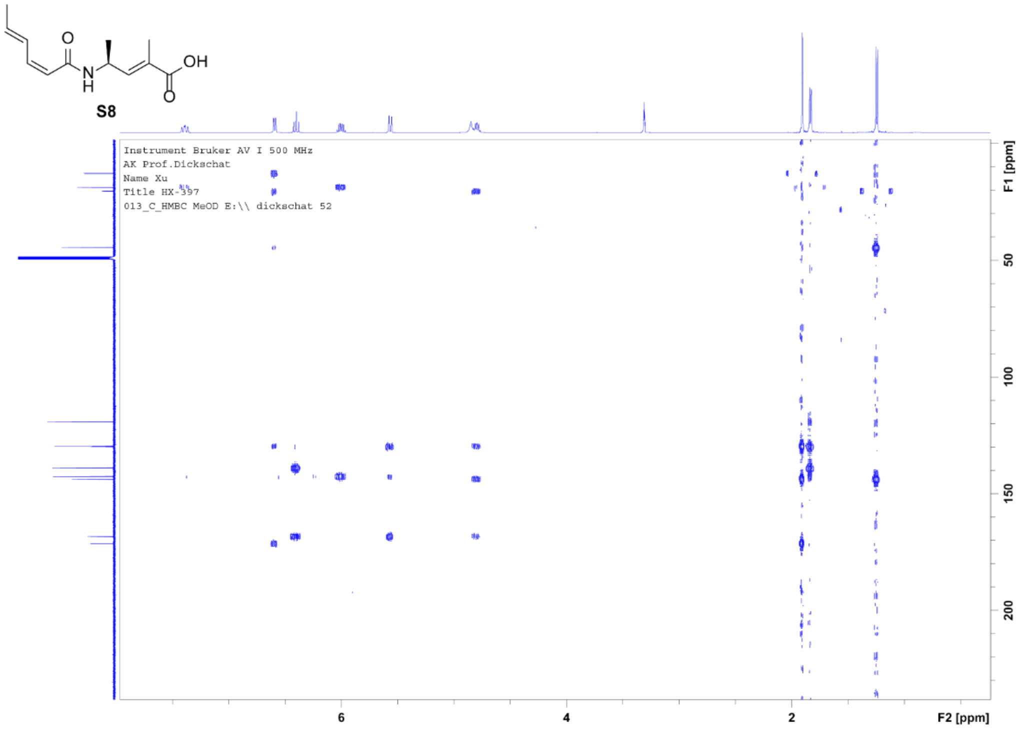

Figure S40. HMBC spectrum (CD_3OD) of **S8**.

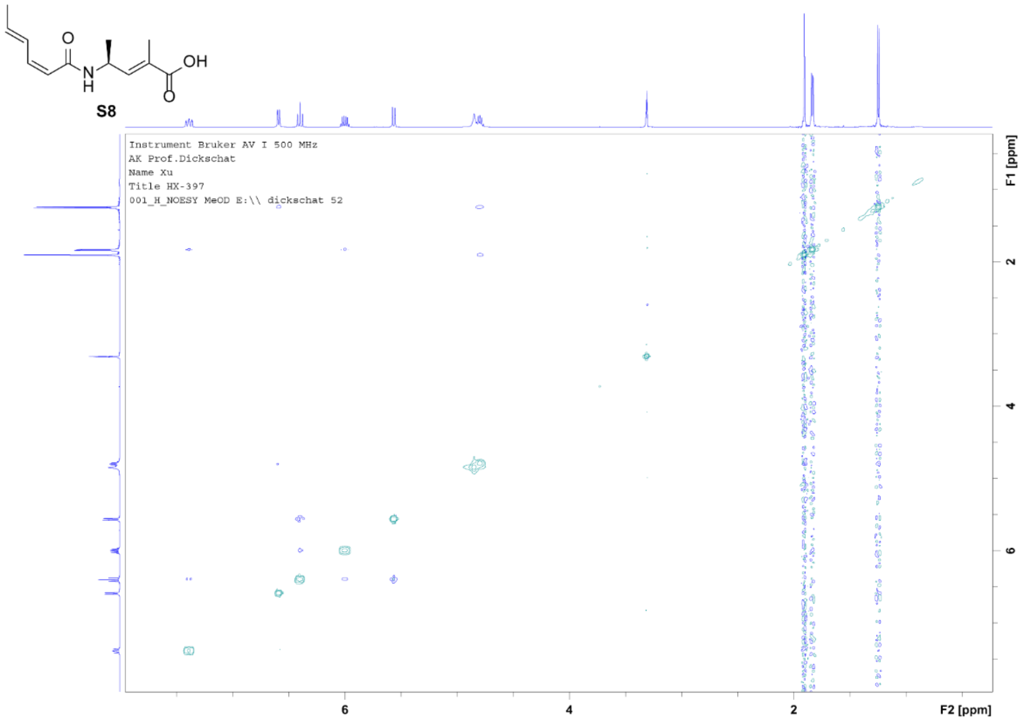

Figure S41. NOESY spectrum (CD_3OD) of **S8**.

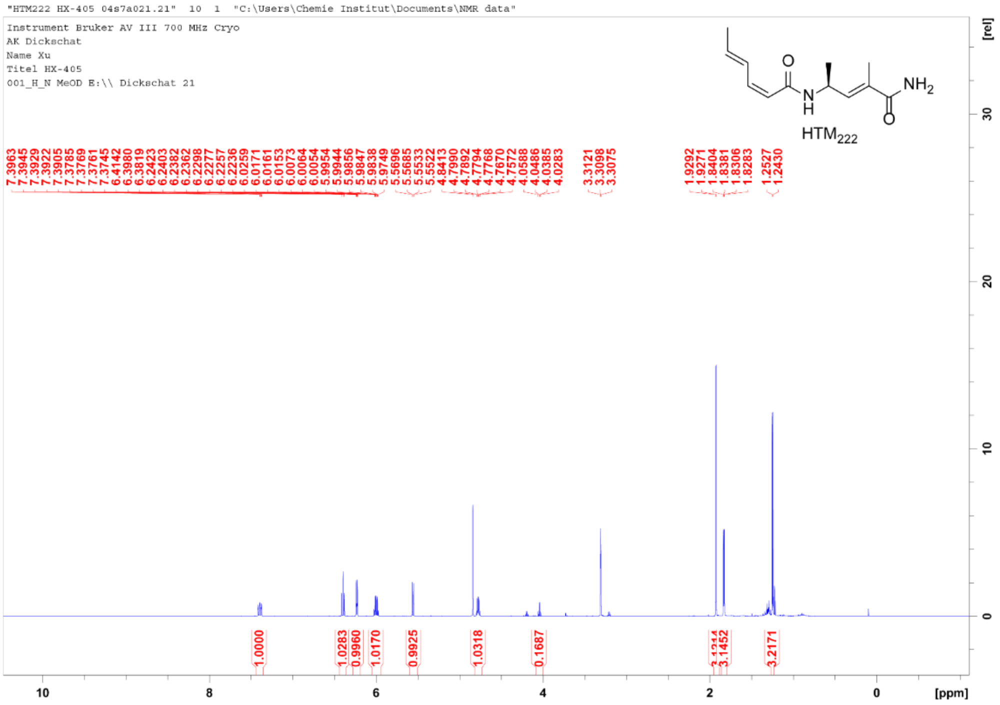

Figure S42. ^{1}H NMR spectrum (700 MHz, CD_3OD) of HTM_{222}.

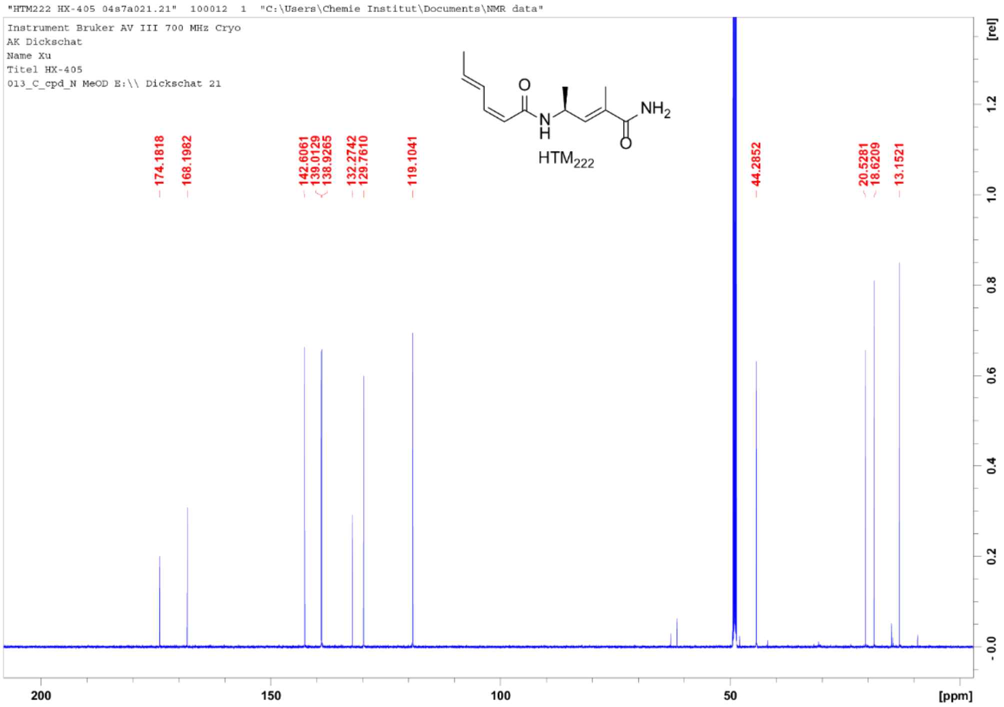

Figure S43. ^{13}C NMR spectrum (176 MHz, CD_3OD) of HTM_{222}.

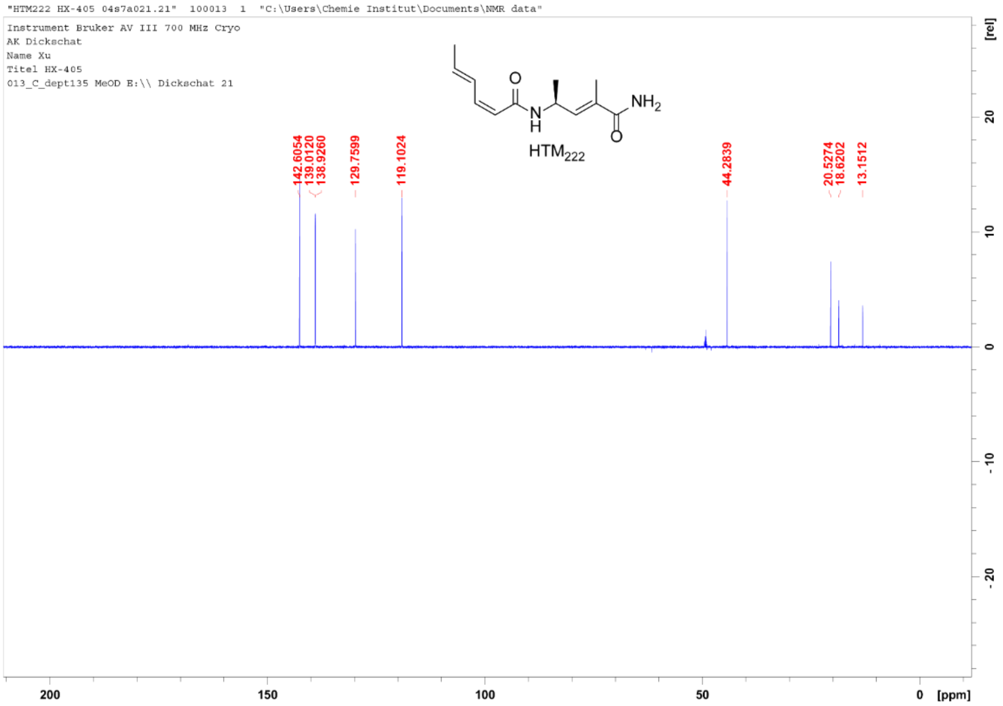

Figure S44. ^{13}C-DEPT-135 NMR spectrum (176 MHz, CD_3OD) of HTM_{222}.

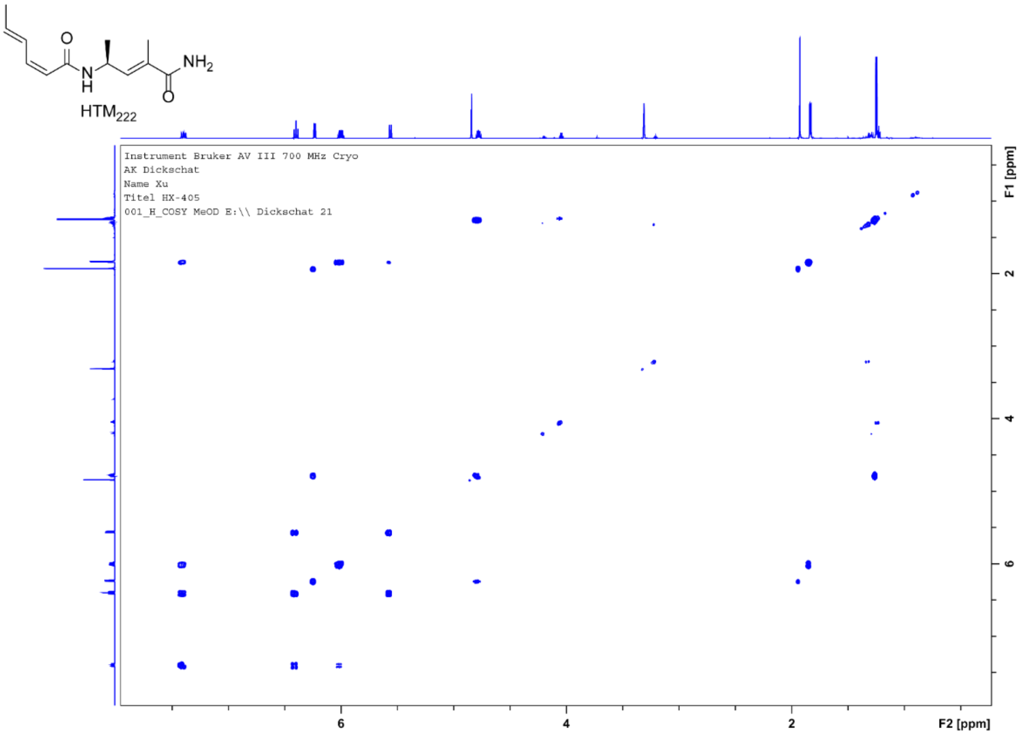

Figure S45. ^{1}H-^{1}H COSY spectrum (700 MHz, CD_3OD) of HTM_{222}.

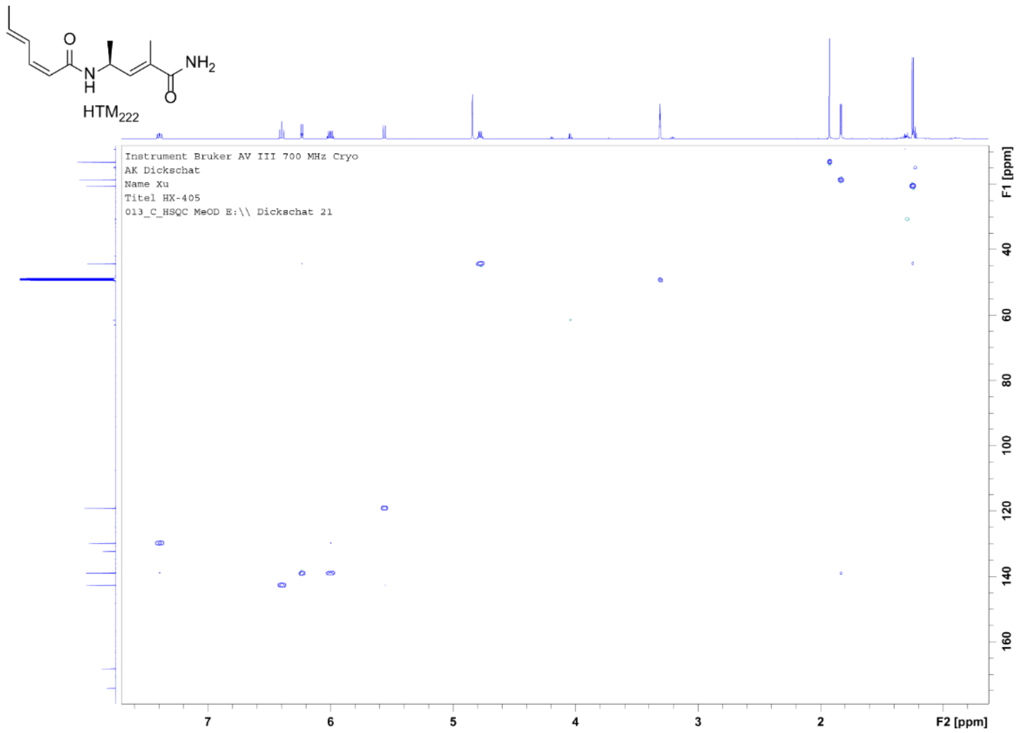

Figure S46. HSQC spectrum (CD_3OD) of HTM_{222}.

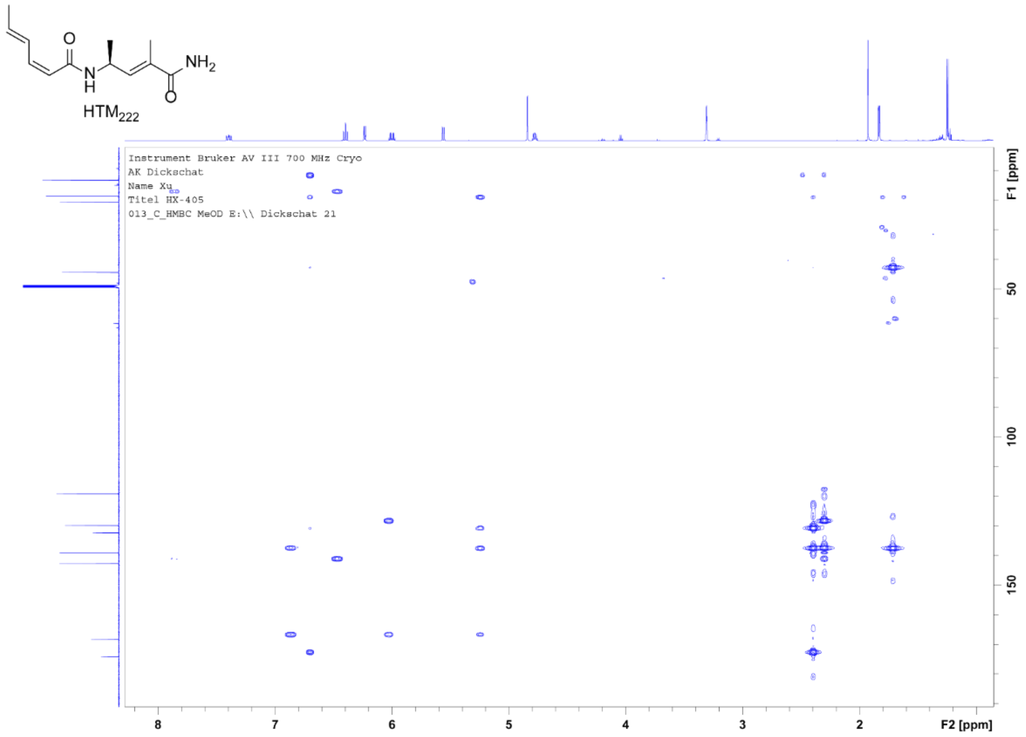

Figure S47. HMBC spectrum (CD_3OD) of HTM_{222}.

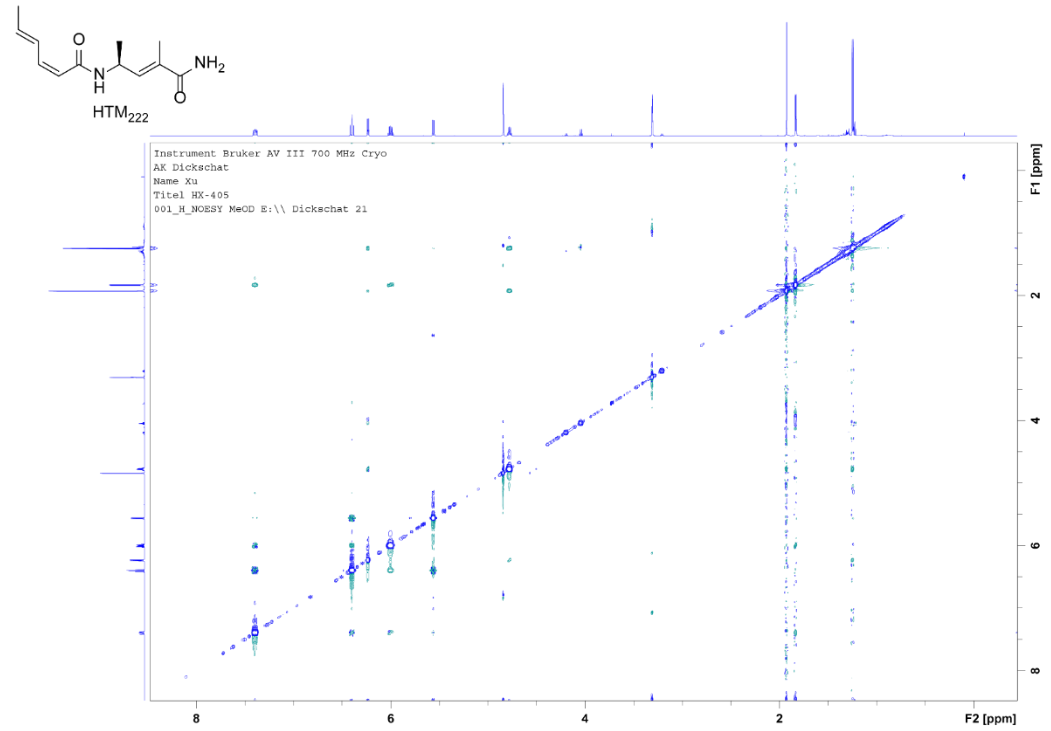

Figure S48. NOESY spectrum (CD_3OD) of HTM_{222}.

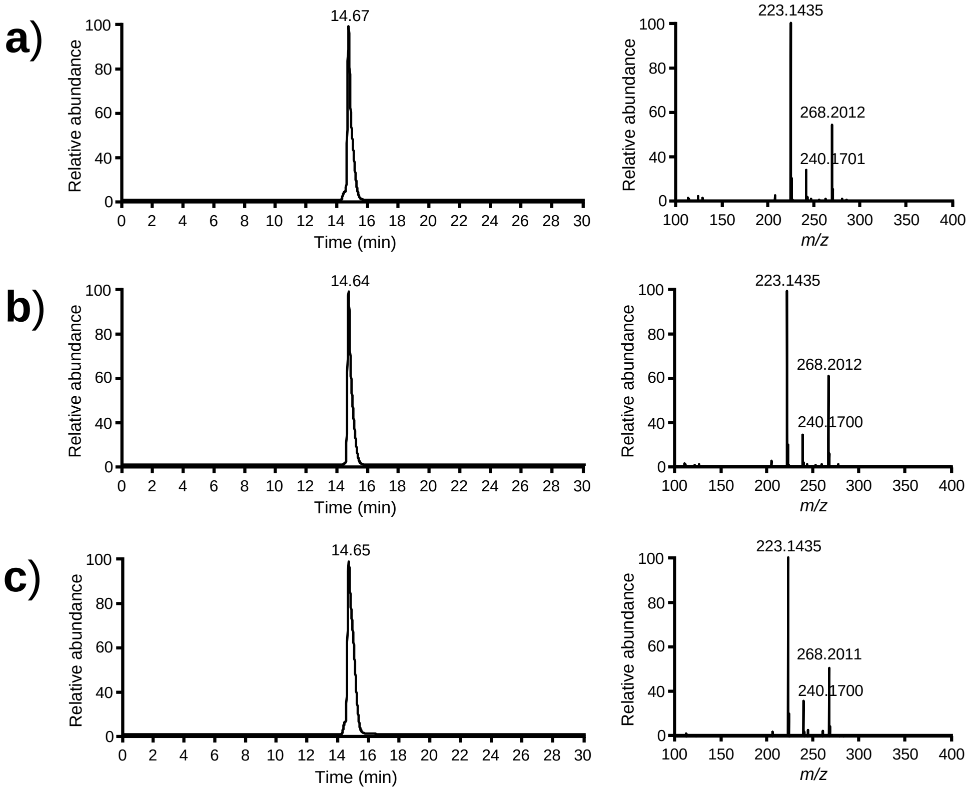

Figure S49. Identification of HTM_{222} by LC-ESI-HRMS. Chromatogram and ESI mass spectrum of a) natural HTM_{222} isolated from culture extracts, b) HTM_{222} synthesized from L-alanine (Scheme S1b), and c) coinjection of natural and synthetic HTM_{222}.

Table S4. Deduced functions of ORFs in putativr HTM biosynthetic gene cluster.

ORFs	Amino acid	Proposed function	Sequence similarity	Identity/Similarity
HtmA4	273	Reductase (KR)	WP_030668949.1	272/273
HtmI1	427	Isochorismate synthase	WP_030668946.1	391/393
HtmB3	552	AMP-dependent synthetase (A)	WP_051854476.1	550/552
HtmI2	210	Isochorismate synthase	WP_030668942.1	210/210
HtmU1	187	Unknown	WP_030668938.1	187/187
HtmU2	320	Unknown	WP_078868530.1	319/320
HtmT	418	Glycosyltransferase	WP_138966001.1	376/417
HtmR1	168	Transcriptional regulator	WP_030668931.1	155/155
HtmO	460	Amidase	WP_078868529.1	458/460
HtmA5	251	TEII	WP_037824320.1	248/251
HtmA1	4821	PKS	WP_158857389.1	4722/4816
		Loading module, GNAT-ACP		
		Module 1: KS-ATd-DH-KR-ACP		
		Module 2: KS-ATd-KR-ACP		
		Module 3: KS-DH-ACP		
		Module 4: KS-ATd-ACP		
HtmA2	7387	PKS/NRPS	WP_030668919.1	7345/7389
		Module 5: C-A-PCP		
		Module 6: KS-ATd-DH-KR-CMT-ACP		
		Module 7: KS-ATd-ACP		
		Module 8: C-A-PCP		
		Module 9: KS-ATd-KR-ACP		
		Module 10: KS-ATd-ACP-unknown domain)		
HtmA3	4748	PKS/NRPS	QDQ09714.1	4181/4781
		ACP_{A1}, ACP_{A2}, ACP_{A3}, Cd		
		Module 11: KS-ATd-KR-ACP		
		ACP		
		Module 12: KS-ATd-DH-ACP		
		Module 13: KS-ATd-KR-ACP		
		Module 14: KS-ATd-ACP		
HtmB1	2548	NRPS	WP_144001289.1	2416/2548
		Module 15: C-A-PCP		
		Module 16: C-A-OMT-PCP		
HtmB2	3091	NRPS	WP_144001288.1	2919/3099
		Module 17: C-A-PCP		
		Module 18: C-A-NMT-PCP-C_T		
HtmA8	108	ACP_d	WP_030670431.1	84/84
HtmA6	407	Ketosynthase (KS)	WP_030670435.1	406/407
HtmS	427	Hydroxymethylglutaryl-CoA synthase	WP_030670438.1	425/430
HtmH	786	Enoyl-CoA hydratase	WP_030670441.1	783/798
HtmA7	647	*trans*-AT_1; *trans*-AT_2	WP_078868641.1	642/644
HtmP	411	Cytochrome P450	WP_030670448.1	411/411
HtmU4	275	Unknown	WP_030670451.1	273/275
HtmU5	135	Unknown	WP_030670456.1	134/135
HtmR2	265	LysR family transcriptional regulator	WP_030670459.1	258/265
HtmK	609	Serine/threonine protein kinase	WP_051854586.1	585/587

GNAT, an adaptor domain for decarboxylation of malonyl-CoA to acetyl-CoA unit; KS, ketosynthase; AT, acyl transferase domain; ATd, AT docking domain domain; DH, dehydratase domain; KR, ketoreductase domain; CMT, *C*-methyltransferase domain; OMT, *O*-methyltransferase domain; NMT, *N*-methyltransferase domain; ACP, acyl carrier protein; TE, thioesterase; A, adenylation domain; C, condensation domain; Cd, C-terminal domain; C_T, terminal condensation domain; PCP, peptidyl carrier protein domain.

Gene deletion or site-directed mutation in vivo. The recombinant plasmids used for gene disruption and site-directed mutation were introduced into *S. spectabilis CCTCC M2017417* by conjugation using donor strain *E. coli* ET12567/pUZ8002 on ABB13 plates. After incubation at 28 °C for 12 h, the plate was overlaid with the final concentration of 35 µg/mL apramycin and 30 µg/mL nalidixic acid. Exconjugants were selected on ABB13 plates supplied with 35 µg/mL apramycin and 30 µg/mL nalidixic acid to check their antibiotic resistance. Then single colonies were patched onto ABB13 plates containing 35 µg/mL apramycin and onto ABB13 plates without antibiotic, respectively, to screen for the double crossover mutant. The mutant candidates with correct phenotype (Apr^R) were further verified by PCR and sequencing with corresponding primers (Table S2).

Construction of gene deletion plasmids. To knock out the *htm* gene cluster, two homologous recombination fragments of 2061 bp and 2060 bp flanking the ~92 kb *htm* cluster in the genome were amplified by PCR using two pairs of primers htm-L-up and htm-L-re, htm-R-up and htm-R-re, respectively (Table S2). These two fragments were then cloned into the *Streptomyces-E. coli* shuttle vectors pYH7[4] treated with *Nde*I and *Hin*dIII by Gibson method to create the recombinant plasmid pWHU5001. To verify the plasmid and the mutant, a pair of primers htm-confirm-up and htm-confirm-re (Table S2) flanking the deletion region were used for PCR and sequencing.

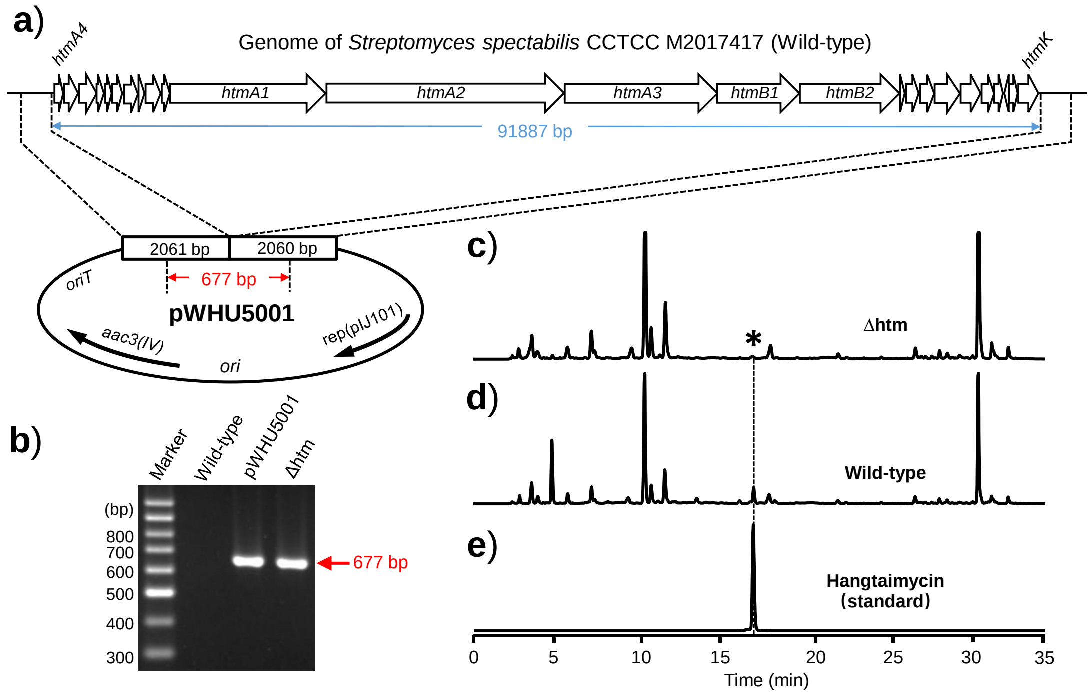

Figure S50. Deletion of the entire HTM biosynthetic gene cluster and its verification. a) Gene cluster deletion mediated by double homologous recombination in *Streptomyces spectabilis* CCTCC M2017417. b) Confirmation of the mutant by PCR (red arrows indicate the size of the PCR product expected for the Δhtm mutant). c–e) HTM production detected by HPLC (asterisk indicates absence of HTM). The plasmid pWHU5001 and wild-type genomic DNA were used as positive and negative control, respectively.

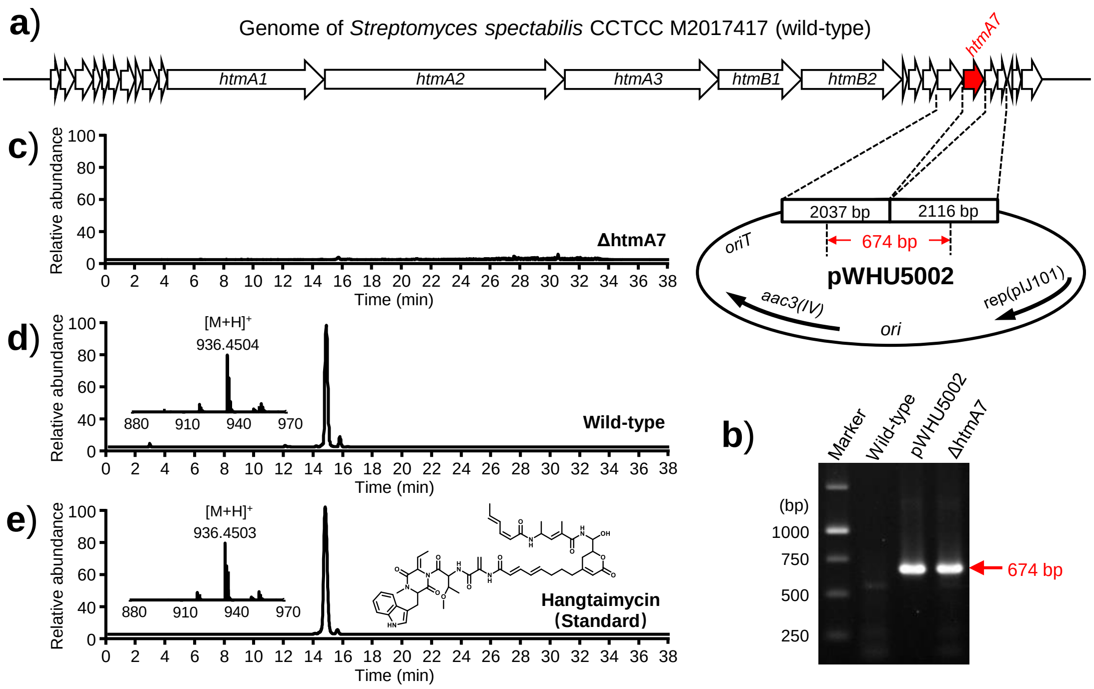

Figure S51. Scheme presentation of *htmA7* deletion and verification. a) In-frame deletion of *htmA7* mediated by double homologous recombination in vivo. b) The mutant is confirmed by PCR amplification and sequencing using the genomic DNA from the mutant, wild-type strain (negative control) and plasmid pWHU5002 (positive control) as the template. c–e) HTM production was detected by LC-ESI-HRMS analysis. The size of predicted and observed PCR fragments are shown by red solid arrows.

Construction of site-directed mutation plasmids. To construct site-directed mutation plasmids of KR_1, DH_1, KR_2, ACP_2, KS_3, DH_3 and ACP_3, each two homologous recombination fragments were amplified by overlapping PCR using primer pairs listed in Table S2, then fused into the shuttle vector pYH7 digested with *Nde*I *and Hin*dIII to yield the corresponding recombinant plasmids (Table S1).

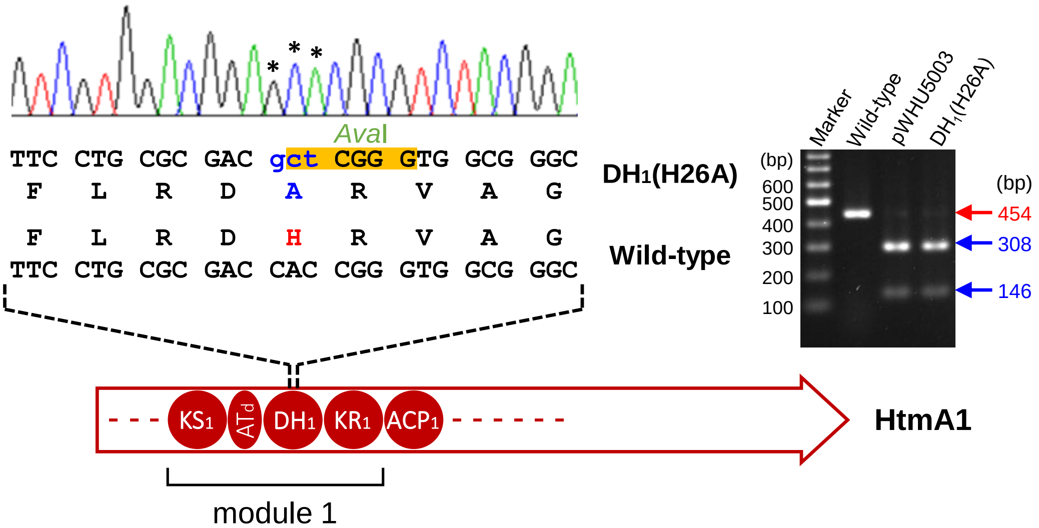

Figure S52. Site-directed mutation of DH_1 in module 1 of HtmA1. The histidine (H) in active motif H×××G××××P was mutated to alanine (A). The changed nucleic acids and corresponding amino acid are shown in blue and marked with asterisks. An *Ava*I restriction site which is used for mutant candidate screening by PCR is highlighted in yellow. The PCR product of mutation was confirmed by restriction enzyme digestion and sequencing. The plasmid pWHU5003 and wild-type genomic DNA were used as positive and negative control, respectively.

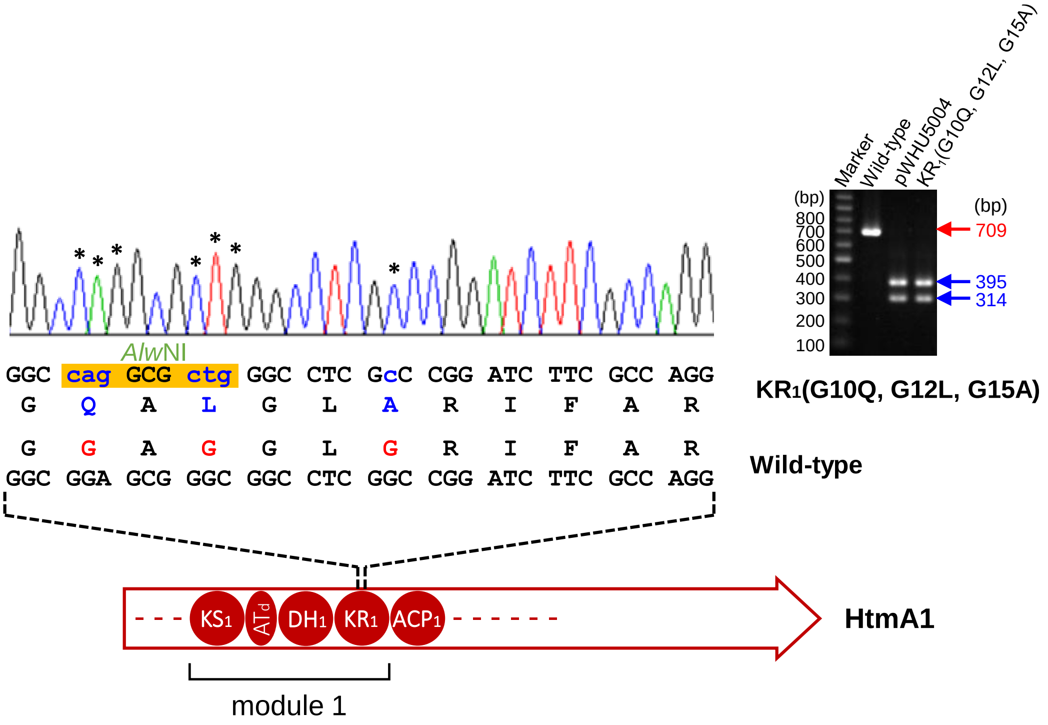

Figure S53. Site-directed mutation of KR_1 in module 1 of HtmA1. Three glysines (G) in NADPH binding site of G×G××G××××A were mutated to glutamine (Q), leucine (L) and alanine (A), respectively. The changed nucleic acids and corresponding amino acids are shown in blue and marked with asterisks. An *Alw*NI restriction site which is used for mutant candidate screening by PCR is highlighted in yellow. The PCR product of mutation was confirmed by restriction enzyme digestion and sequencing. The plasmid pWHU5004 and wild-type genomic DNA were used as positive and negative control, respectively.

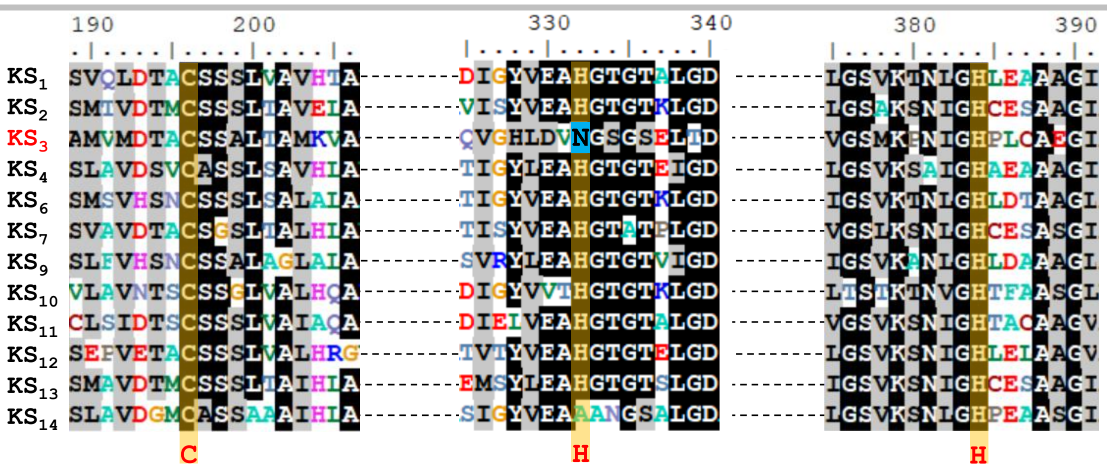

Figure S54. The partial sequence alignment of KS in HTM PKS. The active site triad that is required for Claisen condensation activity are highlighted as C-H-H. A native variation (N_{332}) in the catalytic trail of KS_3 is indicated against blue background.

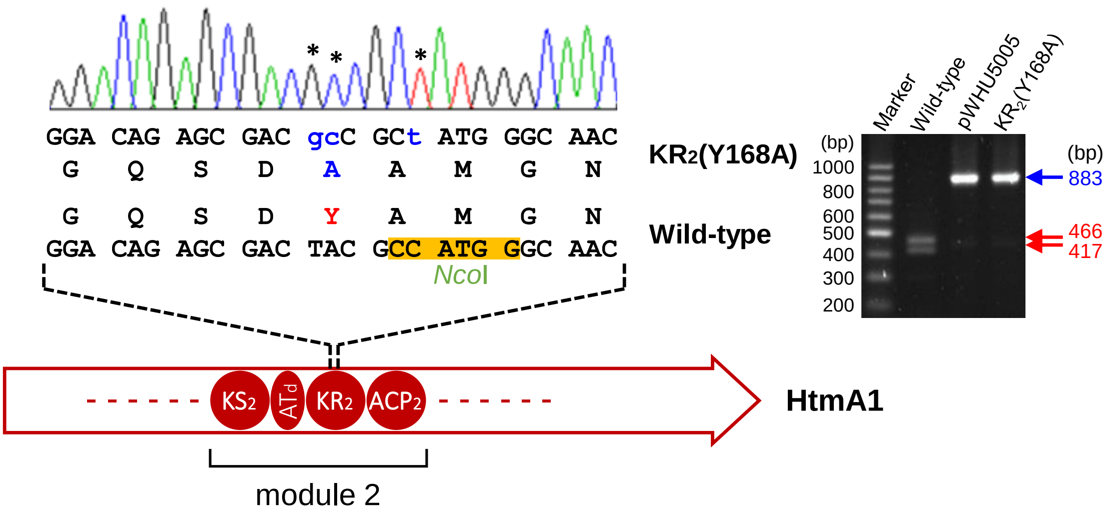

Figure S55. Site-directed mutation of KR_2 in module 2 of HtmA1. The tyrosine (Y) in active motif was mutated to alanine (A). The changed nucleic acids and corresponding amino acid are shown in blue and marked with asterisks. A *Nco*I restriction site which is destroyed in mutant for candidate screening by PCR is highlighted in yellow. The PCR product of mutation was confirmed by restriction enzyme digestion and sequencing. The plasmid pWHU5005 and wild-type genomic DNA were used as positive and negative control, respectively.

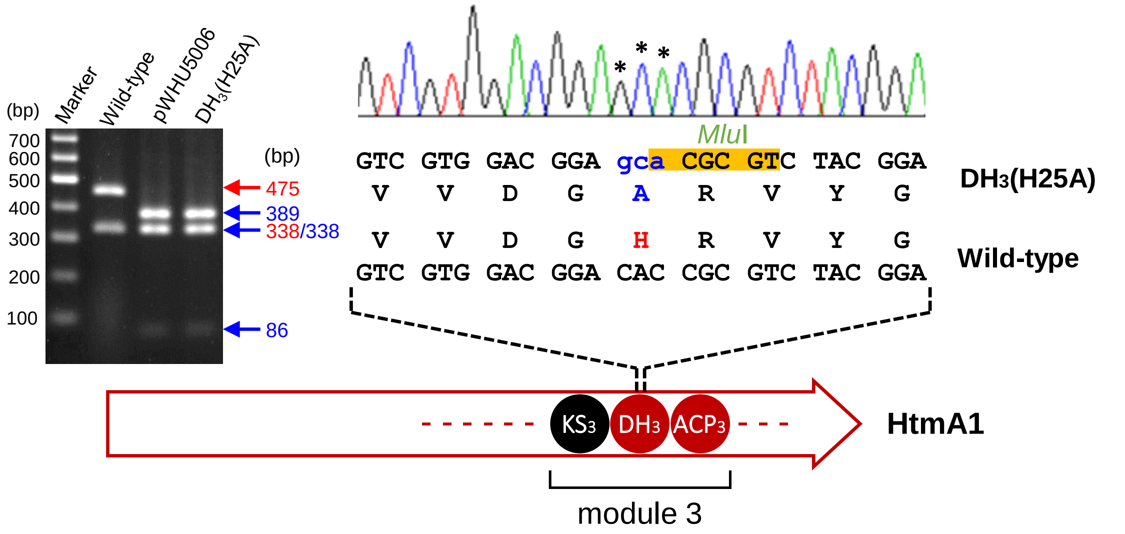

Figure S56. Site-directed mutation of DH_3 in module 3 of HtmA1. The histidine (H) in active motif H×××G××××P was mutated to alanine (A). The changed nucleic acids and corresponding amino acid are shown in blue and marked with asterisks. A *Mlu*I restriction site which is used for mutant candidate screening by PCR is highlighted in yellow. The PCR product of mutation was confirmed by restriction enzyme digestion and sequencing. The plasmid pWHU5006 and wild-type genomic DNA were used as positive and negative control, respectively.

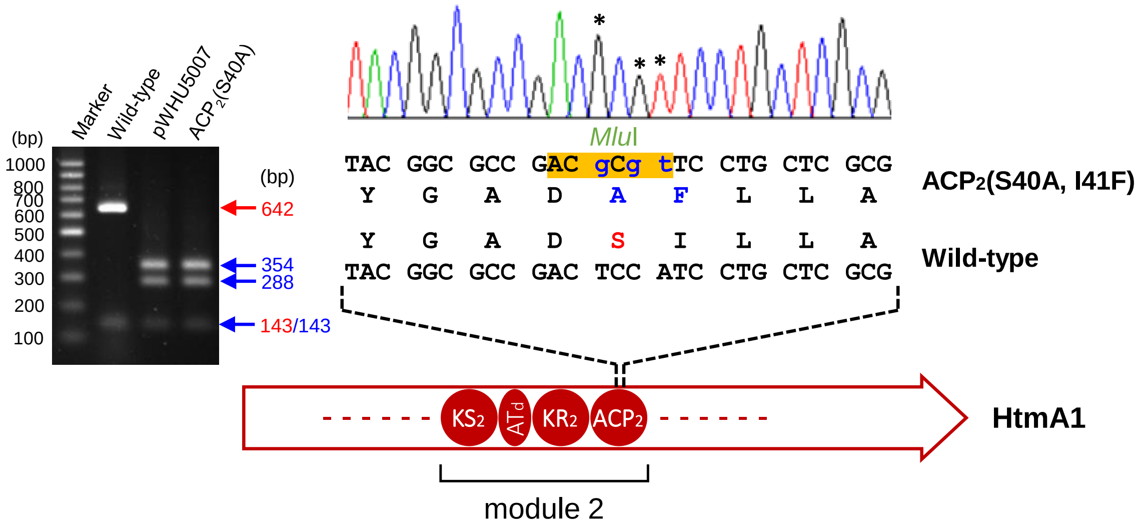

Figure S57. Site-directed mutation of ACP_2 in module 2 of HtmA1. The active site of ACP2 was mutated from serine (S) to alanine (A). The changed nucleic acids and corresponding amino acid are shown in blue and marked with asterisks. A *Mlu*I restriction site which is used for mutant candidate screening by PCR is highlighted in yellow. The PCR product of mutation was confirmed by restriction enzyme digestion and sequencing. The plasmid pWHU5007 and wild-type genomic DNA were used as positive and negative control, respectively.

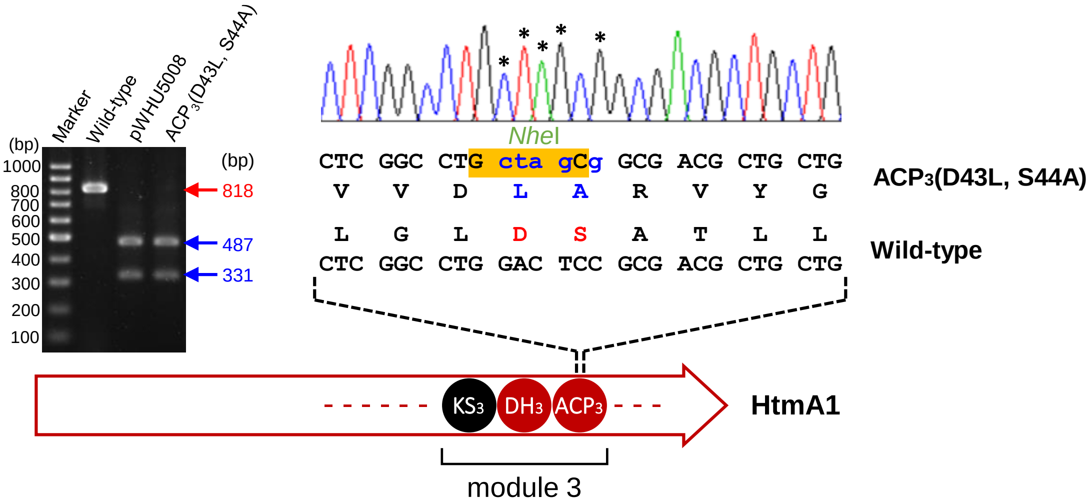

Figure S58. Site-directed mutation of ACP_3 in module 3 of HtmA1. The active site of ACP3 was mutated from serine (S) to alanine (A) coupling with another mutation from aspartic acid (D) to leucine (L). The changed nucleic acids and corresponding amino acids are shown in blue and marked with asterisks. A *Nhe*I restriction site which is used for mutant candidate screening by PCR is highlighted in yellow. The PCR product of mutation was confirmed by restriction enzyme digestion and sequencing. The plasmid pWHU5008 and wild-type genomic DNA were used as positive and negative control, respectively.

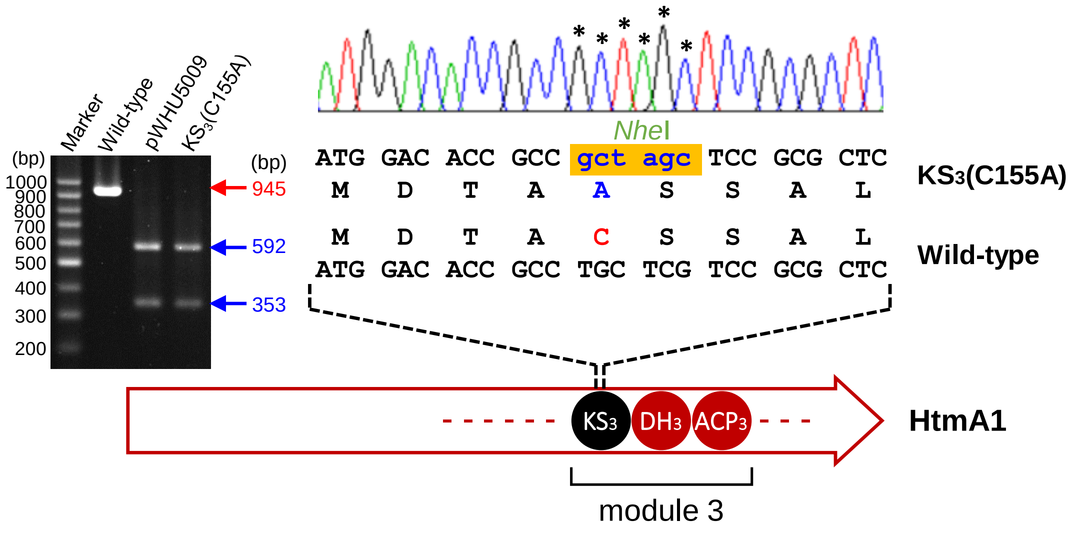

Figure S59. Site-directed mutation of KS_3 in module 3 of HtmA1. The cysteine (C) in substrate binding site was mutated to alanine (A). The changed nucleic acids and corresponding amino acid are shown in blue and marked with asterisks. A *Nhe*I restriction site which is used for mutant candidate screening by PCR is highlighted in yellow. The PCR product of mutation was confirmed by restriction enzyme digestion and sequencing. The plasmid pWHU5009 and wild-type genomic DNA were used as positive and negative control, respectively.

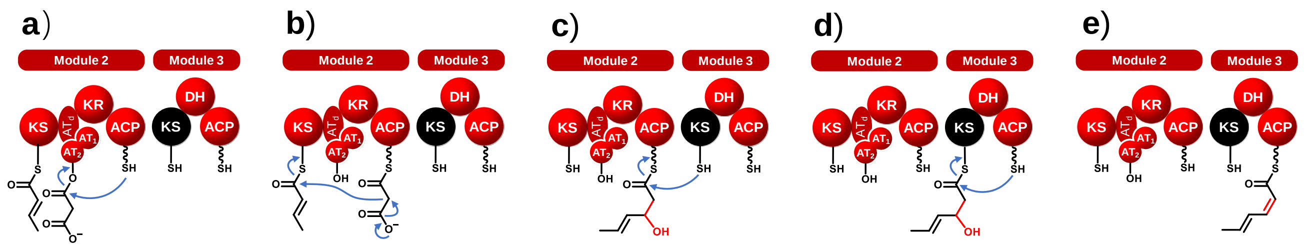

Figure S60. Proposed mechanism of the dehydrating bimodule (modules 2 and 3) of HtmA1. a, b) Elongation to the triketide by module 2 with malonyl-CoA, c) ketoreduction by KR_2 and transfer to KS_3 that only acts as transacylase (non-elongating), d, e) transfer to ACP_3 and dehydration.

Construction of protein expression plasmids. The expression plasmids for module 1, module 2, module 3 and module 3 containing the mutated KS_3 domain were generated by PCR amplifying with primes listed in Table S2, then inserted it into vector pET28a(+) to yield the corresponding recombinant plasmids (Table S1).

Expression and purification of proteins. For expression of *holo*-module 2, *holo*-module 3, *holo*-module 3(KS_3(C155A)), the host strain was *E. coli* BAP1.[2] For HtmA7, the strain was *E. coli BL21(DE3) with* plasmid pGro7.[9] For *holo*-module 1, the host strain was *E. coli* BAP1 with pGro7. The overnight seed culture with kanamycin (25 μg/mL) and chloramphenicol (25 μg/mL) for pGro7 grown in 2×TY medium were inoculated into LB medium (1 L) and continue to grow at 37 °C to A600 values of 0.3-0.5. For HtmA7 and *holo*-module 1 expression, additionally supplemented with 3.3 mM arabinose to induce GroEL/ES chaperone in pGro7.[9] After this, 0.2 mM of isopropyl-β-dthiogalactopyranoside (IPTG) was added and further incubate at 16 °C for 18 h. Then, the culture were harvested by centrifugation (4500×*g*) for 15 min and suspended in lysis buffer (20 mM Tris-HCl, pH 7.5, containing 300 mM NaCl and 10% glycerol) and then lysed by sonication. The lysates cleared by centrifugation (17000×*g*) for 60 min were added to the His-Bind resin column (Sigma-Aldrich). The resin absorbed with proteins were first washed with three column volumes of lysis buffer, then eluted with a step gradient lysis buffer contained increasing imidazole concentrations of 15, 50, 100, 200 and 400 mM. Fractions containing targeted proteins were further purified on a Superdex 200 16/600 column with lysis buffer at a flow rate of 1 mL/min. The fractions containing purified protein were pooled and concentrated based on SDS-PAGE (Figure S61). The theoretical molecular weights of target proteins are 160050 Da (*holo*-module 1), 136335 Da (*holo*-module 2), 90462 Da (*holo*-module 3), 90430 Da (*holo*-module 3(KS_3(C155A)), 72873 Da (HtmA7), respectively.

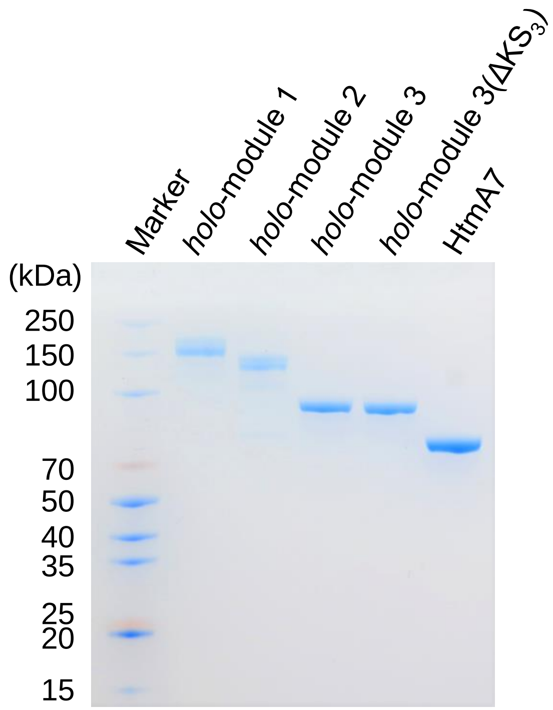

Figure S61. SDS-PAGE analysis of all recombinant enzymes used in this study. The theoretical molecular weights of target proteins are 160050 Da (*holo*-module 1), 136335 Da (*holo*-module 2), 90462 Da (*holo*-module 3), 90430 Da (*holo*-module 3(ΔKS_3)), 72873 Da (HtmA7), respectively.

Module assay. For the polyketide chain elongation assay, reaction was performed in a total volume of 100 µl containing 10 µM *holo*-module 1/*holo*-module 2/*holo*-module 3/*holo*-module 3(ΔKS_3), 200 µM (*E*)-2-butenoyl-SNAC, 5 µM HtmA7, 200 µM malonyl-CoA, 5 mM ATP, 100 µM NADPH and 10 mM $MgCl_2$ in 50 mM Tris-HCl buffer (pH 7.5) at 25 °C for 3 h. After reaction, the solution was hydrolyzed to release polyketide chains as described below, then the products were submitted to LC-ESI-HRMS analysis.

Hydrolysis of thioester-bound products and LC-ESI-HRMS analysis. After enzymatic assay, the reaction was quenched by the addition of 20% trichloroacetic acid (TCA). The precipitated protein was pelleted by centrifugation and was washed twice with 10% TCA. The protein pellet was then dissolved in 100 µl KOH (0.1 M) for 2 h at room temperature. 5 µl trifluoroacetic acid (TFA, 50%) was then added, and the solution was centrifuged to remove precipitated proteins. The solution was finally applied to LCESI-HRMS for analysis. LC-ESI-HRMS analysis was carried out on a Thermo Electron LTQ-Orbitrap XL using negative mode electrospray ionization, coupled to Thermo Accela 600 fitted with a Phenomenex Luna C18 column (250×4.6 mm, 5 µm) at a flow rate of 0.8 mL/min. The gradient elution system for separation of protein is a mixture of solvent A (H_2O) and solvent B (acetonitrile). The procedure of the elution system is as following: 0-10 min, 2-25% B; 10-20 min, 25-75% B; 20-25 min, 100% B; 25-30 min, 2% B. The mass spectrometer was set to full scan (from *m*/*z* 60 to 500).

Synthesis of (*E*)-2-butenoyl-SNAC (2). Crotonic acid (430 mg) was dissolved in CH_2Cl_2 (15 mL) on 0 °C for 15 minutes. Then, added DMAP (122 mg), EDC·HCl (230 mg) and SNAC (500 mg) to this solution. The mixture was stirred overnight at room temperature. Saturated aqueous NH_4Cl was added to quench this reaction. After this, the mixture was extracted three times with CH_2Cl_2, and concentrated under reduced pressure. The final pure (*E*)-2-butenoyl-SNAC (**2**) was purified by HPLC with a yield of 51%. DMAP: 4-(*N*,*N*-dimethylamino) pyridine, SNAC: 2-(acetylamino)ethanethiol, EDC·HCl: *N*-ethyl-*N'*-(3-dimethylaminopropyl)carbodiimide hydrochloride. ^{1}H NMR (400 MHz, CD_3OD): δ_H 6.91 (dq, *J* = 15.3 Hz, 6.9 Hz, 1H), 6.17 (dd, *J* = 15.4 Hz, 1.4 Hz, 1H), 3.31 (t, *J* = 6.7 Hz, 2H), 3.08 (t, *J* = 6.7 Hz, 2H), 1.90 (s, 3H), 1.89 (dd, *J* = 6.9 Hz, 1.4 Hz, 3H); ^{13}C NMR (100 MHz, CD_3OD): δ_C 190.8, 173.5, 142.9, 131.0, 40.3, 28.9, 22.7, 18.2 (Figures S62 and S63). ESI-HRMS (*m/z*): $[M+H]^+$ calculated for $C_8H_{14}NO_2S^+$, 188.0740; found, 188.0732 (Figure S64).

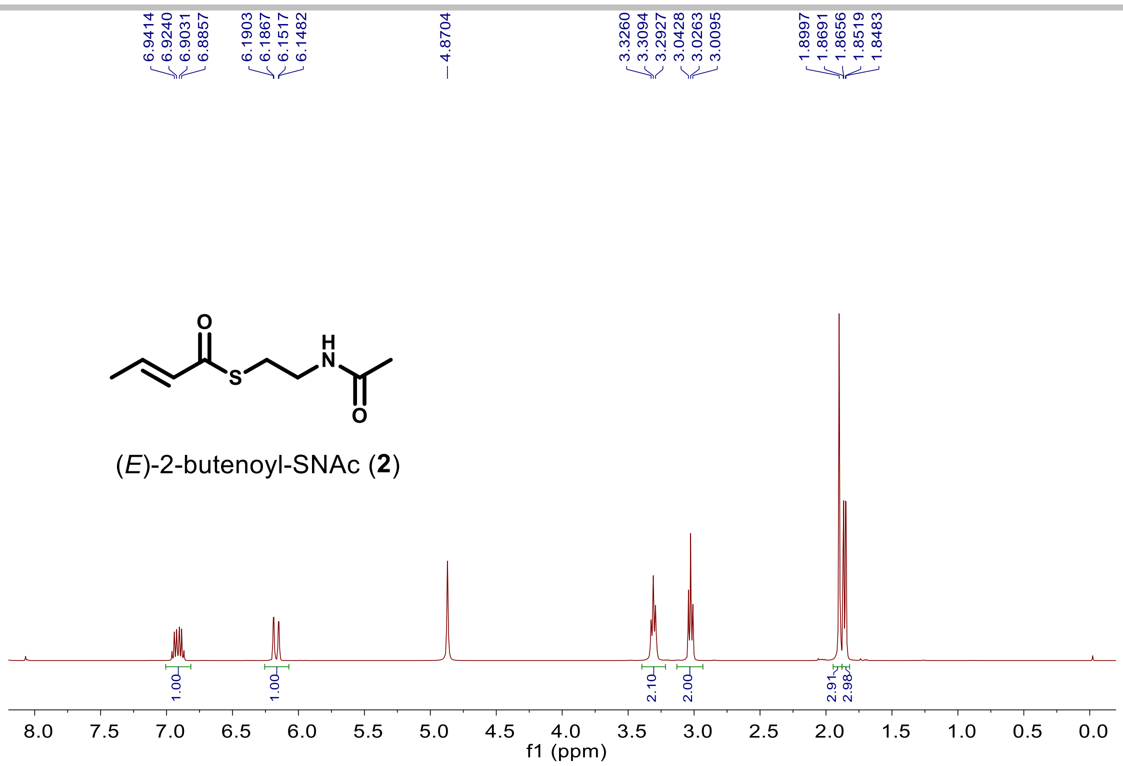

Figure S62. ^{1}H NMR spectrum of compound **2** (400 MHz, $(CD_3)_2SO$).

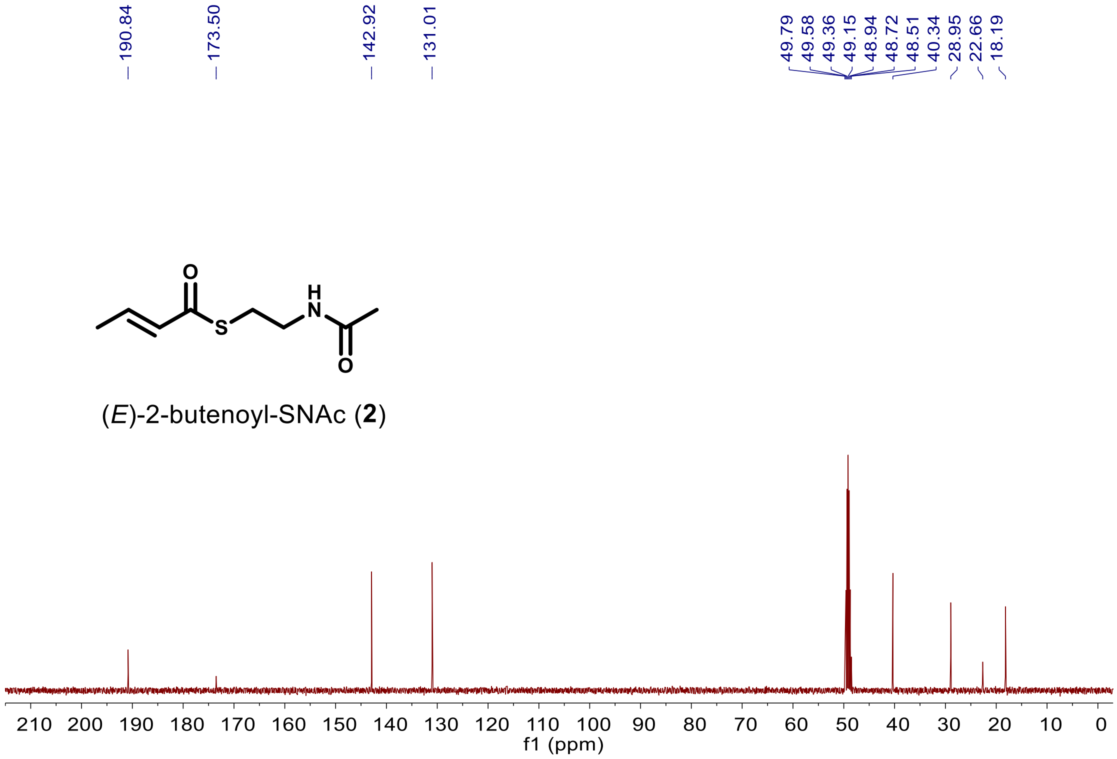

Figure S63. ^{13}C NMR spectrum of compound **2** (150 MHz, $(CD_3)_2SO$).

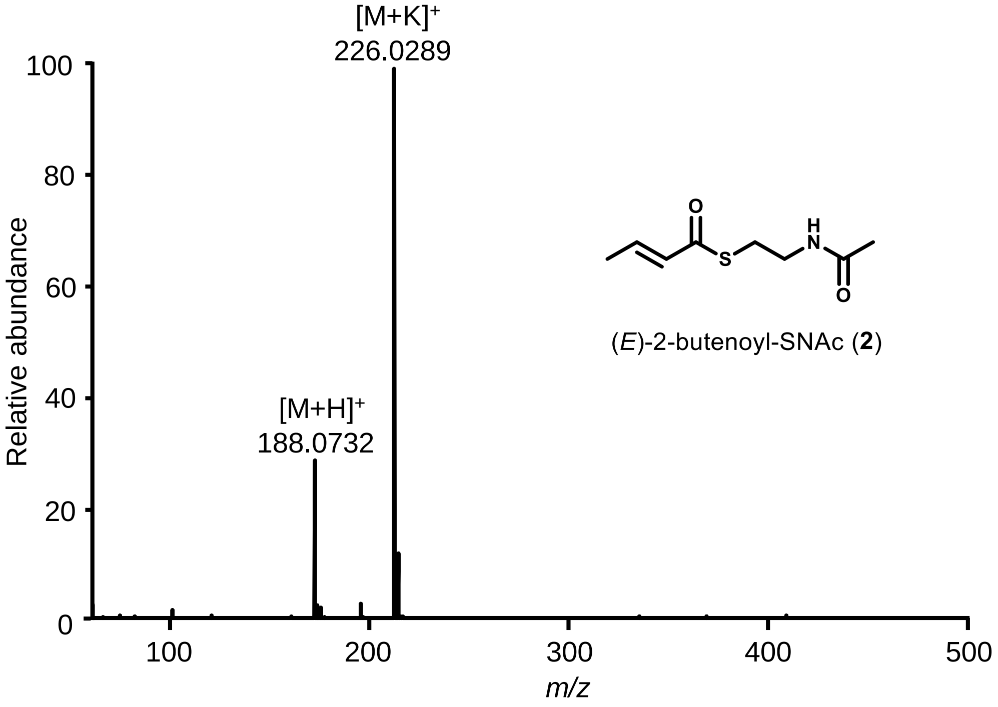

Figure S64. ESI-HRMS of compound **2**.

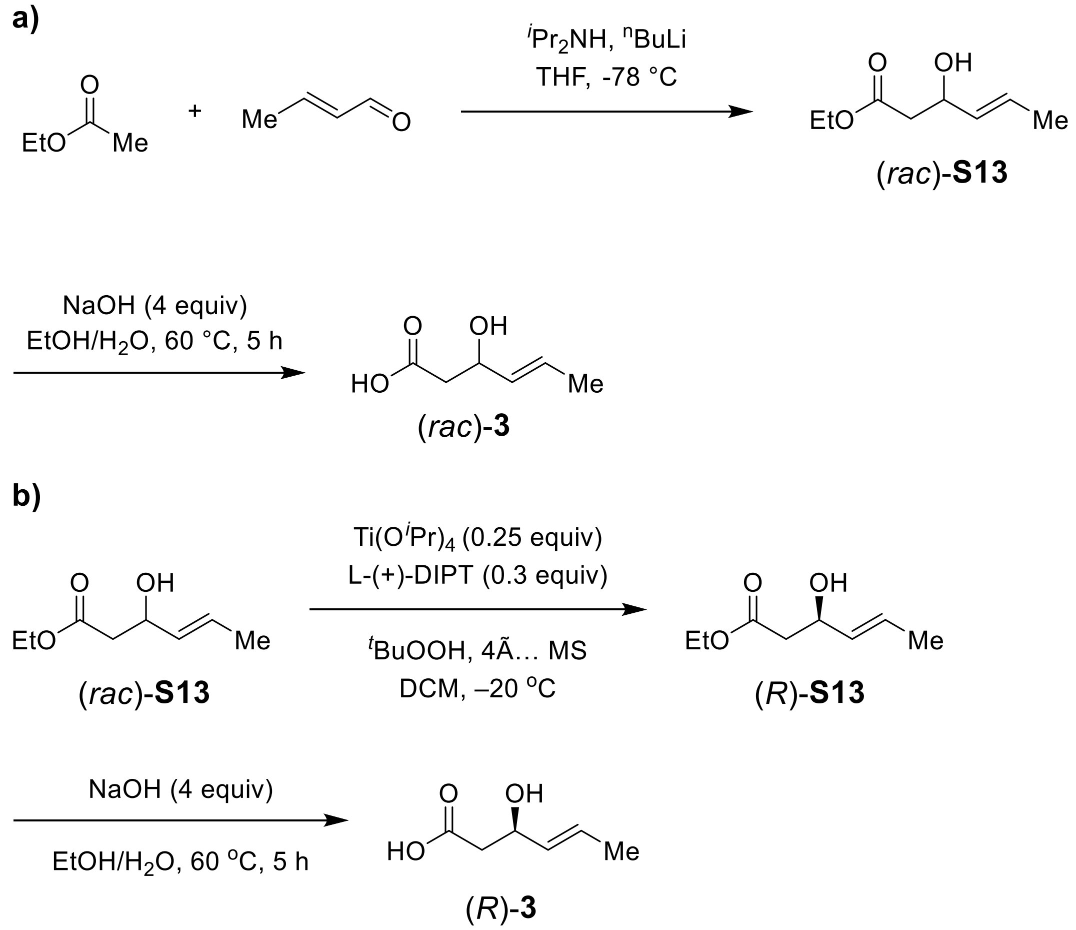

Scheme S2. Preparation of a) (*rac*)-**3** and b) enantiomerically enriched (*R*)-**3** by kinetic resolution through Sharpless epoxidation of ethyl (*rac*)-3-hydroxyhex-4-enoate (**S13**) and saponification. The *S* enantiomer is epoxidised faster than the *R* enantiomer, leading to its enrichment.

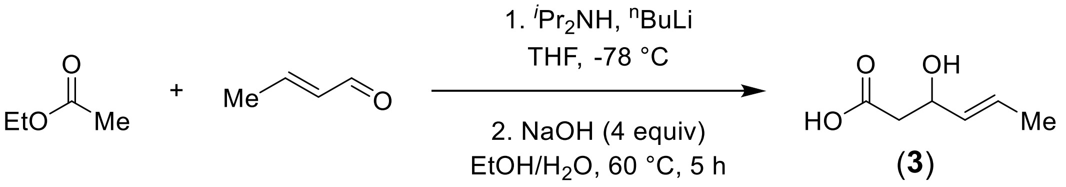

Synthesis of (*E*)-3-hydroxyhex-4-enoic acid (3) To a solution of ^{i}Pr$_2$NH (3.5 mL, 28 mmol) in THF (10 mL) was added ^{n}BuLi (10 mL, 2.5 M in hexane, 25 mmol) dropwise at -78 °C under N_2. EtOAc (2 mL, 24 mmol) was then added dropwise. The resulting mixture was allowed to stir at -78 °C for 40 min, and then a solution of crotonaldehyde (1.6 mL, 20 mmol) in THF (2 mL) was added dropwise. After 1 h at -78 °C, the solution was poured into an ice-cold mixture of a saturated solution of NH_4Cl and EtOAc. The resulting mixture was stirred vigorously for a few minutes, and the layers were separated. The aqueous layer was extracted with EtOAc twice. The combined organic layers were dried over $MgSO_4$, filtered and concentrated to afford the ethyl (*E*)-3-hydroxyhex-4-enoate (*rac*)-**S13** as a colorless oil. To the residue was added NaOH (3.2 g, 80 mmol), EtOH (14 mL) and H_2O (4 mL). The resulting mixture was allowed to stir at 60 °C for 5 h. The solvent was removed under reduced pressure. The residue was dissolved in H_2O, and then the aqueous solution was washed with Et_2O. The aqueous solution was acidified to pH 1 with an aqueous solution of HCl (2 M) and extracted with EtOAc. The combined extracts were washed with brine, dried over Na_2SO_4, filtered and concentrated. Purification by column chromatography (5% MeOH in DCM) gave (*E*)-3-hydroxyhex-4-enoic acid (**3**)[10] as a yellow solid (1.53 g, 67 % yield over 2 steps). ^{1}H NMR (400 MHz, CD_3OD): δ 5.71 (dq, J = 15.2, 6.4 Hz, 1H), 5.51 (dd, J = 15.2, 6.4 Hz, 1H), 4.42 (q, J = 6.8 Hz, 1H), 2.45 (dd, J = 15.1, 6.8 Hz, 1H),

2.43 (dd, J = 15.1, 6.8 Hz, 1H), 1.66 (d, J = 6.8 Hz, 3H). ^{13}C NMR (100 MHz, CD_3OD): δ 175.0, 134.0, 127.6, 70.2, 43.3, 17.8 (Figures S65 and S66). ESI-HRMS (*m*/*z*): $[M+H]^+$ calculated for $C_6H_9O_3^+$, 129.0546; found, 129.0558 (Figure S67).

Synthesis of (3*R*,4*E*)-3-hydroxyhex-4-enoic acid (*R*)-S13.[10] To a mixture of 4 Å molecular sieves (0.15 g) and $Ti(O^iPr)_4$ (0.25 g, 1 mmol) in CH_2Cl_2 (8 mL) was added L-(+)-DIPT (0.25 mL, 1.2 mmol) dropwise at –20 °C. The mixture was stirred at –20 °C for 30 min, and a solution of (*rac*)-ethyl (4*E*)-3-hydroxy-4-hexenoate (**S13**, 0.63 g, 4 mmol) in CH_2Cl_2 (2 mL) was injected. The mixture was stirred for additional 30 min at –20 °C, and then cooled to –40 °C. A solution of ^{t}BuOOH in decane (0.8 mL, 5 M, 4 mmol) was added dropwise. The reaction was carried out at –20 °C for 30 h, and quenched by addition of Me_2S (0.29 mL, 4 mmol). After 30 min at –20 °C, 10% tartaric acid (2 mL), KF (0.29 g, 5 mmol), and Celite (0.25 g) were added. The resulting mixture was stirred at room temperature for 30 min and filtered through a pad of Celite with EtOAc. The residue was purified by column chromatography (10% EtOAc in hexanes) to produce ethyl (3*R*,4*E*)-3-hydroxy-4-hexenoate (*R*)-**S13** as a colorless oil (276.5 mg, 43% yield): $[\alpha]_D^{20}$ = +4.9 (*c* = 0.208, $CHCl_3$). The enantiomeric excess of ethyl (3*R*,4*E*)-3-hydroxy-4-hexenoate was determined by HPLC analysis on chiral column (Daicel, Chiralcel AD-H). Conditions: hexane/isopropanol = 98/2, flow rate = 0.5 mL/min, UV-Vis detection at λ_{max} = 214 nm, t_R = 31.6 min (major), 33.4 min (minor).

Synthesis of (3*R*,4*E*)-3-hydroxyhex-4-enoic acid. A 5 mL flask was charged with ethyl (3*R*,4*E*)-3-hydroxy-4-hexenoate ((*R*)-**S13**, 114.3 mg, 0.63 mmol), NaOH (106.4 mg, 4 mmol), EtOH (0.7 mL) and H_2O (0.2 mL). The resulting mixture was allowed to stir at 60 °C for 5 h. The solvent was removed under reduced pressure. The residue was dissolved in H_2O, and then the aqueous solution was washed with Et_2O. The aqueous solution was acidified to pH = 1 with an aqueous solution of HCl (2 M) and extracted with EtOAc. The combined extracts were washed with brine, dried over Na_2SO_4, filtered and concentrated. Purification by column chromatography (5% MeOH in DCM) gave (3*R*,4*E*)-3-hydroxyhex-4-enoic acid (*R*)-**3** as a yellow oil (46.8 mg, 57 % yield). ^{1}H NMR (400 MHz, CD_3OD): δ 5.71 (dq, J = 15.2, 6.4 Hz, 1H), 5.51 (dd, J = 15.2, 6.4 Hz, 1H), 4.42 (q, J = 6.8 Hz, 1H), 2.45 (d, J = 4.0 Hz, 1H), 2.43 (d, J = 3.6 Hz, 1H), 1.66 (d, J = 6.8 Hz, 3H). ^{13}C NMR (100 MHz, CD_3OD): δ 175.0, 134.0, 127.5, 70.1, 43.3, 17.8. The enantiomeric excess of (3*R*,4*E*)-3-hydroxyhex-4-enoic acid was determined by HPLC analysis on chiral column (Daicel, Chiralcel AD-H). Conditions: hexane/isopropanol = 95/5, flow rate = 1.0 mL/min, UV-Vis detection at λ_{max} = 210 nm, t_R = 12.178 min (major), 13.417 min (minor) (Figure S68).

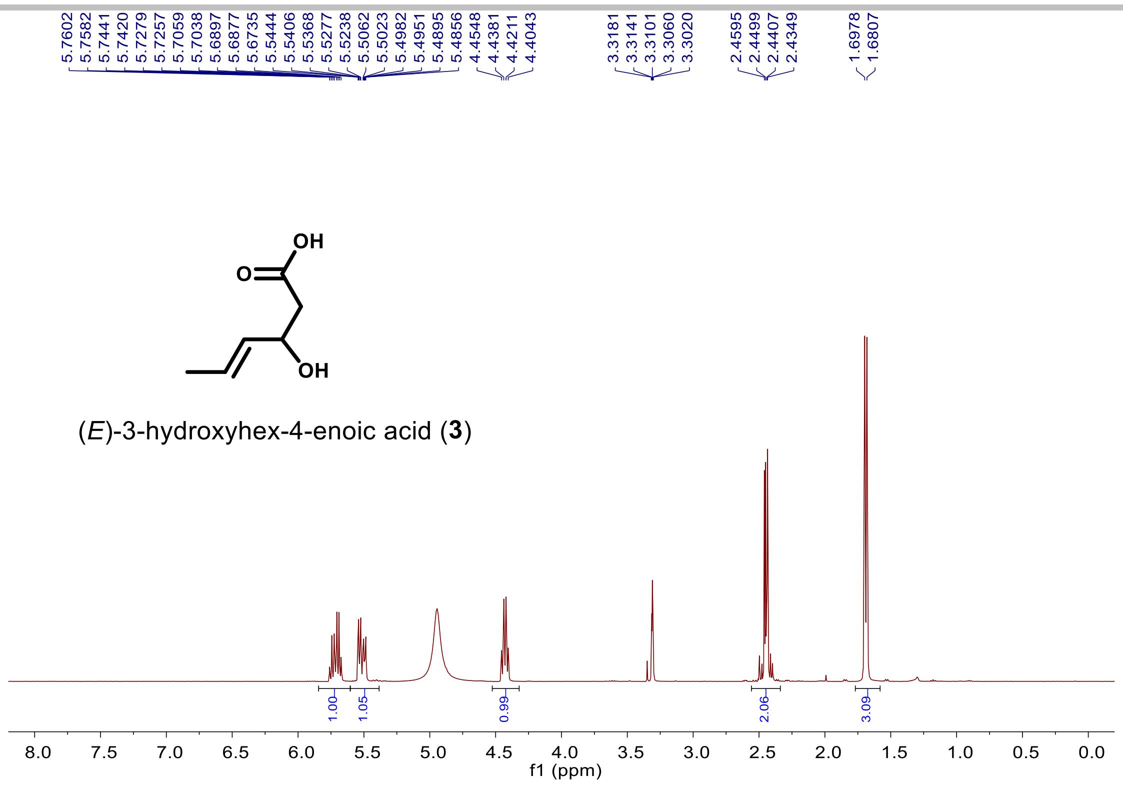

Figure S65. ^{1}H NMR spectrum of compound **3** (400 MHz, CD_3OD).

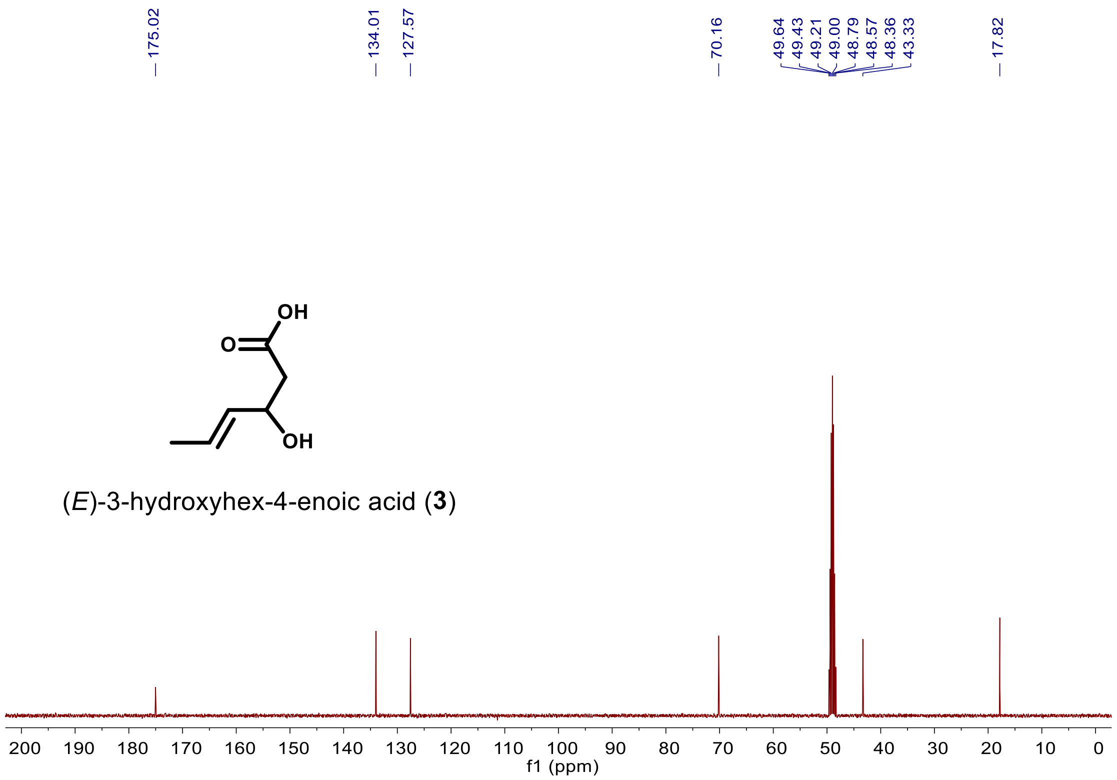

Figure S66. ^{13}C NMR spectrum of compound **3** (100 MHz, CD_3OD).

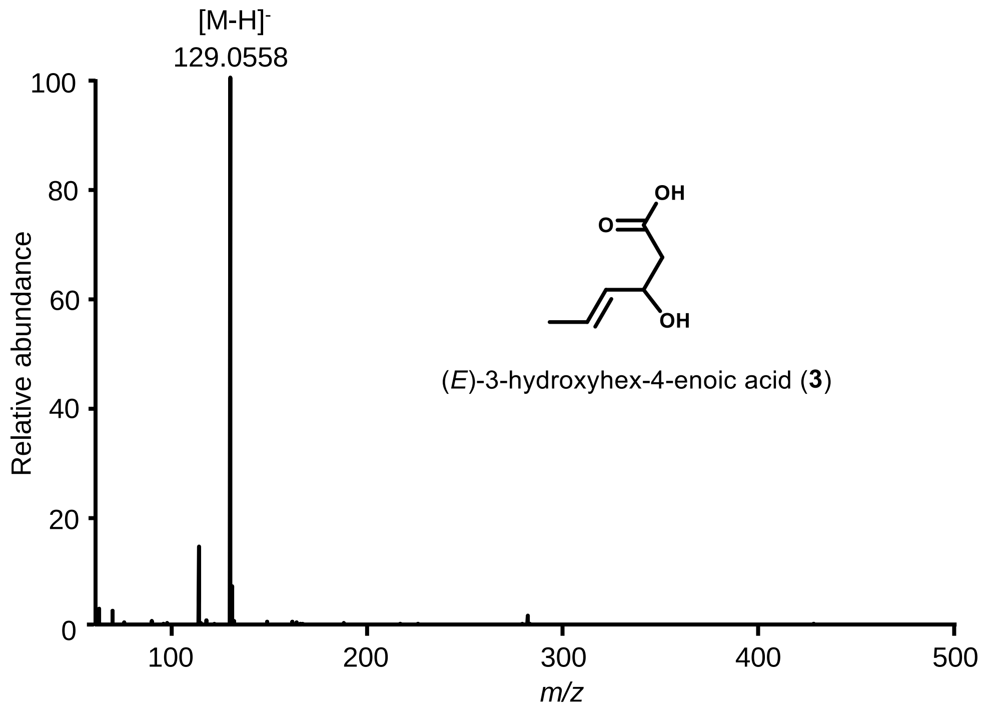

Figure S67. ESI-HRMS of compound **3**.

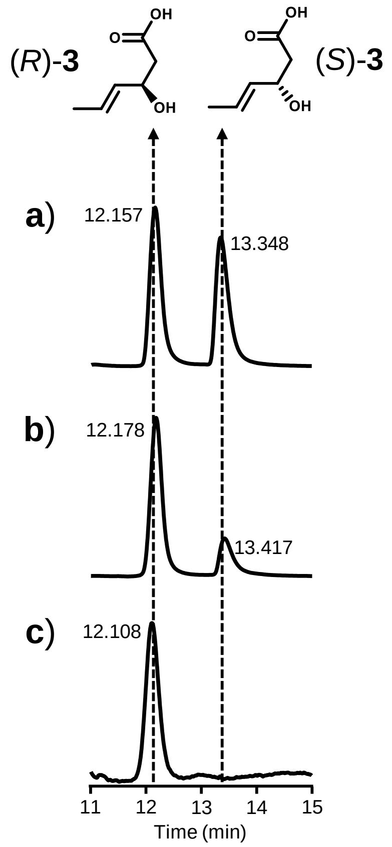

Figure S68. Confirmation of configuration of C3-hydroxy group in the product **3** from in vitro assay of *holo*-module 2 with synthetic **2** by chiral HPLC analysis. a) Synthetic racemic **3**. b) Synthetic enantiomerically enriched **3**. c) Product **3** from in vitro assay of *holo*-module 2 with synthetic **2**.

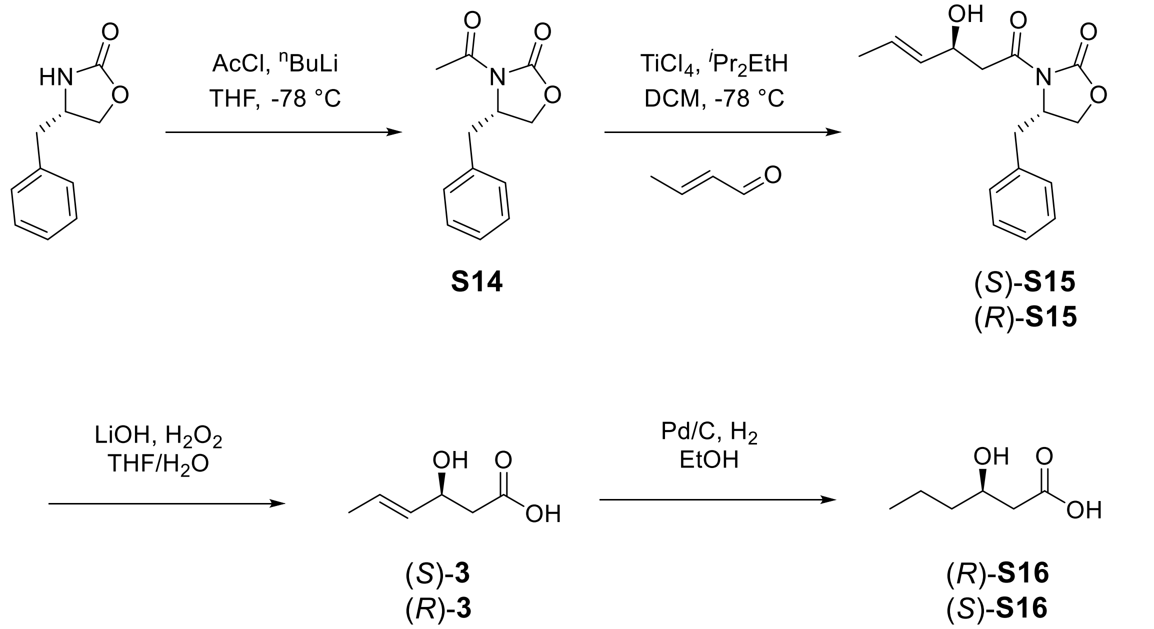

Scheme S3. Preparation of (*S*)-**3** and (*R*)-**3** by Evans' acyl oxazolidinone.

Synthesis of (*S*)-3-acetyl-4-benzyloxazolidin-2-one (S14).[11] To a (*S*)-4-Benzyl-2-oxazolidinone (3.30 g, 18.5 mmol) in THF (30 mL) was added ⁿBuLi (11.7 mL, 1.6 M in hexane, 18.7 mmol) dropwise at –78 °C under Ar. After 20 min, AcCl (1.65 g, 21.1 mmol) was added dropwise. The reaction mixture was allowed to stir at –78 °C for 2.5 h. The mixture was poured into a saturated aqueous solution of NH_4Cl (15 mL). After removal of THF under reduced pressure, the aqueous layer was extracted with CH_2Cl_2 (2 x 30 mL). The combined organic layers were washed with 10% NaOH solution and dried with $MgSO_4$, filtered and concentrated to dryness. The residue was puridied through silica gel column chromatography (cyclohexane/EtOAc, 5 : 1) to afford ethyl (*S*)-3-acetyl-4-benzyloxazolidin-2-one (**S14**)[11] as a white solid (2.80 g, 12.8 mmol, 69 % yield). ^{1}H NMR (500 MHz, $CDCl_3$): δ_H 7.33 (m, 2H), 7.28 (m, 1H), 7.21 (m, 2H), 4.67 (ddt, *J* = 9.6, 7.6, 3.2 Hz, 1H), 4.18 (m, 2H), 3.31 (dd, *J* = 13.4, 3.4 Hz, 1H), 2.78 (dd, *J* = 13.4, 9.6 Hz, 1H), 2.56 (s, 3H) ppm; ^{13}C NMR (125 MHz, $CDCl_3$): δ_C 190.8, 170.4, 153.8, 135.3, 129.6, 129.1, 127.5, 66.2, 55.1, 38.0, 24.0 ppm (Figures S69 and S70).

Synthesis of (*S*)-4-benzyl-3-((*S*,*E*)-3-hydroxyhex-4-enoyl)oxazolidin-2-one ((*S*)-S15) and (*S*)-4-benzyl-3-((*R*,*E*)-3-hydroxyhex-4-enoyl)oxazolidin-2-one ((*R*)-S15).[12] (*S*)-3-Acetyl-4-benzyloxazolidin-2-one (2.00 g, 9.1 mmol) was dissolved in CH_2Cl_2 (45 mL, –78 °C) under Ar. A solution of $TiCl_4$ (3.46 g, 18.2 mmol) in CH_2Cl_2 (18 mL) was added dropwise, followed by the addition of. diisopropylethylamine (2.35 g, 18.2 mmol). The resulting mixture was allowed to stir at –78 °C for 1 h, and then a solution of crotonaldehyde (1.28 g, 18.2 mmol) was added dropwise. Stirring was continued at –78 °C for 5 h and then allowed to warm to room temperature with continued stirring overnight. The mixture was poured onto a saturated aqueous solution of NH_4Cl (30 mL). The aqueous layer was extracted with CH_2Cl_2 (2 x 100 mL). The combined organic layers were washed with brine, dried with $MgSO_4$, filtered and concentrated to dryness. Purification of the crude product by column chromatography on silica gel (cyclohexane/EtOAc, 3 : 1) gave (*S*)-4-benzyl-3-((*S*,*E*)-3-hydroxyhex-4-enoyl)oxazolidin-2-one ((*S*)-**S15**) (685 mg, 2.4 mmol, 26 % yield) and (*S*)-4-benzyl-3-((*R*,*E*)-3-hydroxyhex-4-enoyl)oxazolidin-2-one ((*R*)-**S15**) (359 mg, 1.2 mmol, 14 % yield) as yellow solids.

(*S*)-4-Benzyl-3-((*S*,*E*)-3-hydroxyhex-4-enoyl)oxazolidin-2-one ((*S*)-**S15**).[13] ^{1}H NMR (500 MHz, C_6D_6): δ_H 7.04 (m, 3H), 6.85 (m, 2H), 5.72 (dqd, *J* = 15.4, 6.5, 1.3 Hz, 1H), 5.55 (ddq, *J* = 15.3, 5.9, 1.6 Hz, 1H), 4.68 (dq, *J* = 9.2, 4.2 Hz, 1H), 4.07 (ddt, *J* = 9.3, 8.0, 3.1 Hz, 1H), 3.42 (dd, *J* = 9.0, 2.8 Hz, 1H), 3.35 (dd, *J* = 16.8, 3.5 Hz, 1H), 3.14 (dd, *J* = 8.9, 8.0 Hz, 1H), 3.07 (dd, *J* = 16.8, 3.5 Hz, 1H), 2.87 (m, 2H), 2.24 (dd, *J* = 13.4, 9.3 Hz, 1H), 1.53 (dt, *J* = 6.5, 1.4 Hz, 3H) ppm; ^{13}C NMR (125 MHz, C_6D_6): δ_C 172.1, 153.3, 135.8, 133.2, 129.7, 129.0, 127.4, 126.3, 69.0, 65.7, 54.9, 43.3, 37.6, 17.7 ppm (Figures S71 and S72).

(*S*)-4-Benzyl-3-((*R*,*E*)-3-hydroxyhex-4-enoyl)oxazolidin-2-one ((*R*)-**S15**). ^{1}H NMR (500 MHz, C_6D_6): δ_H 7.04 (m, 3H), 6.85 (m, 2H), 5.71 (dqd, *J* = 15.4, 6.5, 1.3 Hz, 1H), 5.54 (ddq, *J* = 15.3, 6.0, 1.6 Hz, 1H), 4.70z (m, 1H), 4.05 (ddt, *J* = 9.5, 8.0, 3.1 Hz, 1H), 3.41 (dd, *J* =

8.9, 2.8 Hz, 1H), 3.23 (d, *J* = 3.26 Hz, 1H), 3.22 (d, *J* = 0.9 Hz, 1H), 3.10 (ddd, *J* = 8.9, 8.0, 0.7 Hz, 1H), 2.93 (dd, *J* = 13.4, 3.3 Hz, 1H), 2.74 (d, *J* = 4.5 Hz, 1H), 2.26 (dd, *J* = 13.4, 9.5 Hz, 1H), 1.53 (dt, *J* = 6.5, 1.4 Hz, 3H) ppm; ^{13}C NMR (125 MHz, C_6D_6): δ_C 172.1, 153.3, 135.8, 133.1, 129.6, 129.0, 127.4, 126.4, 68.9, 65.7, 54.9, 43.4, 37.8, 17.7 ppm (Figures S73 and S74). HRMS (APCI): $[M+H]^+$ calculated for $C_{16}H_{20}NO_4^+$ *m*/*z* 290.1387; found *m*/*z* 290.1385.

Synthesis of (*S*,*E*)-3-hydroxyhex-4-enoic acid ((*S*)-3). According to a known procedure,[14] (*S*)-4-benzyl-3-((*S*,*E*)-3-hydroxyhex-4-enoyl)oxazolidin-2-one ((*S*)-**S15**) (145 mg, 0.5 mmol) was dissolved in THF (2.1 mL) and H_2O (0.5 mL). The solution was cooled to 0 °C and a solution of H_2O_2 (35%, 0.21 mL, 18.2 mmol) was added dropwise. LiOH (36 mg, 1.5 mmol) was added and the resulting mixture was allowed to stir for 2 h, and then quenched by the addition of an aqueous solution of Na_2SO_3 (270 mg in 1.6 mL H_2O). After removal of THF under reduced pressure the aqueous phase was extracted with CH_2Cl_2 (10 mL), then acidified by the addition of 2 N HCl solution to pH 1, and extracted with Et_2O (3 x 10 mL). The combined organic layers were dried with $MgSO_4$, filtered and concentrated to yield (*S*,*E*)-3-hydroxyhex-4-enoic acid ((*S*)-**3**)[15] as a colorless oil (40 mg, 0.3 mmol, 61 % yield). Optical rotation: $[\alpha]_D^{25}$ = –7.5 (*c* 0.3, EtOH), lit. $[\alpha]_D^{25}$ = –22.2 (*c* 0.4, EtOH)];[15] ^{1}H NMR (500 MHz, C_6D_6): δ_H 5.45 (dqd, *J* = 15.3, 6.5, 1.3 Hz, 1H), 5.26 (ddq, *J* = 15.3, 6.3, 1.6 Hz, 1H), 4.29 (dddq, *J* = 8.4, 6.3, 4.1, 1.1 Hz, 1H), 2.32 (dd, *J* = 16.0, 8.4 Hz, 1H), 2.22 (dd, *J* = 16.0, 4.1 Hz, 1H), 1.43 (ddd, *J* = 6.5, 1.7, 1.0 Hz, 3H) ppm; ^{13}C NMR (125 MHz, C_6D_6): δ_C 176.9, 132.5, 126.8, 68.8, 41.7, 17.6 ppm (Figures S75 and S76).

Synthesis of (*R*,*E*)-3-hydroxyhex-4-enoic acid ((*R*)-3). Following the same procedure,[14] (*S*)-4-benzyl-3-((*R*,*E*)-3-hydroxyhex-4-enoyl)oxazolidin-2-one ((*R*)-**S15**) (145 mg, 0.5 mmol) was converted to yield (*R*,*E*)-3-hydroxyhex-4-enoic acid ((*R*)-**3**)[15] as a colorless oil (50 mg, 0.4 mmol, 77 % yield). Optical rotation: $[\alpha]_D^{25}$ = +12.5 (*c* 0.4, EtOH), lit. $[\alpha]_D^{25}$ = +22.5 (*c* 0.4, EtOH)] [15]

Synthesis of (*R*)-3-hydroxyhexanoic acid ((*R*)-S16). (*S*,*E*)-3-Hydroxyhex-4-enoic acid ((*S*)-**3**) (10 mg, 0.08 mmol) was dissolved in EtOH (5 mL). A catalytic amount of Pd/C (5%, 5 mg) was added and the atmosphere was exchanged to H_2 (10 bar). The reaction mixture was stirred for 1 h, the catalyst was filtered off, and the filtrate was concentrated to yield (*R*)-3-hydroxyhexanoic acid ((*R*)-**S16**)[12] as a colorless oil (10 mg, 0.08 mmol, 100 % yield). Optical rotation: $[\alpha]_D^{25}$ = –16.3 (*c* 0.3, $CHCl_3$), lit. $[\alpha]_D^{22}$ = –20.0 (*c* 1.0, $CHCl_3$);[12] ^{1}H NMR (500 MHz, $CDCl_3$): δ_H 4.05 (m, 1H), 2.58 (dd, *J* = 16.6, 3.0 Hz, 1H), 2.48 (dd, *J* = 16.6, 8.9 Hz, 1H), 1.46 (m, 2H), 0.95 (t, *J* = 7.0 Hz, 3H) ppm; ^{13}C NMR (125 MHz, $CDCl_3$): δ_C 177.0, 67.9, 41.0, 38.8, 18.8, 14.1 ppm (Figures S77 and S78).

Synthesis of (*S*)-3-hydroxyhexanoic acid ((*S*)-S16). Following the same procedure, (*R*,*E*)-3-hydroxyhex-4-enoic acid ((*R*)-**3**) (4 mg, 0.03 mmol) was converted into (*S*,*E*)-3-hydroxyhex-4-enoic acid ((*S*)-**S16**))[16] that was obtained as a colorless oil (4 mg, 0.03 mmol, 100 % yield). Optical rotation: $[\alpha]_D^{25}$ = +13.8 (*c* 0.5, $CHCl_3$), lit. $[\alpha]_D^{20}$ = +13.0 (*c* 0.5, $CHCl_3$).[16]

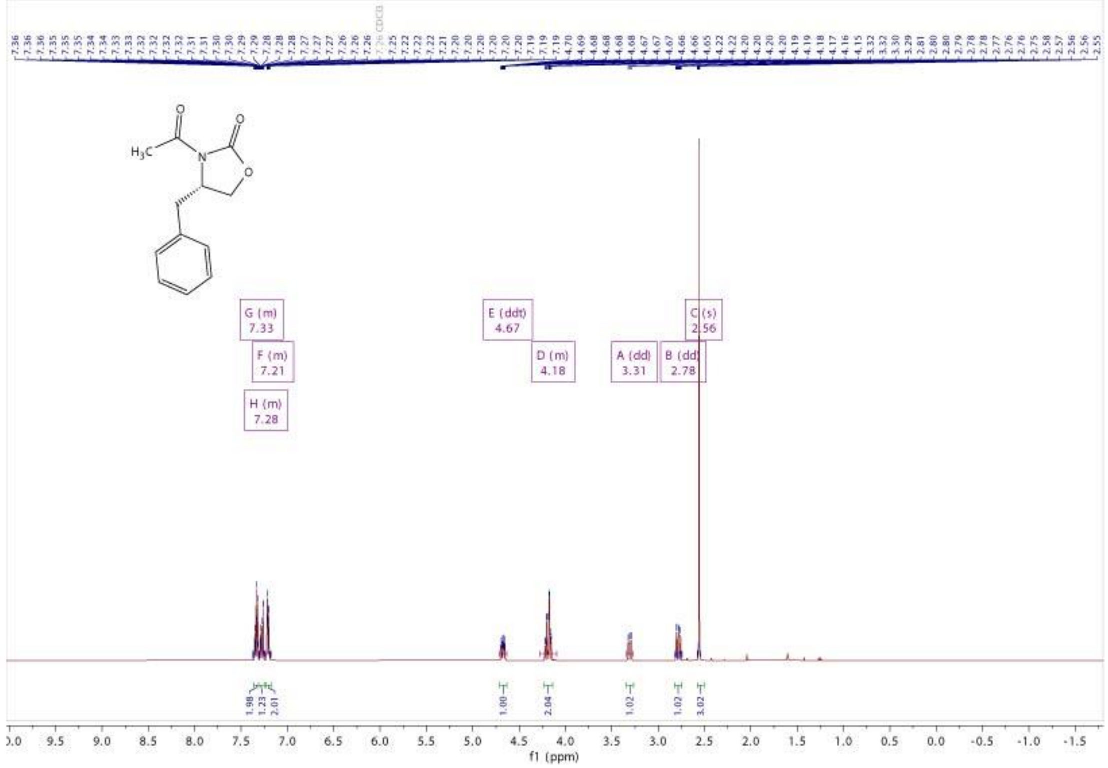

Figure S69. ^{1}H NMR spectrum of compound **S14** (500 MHz, $CDCl_3$).

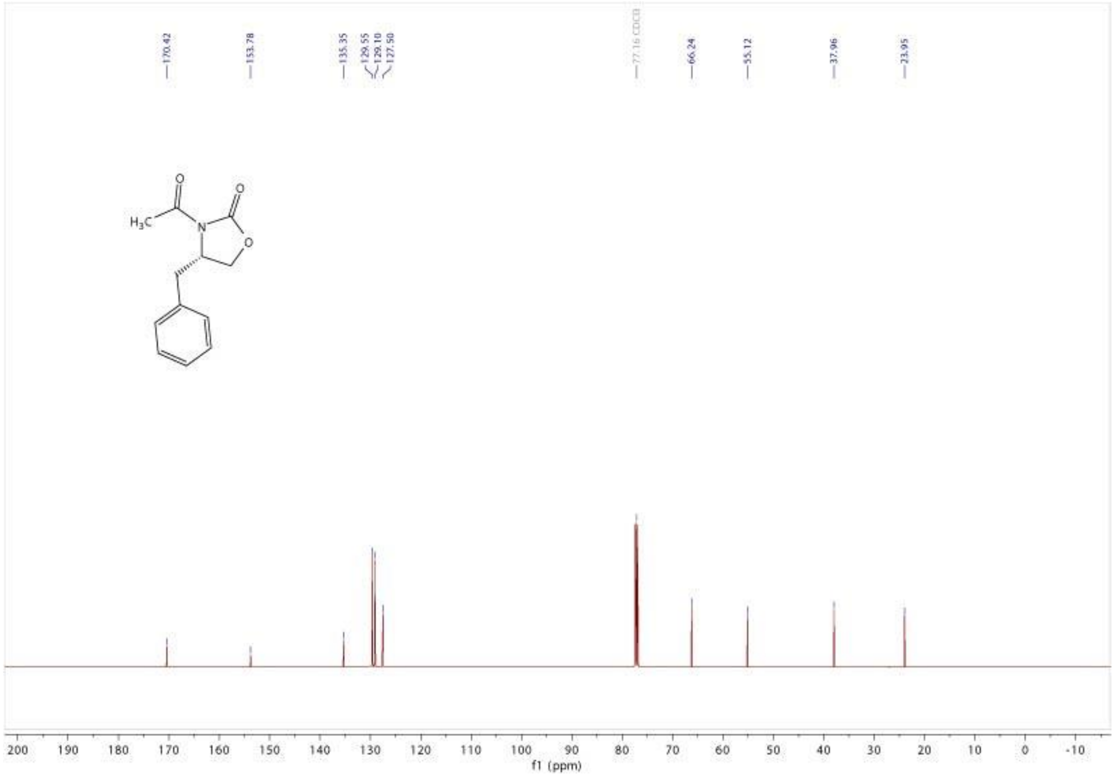

Figure S70. ^{13}C NMR spectrum of compound **S14** (125 MHz, $CDCl_3$).

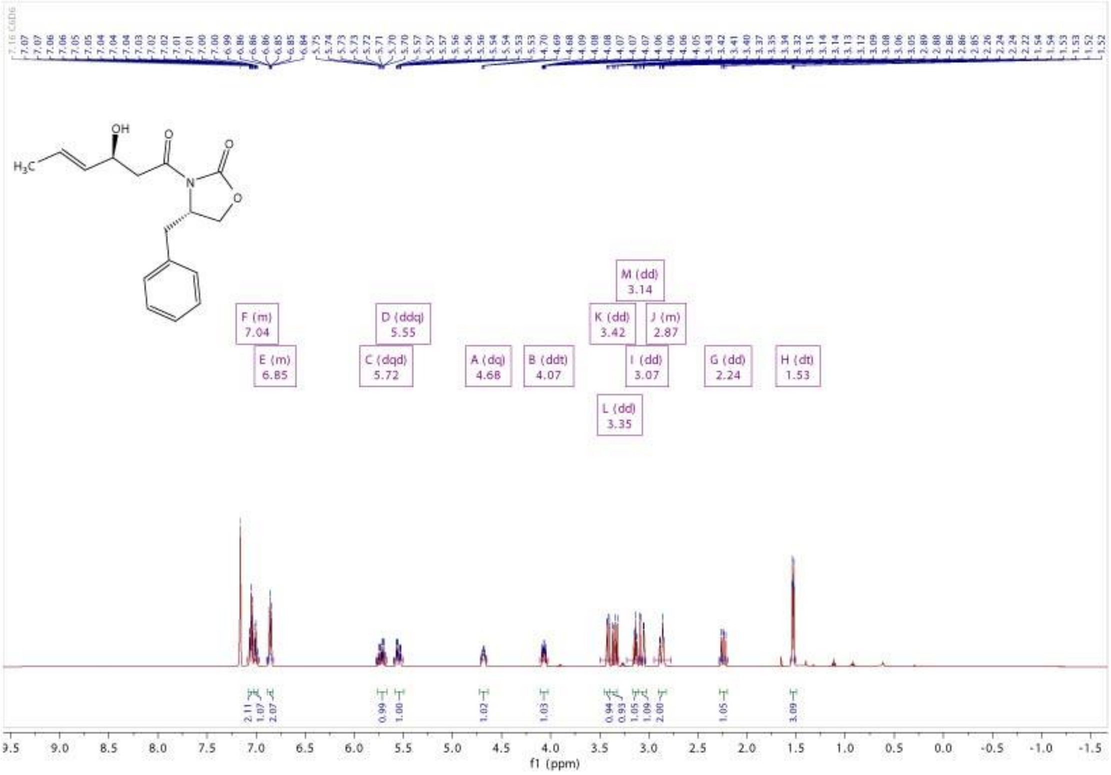

Figure S71. ^{1}H NMR spectrum of compound (*S*)-**S15** (500 MHz, C_6D_6).

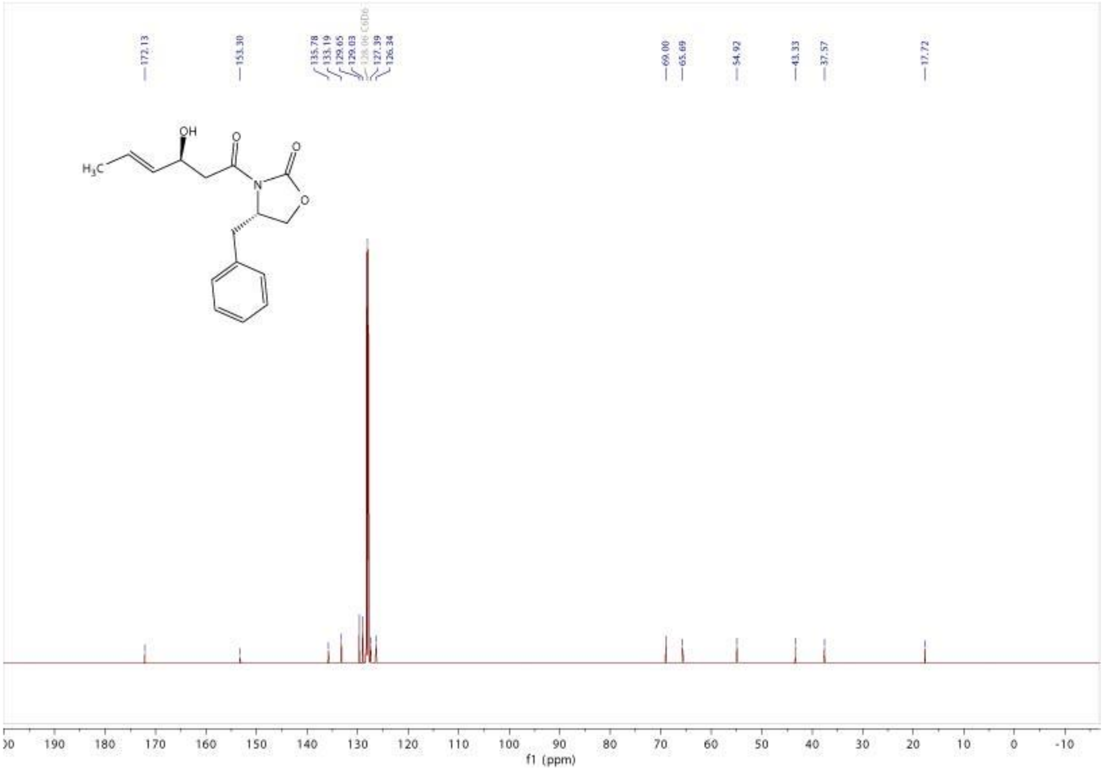

Figure S72. ^{13}C NMR spectrum of compound (*S*)-**S15** (125 MHz, C_6D_6).

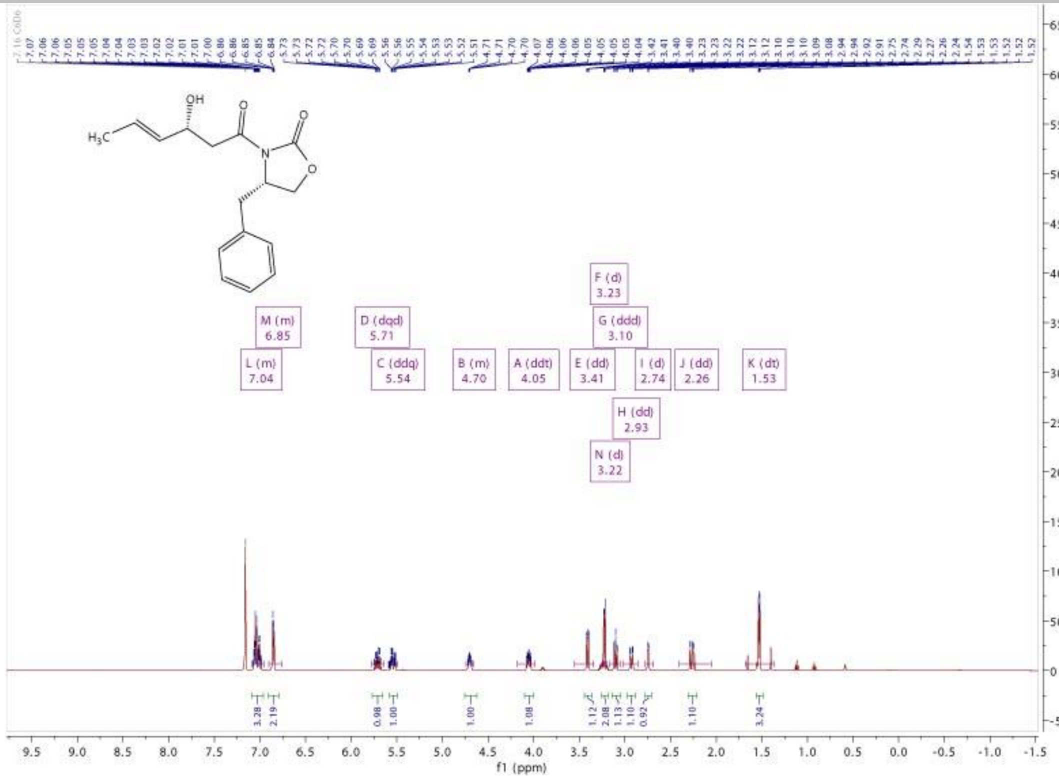

Figure S73. ^{1}H NMR spectrum of compound (*R*)-**S15** (500 MHz, C_6D_6).

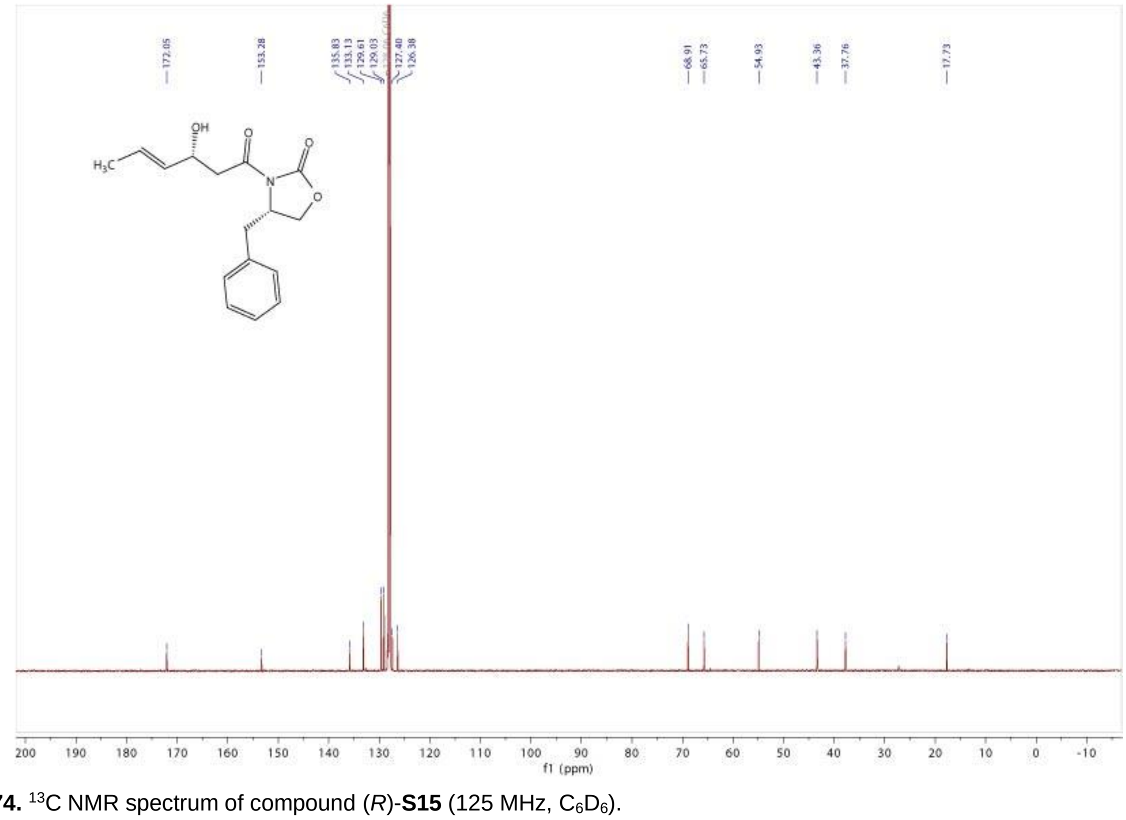

Figure S74. ^{13}C NMR spectrum of compound (*R*)-**S15** (125 MHz, C_6D_6).

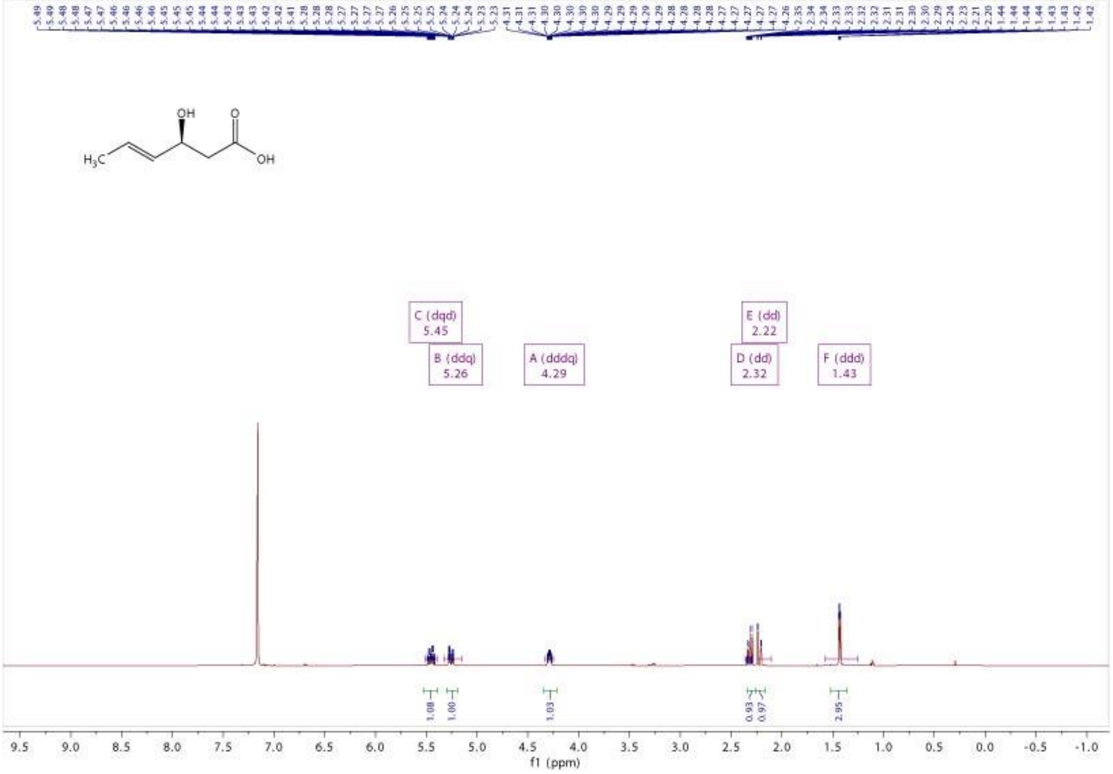

Figure S75. ^{1}H NMR spectrum of compound **3** (500 MHz, C_6D_6).

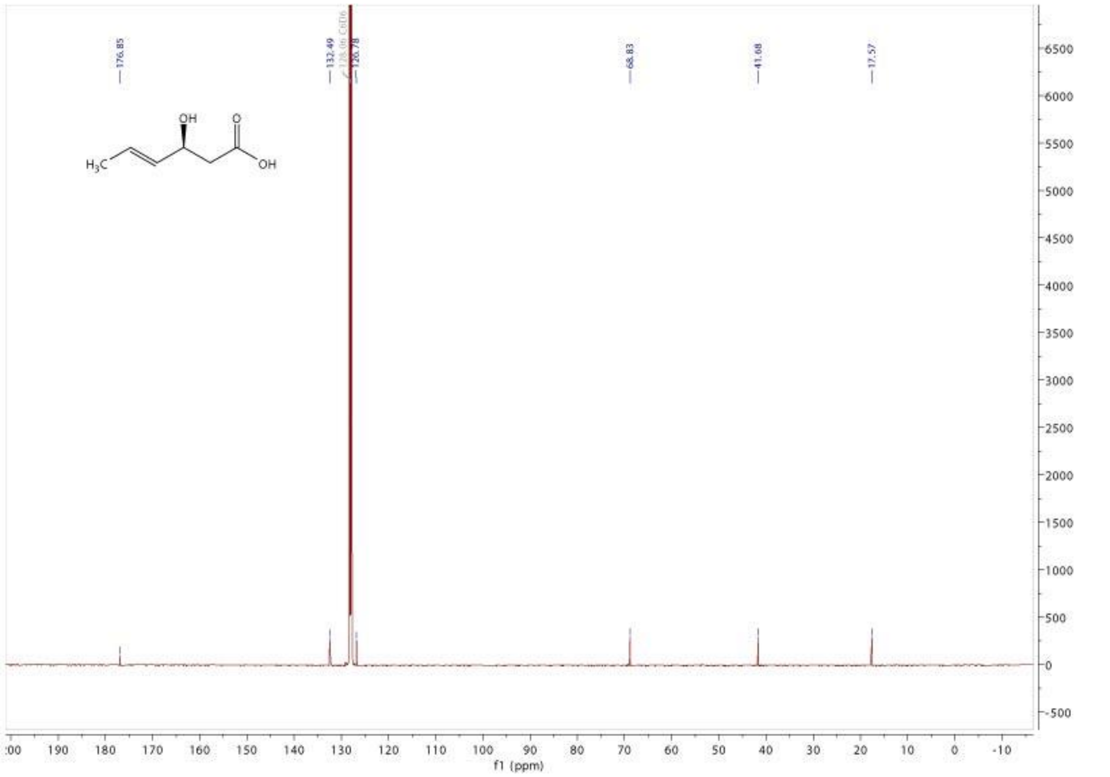

Figure S76. ^{13}C NMR spectrum of compound **3** (125 MHz, C_6D_6).

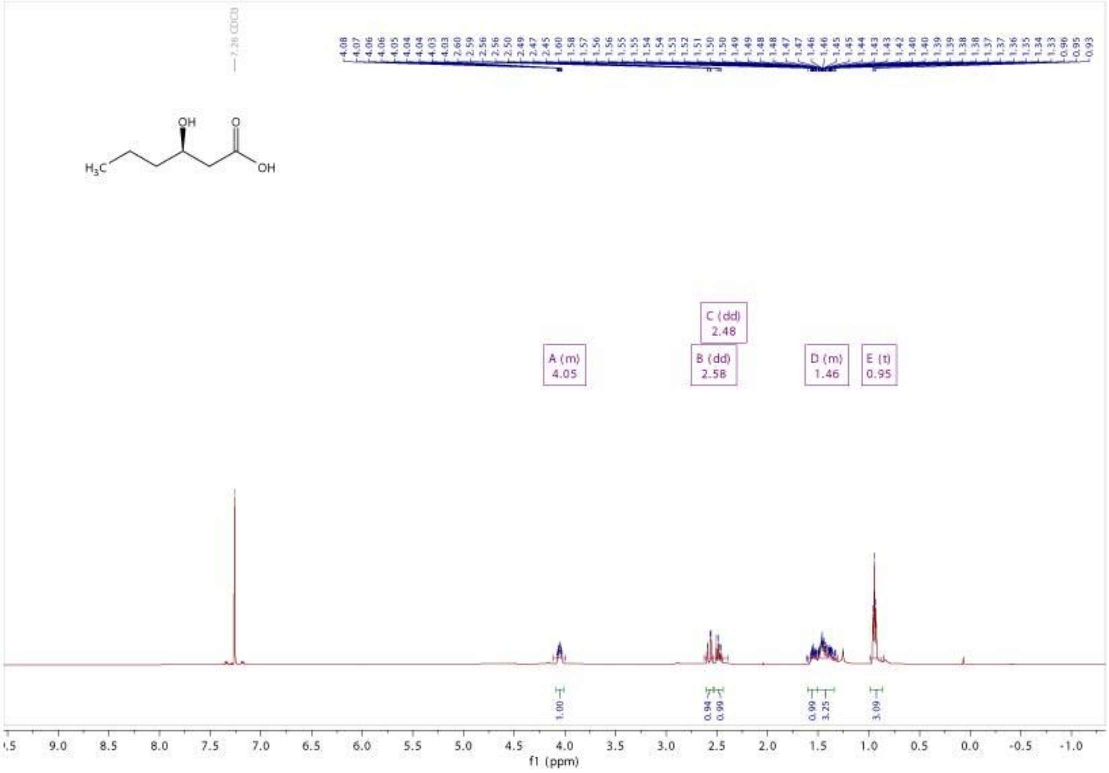

Figure S77. ^{1}H NMR spectrum of compound **S16** (500 MHz, $CDCl_3$).

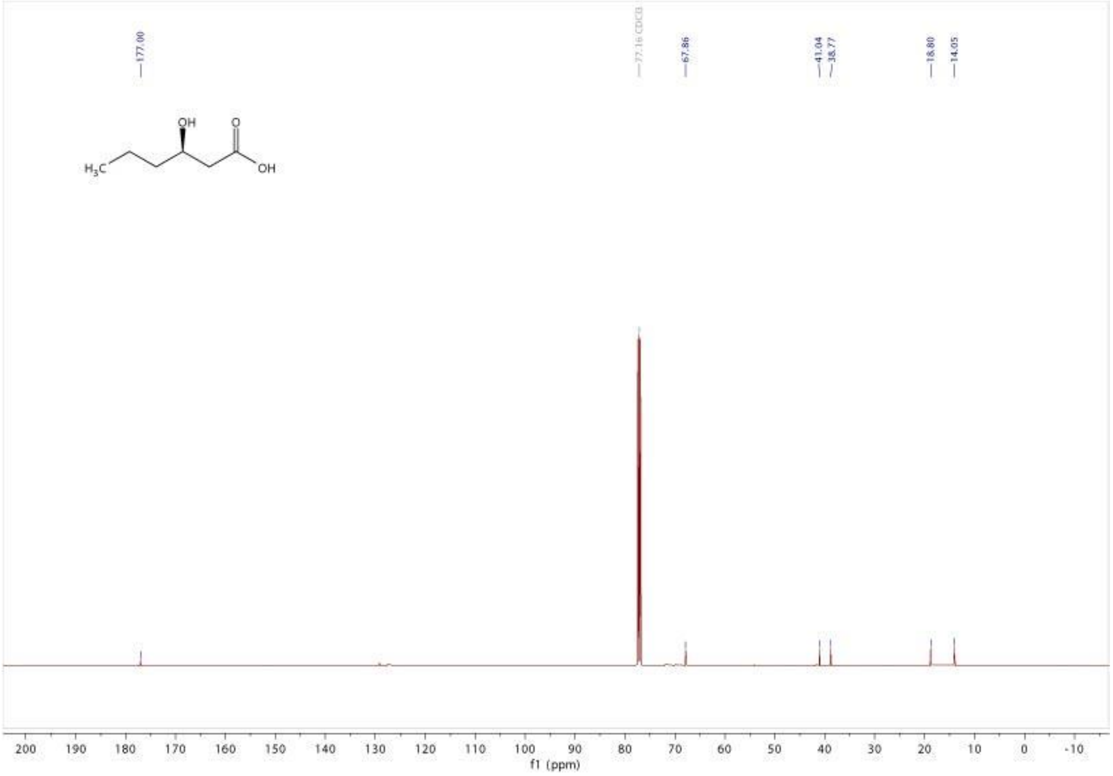

Figure S78. ^{13}C NMR spectrum of compound **S16** (125 MHz, $CDCl_3$).

References

[1] M. S. B. Paget, L. Chamberlin, A. Atrih, S. J. Foster, M. J. Buttner, *J. Bacteriol.* **1999**, *181*, 204–211.

[2] B. A. Pfeifer, S. J. Admiraal, H. Gramajo, D. E. Cane, C. Khosla, *Science* **2001**, *291*, 1790–1792.

[3] Y. Liu, X. Chen, Z. Li, W. Xu, W. Tao, J. Wu, J. Yang, Z. Deng, Y. Sun, *ACS Chem. Biol.* **2017**, *12*, 2589–2597.

[4] Y. Sun, X. He, J. Liang, X. Zhou, Z. Deng, *Appl. Microbiol. Biotechnol.* **2009**, *82*, 303–310.

[5] H. Qu, X. Gao, H. Zhao, Z. Wang, J. Yi, *Carbohydr. Polym.* **2019**, *223*, 115056.

[6] L. T. González, N. W. Minsky, L. E. Espinosa, R. S. Aranda, J. P. Meseguer, P. C. Pérez, *BMC Complement. Altern. Med.* **2017**, *17*, 39.

[7] L. Zuo, B. Jiang, Z. Jiang, W. Zhao, S. Li, H. Liu, B. Hong, L. Yu, L. Zuo, L. Wu, *J. Antibiot. (Tokyo)* **2016**, *69*, 835–838.

[8] M. Handa, K. A. Scheidt, M. Bossart, N. Zheng, W. R. Roush, *J. Org. Chem.* **2008**, *73*, 1031–1035.

[9] K. Nishihara, M. Kanemori, M. Kitagawa, H. Yanagi, T. Yura, *Appl. Environ. Microbiol.* **1998**, *64*, 1694–1699.

[10] Y. Wang, R. Takeyama, Y. Kobayashi, *Angew. Chem. Int. Ed.* **2006**, *45*, 3320–3323.

[11] A. May, P. Willoughby, T. Hoye, *J. Org. Chem.* **2008**, *73*, 3292–3294.

[12] S. Le, D. Muñoz, N. Saunders, T. Simpson, D. Smith, F. Soulas, C. Willis, *Org. Biomol. Chem.* **2005**, *3*, 1719–1728.

[13] J. Son, M. Hwang, W. Lee, D. Lee, *Org. Lett.* **2007**, *9*, 3897–3900.

[14] M. Martín, R. Rodriguez-Acebes, Y. Garcia-Ramos, V. Martínez, C. Murcia, I. Digón, I. Marco, M. Pelay-Gimeno, R. Fernández, F. Reyes, A. Francesch, S. Munt, J. Tulls- Puche, F. Albericio, C. Cuevas, *J. Am. Chem.Soc.* **2014**, *136*, 6754–6762.

[15] T. Yan, A. Hung, H. Lee, C. Chang, W. Liu, *J. Org. Chem.* **1995**, *60*, 3301–3306.

[16] S. Smith, M. Uteuliyev, J. Takacs, *Chem. Commun.* **2011**, *47*, 7812–7814.

Author Contributions

M.L., Y. D. and L.T. performed the genetic and enzymatic assay. H.X., K.S., J. L. and Z.Y. synthesised the substrates. M.Q. and J.X. performed hepatoprotective experiment. G.S. and Z.D. analyzed the data and revised the manuscript. Y.S. and J.S.D. conceived the overall project, analyzed the data and wrote the manuscript.
